# Comprehensive Analyses Identify APOBEC3A as a Genomic Instability-Associated Immune Prognostic Biomarker in Ovarian Cancer

**DOI:** 10.3389/fimmu.2021.749369

**Published:** 2021-10-21

**Authors:** Fangfang Xu, Tingwei Liu, Zhuonan Zhou, Chang Zou, Shaohua Xu

**Affiliations:** ^1^ Department of Gynecology, Shanghai First Maternity and Infant Hospital, School of Medicine, Tongji University, Shanghai, China; ^2^ Jianping Educational Center of International Curriculum, Shanghai Jianping High School, Shanghai, China

**Keywords:** APOBEC3A, ovarian cancer, tumor microenvironment, genomic instability, immunotherapy

## Abstract

Ovarian cancer (OC) is one of the most malignant tumors whose mortality rate ranks first in gynecological tumors. Although immunotherapy sheds new light on clinical treatments, the low response still restricts its clinical use because of the unique characteristics of OC such as immunosuppressive microenvironment and unstable genomes. Further exploration on determining an efficient biomarker to predict the immunotherapy response of OC patients is of vital importance. In this study, integrative analyses were performed systematically using transcriptome profiles and somatic mutation data from The Cancer Genome Atlas (TCGA) based on the immune microenvironment and genomic instability of OC patients. Firstly, intersection analysis was conducted to identify immune-related differentially expressed genes (DEGs) and genomic instability-related DEGs. Secondly, Apolipoprotein B MRNA Editing Enzyme Catalytic Subunit 3A (APOBEC3A) was recognized as a protective factor for OC, which was also verified through basic experiments such as quantitative reverse transcription PCR (RT-qPCR), immunohistochemistry (IHC), Cell Counting Kit-8 (CCK-8), and transwell assays. Thirdly, the correlation analyses of APOBEC3A expression with tumor-infiltrating immune cells (TICs), inhibitory checkpoint molecules (ICPs), Immunophenoscores (IPS), and response to anti-PD-L1 immunotherapy were further applied along with single-sample GSEA (ssGSEA), demonstrating APOBEC3A as a promising biomarker to forecast the immunotherapy response of OC patients. Last, the relationship between APOBEC3A expression with tumor mutation burden (TMB), DNA damage response (DDR) genes, and m6A-related regulators was also analyzed along with the experimental verification of immunofluorescence (IF) and RT-qPCR, comprehensively confirming the intimate association of APOBEC3A with genomic instability in OC. In conclusion, APOBEC3A was identified as a protective signature and a promising prognostic biomarker for forecasting the survival and immunotherapy effect of OC patients, which might accelerate the clinical application and improve immunotherapy effect.

## Introduction

Ovarian cancer (OC) is a common malignant tumor in gynecology whose morbidity comes fourth after breast cancer, cervical cancer, and endometrial cancer among the cancers in women, which remains a challenging global health problem. Moreover, the mortality rate of OC ranks first in gynecological tumors according to epidemiological analysis. In 2020, there were 313,959 new cases and 207,252 deaths of OC around the world (1). In 2018, the CONCORD project on the prevalence of OC in 61 countries worldwide indicated that the 5-year survival rate of OC patients worldwide from 2010 to 2014 was 30%–50% (2). Statistically, the prognosis of early OC patients is satisfactory, whose 5-year survival rate can reach 90%; while the 5-year survival rate of advanced OC is significantly reduced to only 20%–30% (2, 3). However, early diagnosis of OC is quite difficult and more than 70% of patients are found to be already in advanced stage III or IV when they first get diagnosed; by then, the treatment options are also limited and the prognosis is not ideal (4–6). The pathogenesis of OC is not yet clear, and it is currently believed to involve endocrine, genetic changes, microbial infections, stress, etc. (7–9). Though there were plenty of hypotheses on the tumorigenesis and progression of OC, the exact mechanisms remain confusing and indistinct. The current treatments for OC are still surgery, chemotherapy, targeted therapy, hormone therapy, and immunotherapy, which are still expected to carry out more effortful researches to prevent the resistance and recurrence of OC (10). At present, immunotherapy, especially immune checkpoint blockade (ICB) therapy, has achieved an impressive success in melanoma, non-small cell lung cancer and other cancers (11, 12), but the therapeutic value of immunotherapy in OC is still in the research stage (13–15). Disappointedly, the response rate to ICB therapy of OC patients appears unsatisfactory yet. Hence, it is imminently needed to explore some novel biomarkers to recognize OC as early as possible and enhance the effect of immunotherapy in OC patients, therefore clarifying the possible molecular mechanisms of OC.

Tumor microenvironment (TME) includes not only the tumor itself and the matrix, but also immune components like tumor-associated macrophages (TAMs), CD8^+^ T lymphocytes, and myeloid-derived suppressor cells (16). Many previously published literatures have indicated that the components of TME are dynamically changing and related to a variety of biological behaviors of cancers such as invasion, metastasis, and immune escape of immunological surveillance (17, 18). So far, according to the status of tumor-infiltrating immune cells (TICs) in TME, tumors can be divided into two different types: hot tumors and cold tumors (19, 20). Hot tumor possesses a high density of CD8^+^ T lymphocytes in tumor tissue whose function is weakened by immunosuppressive molecules. Patients with this type of tumor can benefit from ICB treatment. Oppositely, cold tumor lacks T lymphocytes, and this type of patients may get benefit from changing the number of TICs (21). Although the tumor mutation burden (TMB) of OC is relatively high, it still belongs to the category of cold tumors, that is, there is a general lack of cytotoxic T lymphocyte (CTL) infiltration, and the infiltrating T lymphocytes cannot recognize all of the tumor antigens (22). Additionally, an important reason for limiting the immunotherapy response of OC is that the TME of it always maintains in a state of immunosuppression. Therefore, it allows no delay to focus on the genomic characteristics of tumor immune microenvironment (TIME) and explore some effective biological signatures to convert OC from cold tumor into hot tumor, thus improving the effect of immunotherapy.

Accumulating evidence has expounded genomic instability as a hallmark of cancer (23, 24). Moreover, genomic instability has also been reported as a crucial prognostic signature, which is strongly related to the tumorigenesis and progression (25, 26). As for OC, there are few researches to investigate the relationship between genomic instability-related genes and clinical characteristics. Although germline mutations in BRCA1 and BRCA2 (BRCA1/2) genes considerably elevate genomic instability (27), the molecular mechanisms of genomic instability remain only partially understood. More interestingly, a variety of previous published studies have showed that the cumulative genomic instability could generate tumor neoantigens, which might activate the immune infiltrating cells and cause spontaneous antitumor immunological effect, thus forecasting the response to immunotherapy (28–31). Consequently, there is an inevitable connection between genome instability and TIME, which could be possible to predict the prognosis and immunotherapy response of cancer. However, biological signatures that are both associated with genomic instability and TIME in OC patients are still seldomly analyzed and remain to be explored systematically.

In this study, we tried to explore some novel biomarkers through the gene expression profiles and somatic mutation data comprehensively by integrative bioinformatics methods and basic experiments. In this article, Apolipoprotein B MRNA Editing Enzyme Catalytic Subunit 3A (APOBEC3A) was finally recognized as a prognostic signature that was correlated with both genomic instability and TIME. Simultaneously, we conducted Gene Set Enrichment Analysis (GSEA) and single-sample GSEA (ssGSEA) on the basis of the different APOBEC3A expression groups. Additionally, the relationship between APOBEC3A expression and ImmuneScore, TICs, inhibitory checkpoint molecules (ICPs), Immunophenoscores (IPS), and anti-PD-L1 therapy response was further performed, indicating that APOBEC3A was apparently a favorable signature for improving the immunosuppressive microenvironment and foretelling the immunotherapy responsiveness of OC. Besides, the correlation of APOBEC3A expression with TMB, DNA damage response (DDR) genes, somatic mutation, and m6A regulators was analyzed as well, indicating that APOBEC3A could also be an indicator of genomic instability. The above analyses showed that APOBEC3A could serve as a vital signature for instructing the clinical therapeutical strategies and forecasting the clinical prognoses. Besides, we also conducted a variety of basic experiments to confirm APOBEC3A as a protective factor, such as quantitative reverse transcription PCR (RT-qPCR), immunohistochemistry (IHC), Cell Counting Kit-8 (CCK-8) assay, 5-Ethynyl-20-deoxyuridine (EdU) staining, cell cycle detection, transwell assay, and immunofluorescence (IF). This study is aimed at identifying a promising biomarker that targets mainly on the TIME and genomic instability, thus improving the immunosuppressive microenvironment of OC, illuminating the biomolecular mechanisms, and providing new therapeutical targets for the clinical treatments.

## Materials and Methods

### Data Collection

**Figure 1:**
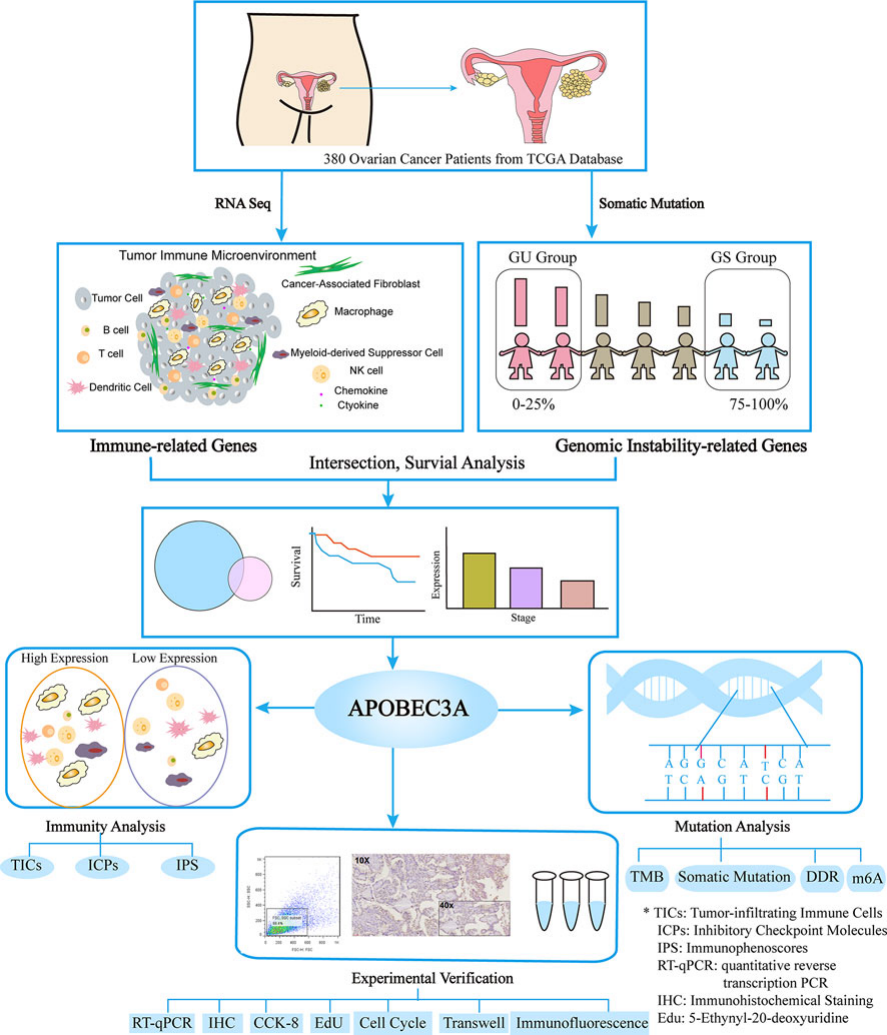
The analysis workflow of this study.

Data preparation, processing, general analysis, and verification of basic experiments in this study are displayed in the flow chart (**Figure 1**). Primarily, transcriptome data, somatic mutation profiles, and clinical characteristics of 380 OC tumor cases were extracted from The Cancer Genome Atlas (TCGA) databases (https://portal.gdc.cancer.gov/). Besides, we further downloaded the gene expression profiles and corresponding clinical features of OC patients from the International Cancer Genome Consortium (ICGC) OV-AU (Ovarian cancer-Australia) database to verify the survival results in our study (https://icgc.org/). The clinical information of OC patients in TCGA and ICGC datasets are displayed in **Table S1**. Then, KM Plotter online database (http://kmplot.com) was exploited to analyze the prognostic value of gene expression in OC. The OC cases were divided equally into two different expression groups to analyze the overall survival (OS). In order to demonstrate the prognostic value for immunotherapy, we ultimately included an immunotherapeutic dataset called IMvigor210 cohort from Mariathasan et al. in this study, which contained the intervention information of treatment using anti-PD-L1 antibody in urothelial cancer patients (32).

### Identification of Immune-Related Genes and Genomic Instability-Related Genes

ESTIMATE package with R software (version 4.0.5) was applied to determine the proportion of immune, stromal, and immune-stromal components in TME of each OC patient. Each component was presented by means of ImmuneScore, StromalScore, and ESTIMATEScore, respectively (33). The higher the score, the higher the proportion. Then, 380 OC samples were separated equally into two cohorts (high-ImmuneScore group and low-ImmuneScore group) grounded on the median value of ImmuneScore. Additionally, immune-related differentially expressed genes (DEGs) were recognized *via* “limma” package through the comparison of high-ImmuneScore with low-ImmuneScore cohorts, with the filtering quality of false discovery rate (FDR) < 0.05 and absolute value of log2 fold change (FC) > 0.58. Simultaneously, gene expression profiles and somatic mutation data were combined into a tumor genome to recognize genomic instability-related DEGs and the following steps were conducted in turn: (i) the cumulative amount of somatic mutations per OC sample was calculated and then sorted in descending order, which meant the higher the total number of somatic mutations, the more the chromosomal mutation sites; (ii) the genomic unstable (GU) cohort contains the top 25% of samples while the genomic stable (GS) cohort contains the last 25%; (iii) genomic instability-related DEGs were identified based on the comparison of the transcriptome information between the GU and GS cohorts using “limma” package (absolute value of log2 FC > 0.58 and FDR < 0.05).

### Survival Analysis and Correlation Analysis of Gene Expression With Clinicopathological Characteristics

R package “survival” and “survminer” were loaded to perform survival analysis (34). The Kaplan–Meier method was taken to evaluate the survival rate, and the log-rank test was simultaneously employed to analyze the survival difference by comparing different expression cohorts (high- vs. low-expression groups) with *p*-value < 0.05. In this study, 361 OC samples were picked out from the total of 380 TCGA patients *via* the following exclusion criteria: (i) exclude the samples whose survival time is less than 30 days; and (ii) exclude the samples with incomplete clinical characteristics. The 93 OC patients from ICGC were also selected according to the above filtering standards. Additionally, the package “ggpubr” was employed to analyze the relationship between gene expression and clinicopathological features, taking Kruskal–Wallis rank sum test as the method to assess the statistical significance. Moreover, the KM Plotter online database (GSE15622 of 35 samples and GSE14764 of 80 samples) was also employed to verify the correlation of gene expression with survival rate in OC.

### Functional Enrichment Analysis and Gene Set Enrichment Analysis

Functional enrichment analysis of DEGs was conducted to determine the significantly enriched Gene Ontology (GO) terms through R packages “clusterProfiler”, “enrichplot”, and “ggplot2”. The terms satisfying the standards (both *p*-value < 0.05 and *q*-value < 0.05) were considered as significant. Moreover, the HALLMARK gene sets and C2, CP, and KEGG.v7.2 symbols were performed between different gene expression cohorts through GSEA using the gsea software (version: 4.0.3). The gene sets were thought to be significant with the level of 5% (NOM *p*-value and FDR *q*-value).

### Immunity Analysis

At the same time, R package CIBERSOT (35, 36) and ssGSEA (37) algorithms were occupied to determine TICs proportion of all OC samples and cell immune responses between high- and low-expression cohorts. Additionally, ssGSEA using “GSVA” (R package) was specifically employed to calculate the proportion of TICs among different expression groups and assess immune-related biological functions as well. The immune gene sets were acquired from the MSigDB database (https://www.gsea-msigdb.org/). Moreover, ICPs were extracted from the previously published articles, whose correlation with gene expression was further analyzed with *p*-value < 0.05. Besides, IPS of OC patients was retrieved from the Cancer Immunome Database (TCIA, https://tcia.at/home) and its relationship with gene expression was then assessed with the significance quality of *p*-value < 0.05. Moreover, the IMvigor210 cohort was selected in our study to further verify the prognostic value of the biomarker for immunotherapy response, similarly with *p*-value < 0.05 as the significance quality.

### Mutation Analysis

Somatic mutation data of OC samples was stored in the MAF form and differentially mutated genes were simultaneously determined between the different expression cohorts *via* MAFTOOLS package (38). The TMB score of each OC case was computed according to the following formula: (total mutation/total covered bases) × 10^6^ (39). DDR genes and m6A-related regulators were gathered from many published literatures and the association of these regulators with gene expression was analyzed further (40–44).

### Clinical Specimens

Five OC specimens and five normal ovarian tissues were provided by Shanghai First Maternity and Infant Hospital, which were then collected and conserved in liquid nitrogen before use. The diagnoses of acquired samples were all carefully certified and checked by experienced pathologists, which were consistent with the diagnostic principles of the latest World Health Organization Classification. All specimens have obtained the informed consents with permission of Medical Ethics Committee of Shanghai First Maternity and Infant Hospital. Half of the tissues were stored in liquid nitrogen and the other half were fixed in formalin and embedded in paraffin for histological analysis.

### Cell Culture, RNA Extraction, and Real-Time qPCR

Human OC cell lines HEY and the human leukemic cell line, THP-1 cells were purchased from ATCC (Manassas, VA, USA). Then, cells were carefully preserved using RPMI 1640 medium (Servicebio, China) replenished with 10% fetal bovine serum (FBS, Biological Industries, Israel) and 1% penicillin/streptomycin (New Cell & Molecular Biotech Co, China) in a humid incubator containing 5% CO_2_ at 37°C. The THP-1 cells (5×10^4^ cells/100 μl) were seeded in six-well plates (Corning, USA) and differentiated into macrophages by adding 100 ng/ml of phorbol-12-myristate-13-acetate (PMA, MCE, China) for 48 h. After THP-1 cells were differentiated into M0 macrophages, macrophages were treated with 100 ng/ml lipopolysaccharide (LPS) (Peprotech, USA) for 48 h to differentiate into M1 macrophages. A transwell device (Corning, USA) with a 0.4-μm porous membrane was used for coculture treatments. HEY cells were seeded onto the upper chamber of the Transwell apparatus while M0 and M1 macrophages were seeded at a density of 2 × 10^5^ per well of the six-well plate. After 24 h of co-culture, the macrophages were collected for the succeeding analysis.

Additionally, total RNA from clinical tissues and cells was extracted by TRIzol (Invitrogen, USA), which was further reversely transcribed into cDNA using 5X ALL-IN-One RT Master Mix kit (Applied Biological Materials Inc, Canada) after assessing the purity and concentration. Moreover, real-time PCR was conducted by TB Green Premix Ex Taq kit (Takara, Japan) and GAPDH served as the internal control for all PCR reactions. As shown in **Table 1**, the primers used in this study were exhibited.

**Table 1:** Primer nucleotide sequence of this study.

Gene	Primer nucleotide sequence
GAPDH	Forward: 5’-CTGGGCTACACTGAGCACC-3’
	Reverse: 5’-AAGTGGTCGTTGAGGGCAATG-3’
APOBEC3A	Forward: 5’-CACAACCAGGCTAAGAATCTTCTC-3’
	Reverse: 5’-CAGTGCTTAAATTCATCGTAGGTC-3’
CXCL11	Forward: 5’-TGTGCTACAGTTGTTCAAGGCTTCC-3’
	Reverse: 5’-CTTGCTTGCTTCGATTTGGGATTTAGG-3’
IL1B	Forward: 5’-CCACAGACCTTCCAGGAGAATG-3’
	Reverse: 5’-GTGCAGTTCAGTGATCGTACAGG-3’
CD80	Forward: 5’-CTCTTGGTGCTGGCTGGTCTTT-3’
	Reverse: 5’-GCCAGTAGATGCGAGTTTGTGC-3’
PD-L1	Forward: 5’-TGGCATTTGCTGAACGCATTT-3’
	Reverse: 5’-TGCAGCCAGGTCTAATTGTTTT-3’
LAG3	Forward: 5’-GCGGGGACTTCTCGCTATG-3’
	Reverse: 5’-GGCTCTGAGAGATCCTGGGG-3’
CTSS	Forward: 5’-TGTAGATGCGCGTCATCCTTC-3’
	Reverse: 5’-CCAACCACAAGTACACCATGAT-3’
PDCD1LG2	Forward: 5’-ACCCTGGAATGCAACTTTGAC-3’
	Reverse: 5’-AAGTGGCTCTTTCACGGTGTG-3’
RAD51	Forward: 5’-CAACCCATTTCACGGTTAGAGC-3’
	Reverse: 5’-TTCTTTGGCGCATAGGCAACA-3’
FEN1	Forward: 5’-ATGACATCAAGAGCTACTTTGGC-3’
	Reverse: 5’-GGCGAACAGCAATCAGGAACT-3’
BRCA2	Forward: 5’-CACCCACCCTTAGTTCTACTGT-3’
	Reverse: 5’-CCAATGTGGTCTTTGCAGCTAT-3’
POLB	Forward: 5’-TGGAAAAGATTCGGCAGGATG-3’
	Reverse: 5’-CAGATGGACCAATGCCACTAAC-3’
BRIP1	Forward: 5’-CTTACCCGTCACAGCTTGCTA-3’
	Reverse: 5’-CACTAAGAGATTGTTGCCATGCT-3’
XRCC4	Forward: 5’-ATGTTGGTGAACTGAGAAAAGCA-3’
	Reverse: 5’-GCAATGGTGTCCAAGCAATAAC-3’

### CCK-8 Assay, EdU Incorporation Assay, Cell Cycle Detection, and Transwell Assay

APOBEC3A overexpression plasmids were ordered from Public Protein/Plasmid Library. HEY cells were transfected for 48 h with APOBEC3A overexpression plasmids (APOBEC3A-pcDNA3.1, 700 ng/μl) and control plasmids (pcDNA3.1, 500 ng/μl). Then, we followed the instructions of manufacturer and transfected both plasmids into HEY cells using Lipofectamine 2000 reagent (Invitrogen, USA). The transfection efficiency was verified by RT-qPCR after 48 h of transfection. Besides, the cell proliferation reagent CCK-8 (GeneView, America) was applied to approximately measure the cell viability. After plating the cells in the 96-well microtiter plates (Coring, NY, USA), 10 µl of CCK-8 reagent was added to each well and the cells were incubated for 2 h. Then, the value of optical density (OD) was acquired at 450 nm to determine the cell viability. Following the protocol, we further conducted the EdU assay through a Cell-Light EdU Cell Proliferation Kit (RiboBio, China). After transfection for 48 h, the transfected cells were then incubated with 50 mM EdU for another 3 h. Subsequently, 4% paraformaldehyde and Apollo Dye Solution were respectively utilized to fix the cells and stain the proliferating cells, along with Hoechst 33342 to mount the cells. After labeling, we photographed and enumerated the EdU-positive cells *via* the microscope under five indiscriminately selected visions. As for cell cycle detection, the transfected cells were fixed with 70% ethanol at 4°C for 24 h. Then, the cells were washed with PBS and stained with propidium oxide (PI) for 20 min in a dark room. Finally, the cell cycle was detected by flow cytometry, and the proportion of cells in the phase of G0/G1, S, or G2/M phases was compared. Transwell assay was mainly performed to measure the migration capacity of cells. Firstly, the concentration of transfected cells was adjusted to 2 × 10^5^ cells/ml. Secondly, 150 μl of cell suspension was added in the upper chamber, and 800 μl of RPMI-1640 medium involving 20% FBS in the lower chamber (the bottom of the 24-well plate). After incubating for 24 h under conditions of 37°C with 5% CO_2_, cotton swabs were then used to remove the upper layer cells of the membrane. The upper chamber was further fixed and stained respectively by methanol and crystal violet for 15 and 30 min, and then washed with PBS. Five visual fields were randomly chosen to photograph and enumerate the number of migrated cells under a microscope.

### IHC Staining and Immunofluorescence

IHC staining was employed to investigate the expression of APOBEC3A protein in formalin-fixed, paraffin-embedded tissue sections as described previously. Primary antibody against APOBEC3A (#AP20219a, abcepta) was used overnight at 4°C. Slides were then incubated for another 1 h using secondary antibody (#PK-8501, Vector Lab, USA). The complex was detected using Rabbit IgG mini-PLUS Kit visualized with DAB complex (#PK-8501, Vector Lab, USA). Besides, hematoxylin was applied to counterstain the nuclei. Sections were visualized under a microscope (10× or 40×). For immunofluorescence, the transfected cells were plated onto coverslips for 24 h and fixed for 20 min with 4% paraformaldehyde. After permeabilization for 30 min with 0.5% Triton X-100, cells were incubated in blocking buffer. Primary antibody against H2Ax (1:500, #ab229914, abcam, US) was utilized overnight at 4°C, then washed three times with PBS for 3 min. Alexa-488 goat anti-rabbit (1:500, #GB25303, Servicebio, China) was used as secondary antibody for 60 min and stained with 4’,6-diamidino-2-phenylindole (DAPI) for 15 min at room temperature. Finally, cells were regarded as positive if at least one focus was visible using a 40× objective in a confocal microscope.

## Results

### The Flow Chart of This Study

This study was systematically conducted through the following analytical processes (**Figure 1**). Firstly, we acquired the transcriptome information, somatic mutation profiles, and clinical data of 380 OC patients from the TCGA database, through which data immune-related DEGs and genomic-instability DEGs were recognized using the difference analysis. Secondly, APOBEC3A was further determined *via* intersection analysis of those above DEGs. Thirdly, three aspects of APOBEC3A were comprehensively analyzed, including immunity analysis, mutation analysis, and experimental verification. Among them, immunity analysis contained the correlation of APOBEC3A expression with TICs, ICPs, and IPS. Somatic analysis was about the correlation of APOBEC3A with TMB, somatic mutation, DDR genes, and m6A regulators. As for experimental verification, we have conducted the IHC, qPCR, FCM, Edu staining, and immunofluorescence to confirm the bioinformatical results of APOBEC3A.

### Identification of Immune-Related DEGs and Genomic Instability-Related DEGs in OC

**Figure 2:**
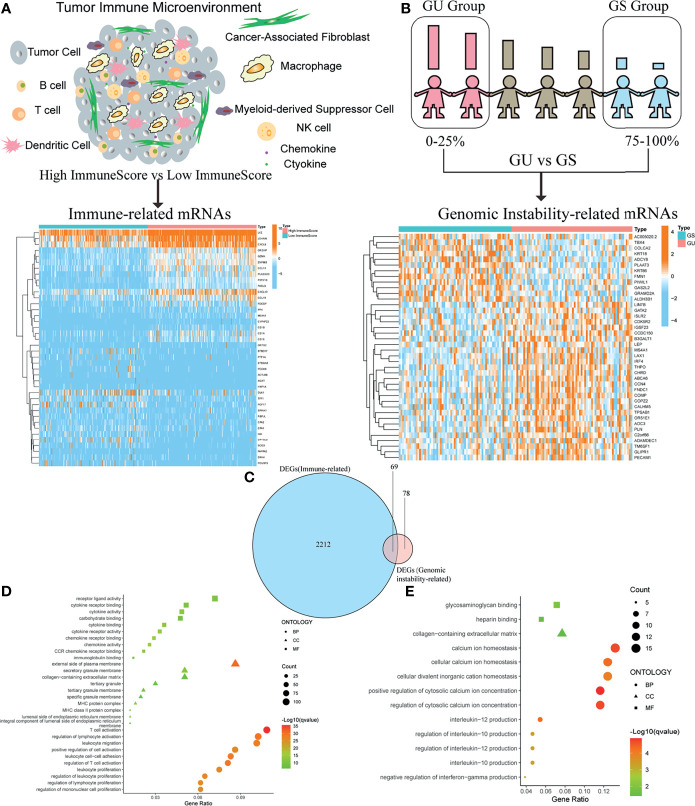
Identification of differentially expressed genes (DEGs) both correlated with tumor immune microenvironment (TIME) and genomic instability of ovarian cancer (OC). **(A)** Heatmap for immune-related DEGs generated by comparison of the high score group vs. the low score group in ImmuneScore. The row name of heatmap is the gene name, and the column name is the ID of samples that are not shown in the plot. Differentially expressed genes were determined by Wilcoxon rank sum test with *q* < 0.05 and fold-change > 0.58 after log2transformation as the significance threshold. **(B)** Heatmap for genomic instability-related DEGs by comparison of the genomic unstable (GU) group vs. the genomic stable (GS) group, similar to **(A)**. **(C)** Venn plot showing common DEGs shared by immune-related DEGs and genomic instability-related DEGs, and *q* < 0.05 and fold-change > 0.58 after log2transformation as the DEGs significance filtering threshold. **(D)** GO enrichment analysis for 2,281 immune-related DEGs; terms with *p* and *q* < 0.05 were believed to be enriched significantly. **(E)** GO enrichment analysis for 147 genomic instability-related DEGs; terms with *p* and *q* < 0.05 were believed to be enriched significantly.

In order to acquire immune-related genes in TME of OC, gene expression profiles were analyzed by means of comparing high- with low-ImmuneScore cohorts. Then, a total of 2,281 immune-related DEGs were identified from the median value of ImmuneScore (**Figure 2A** and **Table S2**). Similarly, we calculated the accumulated number of each OC sample’s somatic mutations and sorted the number according to the descending order. Then, OC samples whose cumulative number ranked the top 25% (*n* = 65) were divided into the GU cohort, and the last 25% (*n* = 70) into the GS cohort. Additionally, 147 genomic instability-related DEGs were extracted from comparing gene expression profiles of GU patients with GS patients (**Figure 2B** and **Table S3**). The above DEGs were all identified by the significance criteria with absolute value of log2 FC > 0.58 and FDR < 0.05. Since cumulative evidence has demonstrated that genomic instability could activate immunological recognition, launch immune responses, and facilitate the dynamic change of TIME, a gene intersection analysis of immune-related DEGs and genomic instability-related DEGs was further performed to see whether there existed any common genes. Interestingly, a total of 69 mutual DEGs were acquired through the intersection analysis (**Figure 2C**). That is, genome instability and immune microenvironment of OC are indeed closely linked at the level of gene expression. Moreover, functional enrichment analysis revealed that the immune-related DEGs were definitely associated with the immunological processes, like T-cell activation and lymphocyte activation regulation (**Figure 2D**). Simultaneously, a similar analytical method was utilized to assess the functional enrichment of genomic instability-related DEGs, indicating that these genes were basically enriched in the following biological processes such as calcium ion homeostasis and interleukin-12 (IL-12) production (**Figure 2E**). These results suggested that the long-lasting genomic instability of patients with OC may cause changes in tumor cell phenotypes, activate immune surveillance, and dynamically change the TME of OC.

### APOBEC3A Was Identified as a Protective Signature in OC Patients

**Figure 3:**
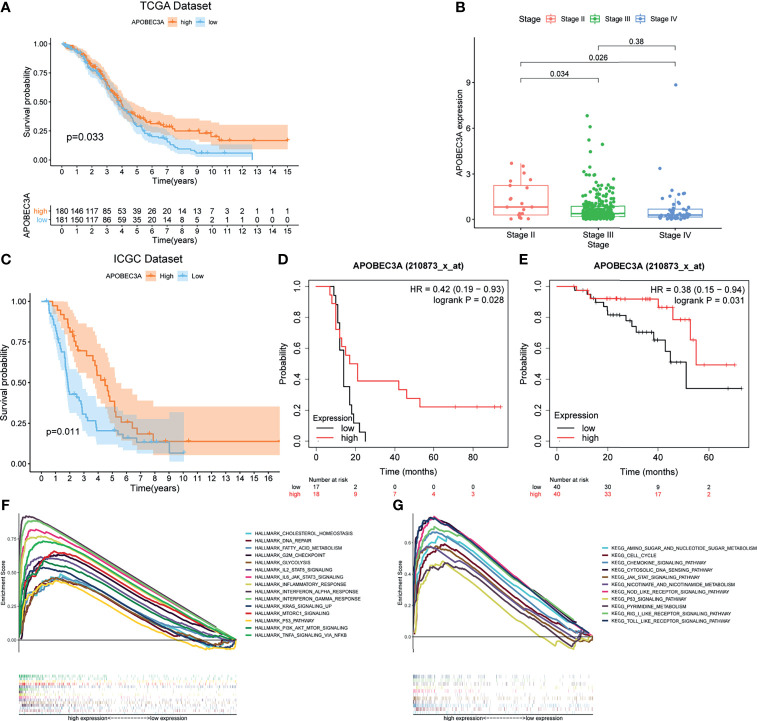
Correlation analysis of APOBEC3A expression with survival and clinicopathological staging characteristics of ovarian cancer (OC) patients. **(A)** Survival analysis for OC patients with different APOBEC3A expression. Patients were marked with high expression or low expression depending on comparing with the median expression level. *p* = 0.033 by log-rank test. **(B)** The correlation of APOBEC3A expression with clinicopathological stage characteristics. Kruskal–Wallis rank sum test acted as the statistical significance test. **(C)** The correlation of APOBEC3A expression with survival time from the International Cancer Genome Consortium (ICGC) database in OC patients, by log-rank test. **(D, E)** The correlation of APOBEC3A expression with survival time using the Kaplan–Meier plotter. **(F, G)** Enriched gene sets in the HALLMARK and KEGG gene sets, by samples of high APOBEC3A expression. Each line is represented by one particular gene set with unique color, and upregulated genes are located on the left, which approach the origin of the coordinates; by contrast, the downregulated ones lay on the right of the *x*-axis. Only gene sets with both NOM *p* < 0.05 and FDR *q* < 0.05 were considered significant. Only several top gene sets are shown in the plot.

After identifying the above 69 genes that were both linked to immune microenvironment and genomic instability of OC patients, we then equally separated tumor cases into different expression cohorts depending on the median level of gene expression, respectively. In addition, Kaplan–Meier analysis was further conducted by comparing high- and low-expression cohorts, demonstrating that there were 10 genes correlated with OS (APOBEC3A, C16orf54, CCL19, HTRA4, IRF4, LILRA5, MS4A1, NUGGC, PDCD1LG2, and SLAMF7). After that, the correlation analysis of these 10 genes’ expression with clinicopathological characteristics was performed, revealing that only APOBEC3A expression was significantly associated with stage classification (**Supplementary Figure 1**). As for APOBEC3A, OC patients with high expression were found to possess a longer survival time than low expression (**Figure 3A**, *p* = 0.033, by log-rank test). Moreover, APOBEC3A expression was descending along with the advanced stage classification of OC progression (**Figure 3B**). In order to verify APOBEC3A as a protective signature, we then used the OC samples from ICGC database and Kaplan–Meier plotter online database to further assess its prognostic value. The results from the ICGC database illustrated that APOBEC3A upregulation would have an improved OS (**Figure 3C**). In addition, online database analysis also showed that OC patients in high APOBEC3A cohort had a better OS than the low one, which was in accordance with the above results of this study (**Figures 3D, E**). These results exhibited that APOBEC3A could serve as a biomarker to forecast the survival and stage classification of OC patients. Moreover, GSEA focusing on HALLMARK gene sets and KEGG gene sets was employed for different APOBEC3A expression groups (**Table S4**). On the one hand, for HALLMARK gene sets, the APOBEC3A high-expression cohort genes were basically enriched not only in immune biological processes but also in genomic instability-related pathways, taking the IL2–STAT5 signaling pathway, inflammatory response, DNA repair, and G2M checkpoint for example (**Figure 3F**). On the other hand, similarly, APOBEC3A high-expression cohort genes were also intimately related to immunological functions and genomic instability-associated processes, such as chemokine signaling pathway, JAK-STAT signaling pathway, cell cycle, and cytosolic DNA sensing pathway (**Figure 3G**). Synchronously, there exhibited no enriched pathways for the genes in the APOBEC3A low-expression cohort. The above results illustrated that APOBEC3A expression was indeed strongly correlated with the immune microenvironment and genome instability of OC and could act as a protective biomarker to predict the prognosis of OC patients.

### Immunity Analysis Between APOBEC3A High- and Low-Expression Cohorts

**Figure 4:**
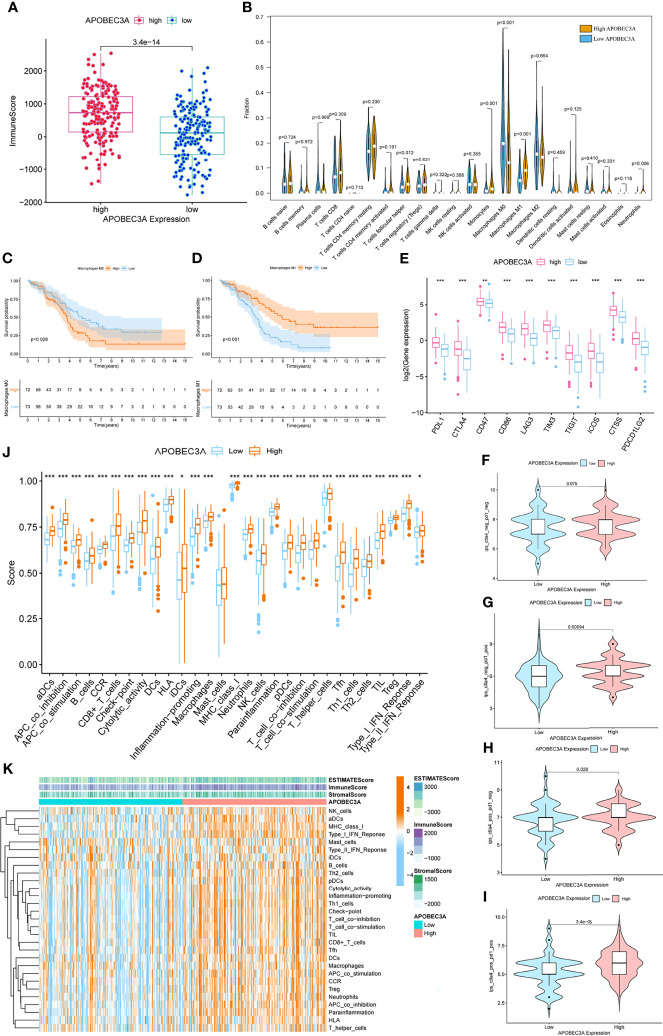
Immunity analysis of APOBEC3A high- and low-expression cohorts in ovarian cancer (OC) patients. **(A)** The correlation of APOBEC3A with ImmuneScore, Wilcoxon rank sum was applied for the significance test. **(B)** Violin plot showed the ratio differentiation of 22 types of immune cells between OC tumor samples with high or low expression relative to the median of APOBEC3A expression, and Wilcoxon rank sum was applied for the significance test. **(C, D)** Kaplan–Meier curves show the independent relevance between overall survival time and Macrophage M0 and Macrophage M1. **(E)** The correlation between common inhibitory immune checkpoints (ICPs) and APOBEC3A high- and low-expression cohorts; Wilcoxon rank sum was applied for the significance test. The results showed that the expression of ICPs was all significantly positive-correlated with APOBEC3A group. ***p < 0.001. **(F)** The correlation of APOBEC3A with Immunophenoscores (IPS) in OC patients who have not received anti-CTLA4 or anti-PD-1 immunotherapy. **(G)** The correlation of APOBEC3A with IPS in OC patients who have only received anti-PD-1 immunotherapy. **(H)** The correlation of APOBEC3A with IPS in OC patients who have only received anti-CTLA4 immunotherapy. **(I)** The correlation of APOBEC3A with IPS in OC patients who have received anti-CTLA4 combined with anti-PD-1 immunotherapy. **(J)** ssGSEA for the association between immune cell subpopulations and related functions. ***p < 0.001, **p < 0.01, *p < 0.05. **(K)** Heatmap for immune responses based on ssGSEA among APOBEC3A high- and low-expression group.

**Figure 5:**
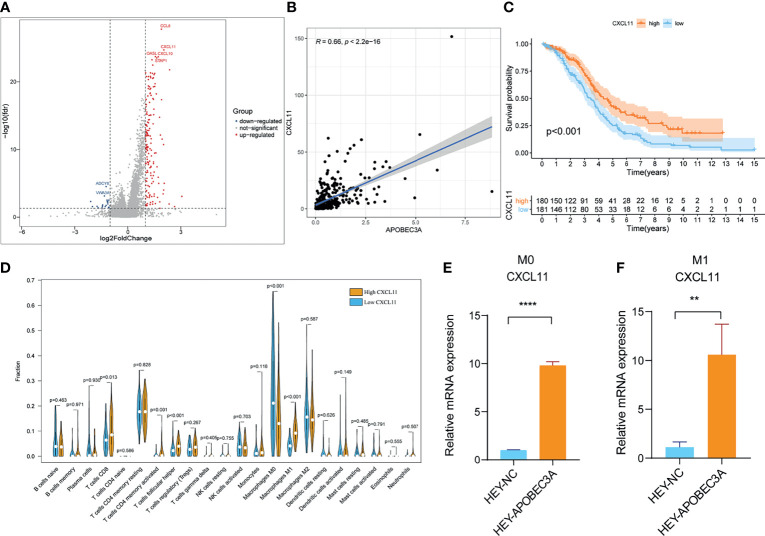
Analyses of APOBEC3A high- and low-expression cohorts in ovarian cancer (OC) patients. **(A)** Volcano plot for differentially expressed genes (DEGs). The blue and red dots represented the significantly downregulated and upregulated genes, respectively, and the gray dots represented the genes without differential expression. FDR < 0.05,|log2 FC|> 1, and *p* < 0.05. **(B)** The correlation analysis of APOBEC3A expression with CXCL11 expression. **(C)** Kaplan–Meier curve shows the independent relevance between overall survival time and CXCL11 expression. **(D)** Violin plot showed the ratio differentiation of 22 types of immune cells between OC tumor samples with high or low expression relative to the median of CXCL11 expression, and Wilcoxon rank sum was applied for the significance test. **(E, F)** RT-qPCR showed that APOBEC3A was positively correlated with CXCL11 in Macrophage M0 **(E)** and M1 **(F)**. All **p < 0.01, ****p < 0.0001; the data represent the mean ± SD from triplicate measurements.

Since APOBEC3A was found to be intimately associated with immune-related biological functions in this study, we conducted a series of comprehensive bioinformatical analyses to further acknowledge how APOBEC3A expression influences immune microenvironment of OC. Firstly, we analyzed the relationship between APOBEC3A expression with ImmuneScore, and the results demonstrated that OC patients in a higher level of the APOBEC3A group exhibited a higher ImmuneScore (**Figure 4A**, *p* = 3.4e-14). Secondly, the CIBERSORT method was then performed to investigate the relationship between APOBEC3A and specific immune cells, which calculated the proportion of 22 different tumor-infiltrating immune subtypes. A total of five types of TICs were considered as significantly related to APOBEC3A expression, including Macrophage M0, Macrophage M1, Monocytes, T-cell follicular helper, and Neutrophils (**Figure 4B**). Among them, it is worth noting that APOBEC3A expression was positively correlated with the proportion of Macrophage M1 (*p* < 0.001) and negatively correlated with M0 (*p* < 0.001). We further analyzed the correlation of immune cells proportion with survival time, illustrating that OC patients with a lower proportion of Macrophage M0 or a higher proportion of Macrophage M1 would have a longer OS (**Figures 4C, D**, *p* = 0.028, *p* < 0.001, respectively). These results may explain the better survival of the high APOBEC3A expression group. Thirdly, in order to assess the prognostic value of APOBEC3A expression to predict the effect of immunotherapy response, the association between gene expression and ICPs was conducted. The analytical results illustrated that APOBEC3A expression exhibited a positive correlation with ICPs such as PDL1, CTLA4, LAG3, and TIGIT (**Figure 4E**). Besides, the analytical results of IPS with APOBEC3A expression have demonstrated that patients in the high APOBEC3A expression group could have a higher IPS of anti-PD1 and anti-CTLA4 therapy (**Figures 4F–I**), which means a better immunotherapy response. These results have fully illustrated that APOBEC3A could be a favorable signature for effectively forecasting the effect of immunotherapy in OC patients. In addition, we applied ssGSEA to further conduct the correlation analysis on APOBEC3A with immune-related functions and immune cell types, showing that many immunological processes were significantly different in different APOBEC3A expression cohorts (**Figures 4J, K**). In order to further demonstrate the role of APOBEC3A in influencing immune microenvironment of OC, we separated cancer patients into two different APOBEC3A expression cohorts according to the median level and finally identified 187 DEGs by comparing these two groups (**Figure 5A**). In these DEGs, C-X-C motif chemokine 11 (CXCL11) was then selected for the further analysis since its expression changed in a remarkably significant level and its correlation with APOBEC3A was strong (**Figure 5B**, *R* = 0.66, *p* < 2.2e-16). In addition, the survival analysis showed that high CXCL11 expression had a longer OS (**Figure 5C**, *p* < 0.001, by log-rank test). Similar to the results of APOBEC3A, CXCL11 expression was also found to be positively related to the proportion of Macrophage M1, and M0, the opposite (**Figure 5D**, *p* < 0.001). Moreover, we performed RT-qPCR to further confirm the correlation of APOBEC3A with CXCL11, and the results indicated that upregulation of APOBEC3A expression in HEY cells increased the CXCL11 expression in M0 and M1 macrophages (**Figures 5E, F**, *p* < 0.0001 and *p* < 0.01, respectively). The above results illustrated that APOBEC3A might increase the secretion level of CXCL11 in M0 and M1 macrophages, and thus a better survival in OC patients.

### APOBEC3A in the Role of Anti-PD-L1 Immunotherapy

**Figure 6:**
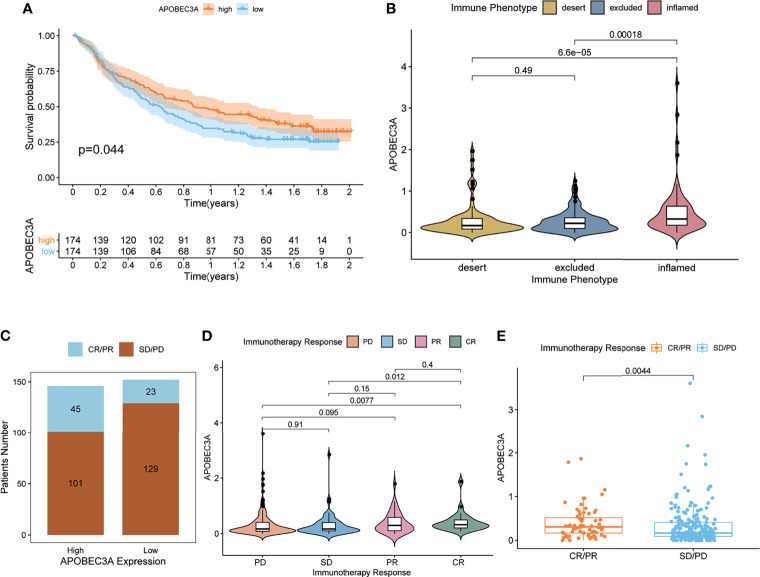
APOBEC3A in the role of anti-PD-L1 immunotherapy. **(A)** Survival analyses for high (174 cases) and low (174 cases) APOBEC3A expression patient groups in the anti-PD-L1 immunotherapy cohort using Kaplan–Meier curves (IMvigor210 cohort; *p* = 0.044, Log-rank test). **(B)** Differences in APOBEC3A among distinct tumor immune phenotypes in the IMvigor210 cohort. The lines in the boxes represented median value (by Kruskal–Wallis test). **(C)** The number of patients with response to PD-L1 blockade immunotherapy in high or low APOBEC3A expression groups. SD, stable disease; PD, progressive disease; CR, complete response; PR, partial response. Responser/Nonresponer: 45/101 in the high APOBEC3A expression groups and 23/129 in the low APOBEC3A expression groups. **(D)** Distribution of APOBEC3A in distinct anti-PD-L1 clinical response groups. **(E)** The correlation of APOBEC3A with immunotherapy response, Wilcoxon rank sum was applied for the significance test.

More critically, we ultimately selected an immunotherapeutic dataset of urothelial cancer called IMvigor210 cohort from Mariathasan et al. (32) to further demonstrate the prognostic value of using APOBEC3A as a biomarker for ICB therapy such as anti-PD-L1 immunotherapy. From the results, we could clearly see that APOBEC3A upregulation exhibited a significantly improved survival that also provided good proofs for our previous results (**Figure 6A**, *p* = 0.044, by log-rank test). Besides, the correlation of different APOBEC3A expression with different immune phenotypes (desert, excluded, inflamed) was also explored since patients with inflamed immune phenotype were more likely to benefit from immunotherapy, while the other two types exhibited the opposite. The results demonstrated that higher APOBEC3A expression was found to be strongly associated with inflamed immune phenotype while the lower one was found to be associated with the excluded and desert types, which meant that it is more difficult for patients with APOBEC3A downregulation to obtain benefits from immunotherapy (**Figure 6B**). In addition, we further investigated the therapeutic responsiveness and clinical benefits to anti-PD-L1 immunotherapy by comparing different APOBEC3A expression cohorts, and cancer patients with high expression of APOBEC3A were found to have a more positive response to immunotherapy, thus a better clinical outcome (**Figures 6C–E**). The above results strongly indicated that APOBEC3A could represent the immune status of TME, which might play an essential part in forecasting the response to immunotherapy as well.

### Mutation Analysis Between APOBEC3A High- and Low-Expression Cohorts

**Figure 7:**
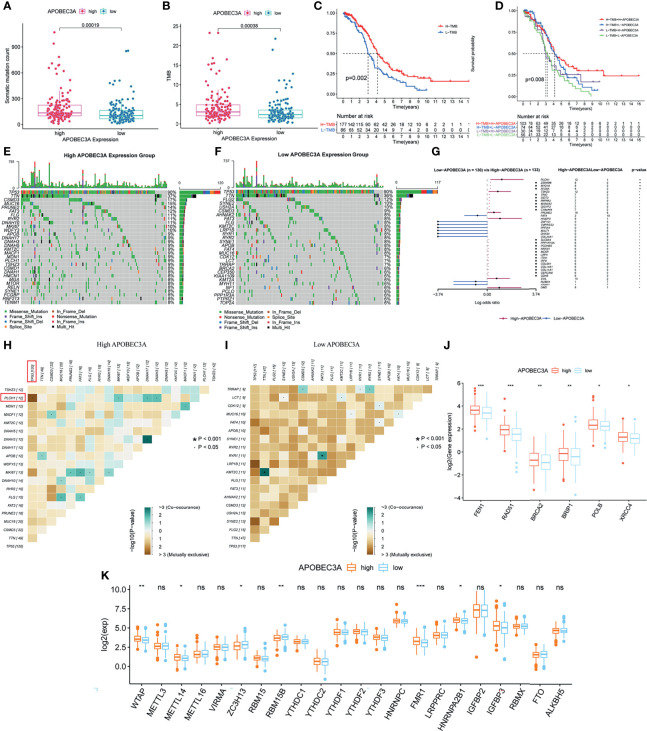
Mutation analysis of APOBEC3A high- and low-expression cohorts in ovarian cancer (OC) patients. **(A)** The correlation of APOBEC3A with somatic mutation count, Wilcoxon rank sum was applied for the significance test. **(B)** The correlation of APOBEC3A with tumor mutation burden (TMB). **(C)** Survival analysis for OC patients with high- and low-TMB groups. Patients were marked with high TMB or low TMB depending on comparing with the median TMB level. *p* = 0.002 by log-rank test. **(D)** Kaplan–Meier curve analysis of overall survival is shown for patients classified according to TMB and APOBEC3A expression. Statistical analysis was performed using the log-rank test. **(E, F)** Waterfall plot shows the mutation distribution of the top 30 most frequently mutated genes. The central panel shows the types of mutations in each OC sample. The upper panel shows the mutation frequency of each OC sample. The bar plots on the left and right side show the frequency and mutation type of genes mutated in the low-APOBEC3A **(E)** and high-APOBEC3A **(F)** cohorts, respectively. The bottom panel is the legend for mutation types. **(G)** Forest plot and waterfall plot display the significantly differentially mutated genes between APOBEC3A high- and low-expression cohorts. **p < 0.01, *p < 0.05. **(H, I)** The heatmap illustrates the mutually co-occurring and exclusive mutations of the top 20 frequently mutated genes. The color and symbol in each cell represent the statistical significance of the exclusivity or co-occurrence for each pair of genes. TP53 and PLCH1 are marked out in the red rectangle. **(J)** The correlation of APOBEC3A with DNA damage response (DDR) genes. ***p < 0.001, **p < 0.01, *p < 0.05. **(K)** The correlation of APOBEC3A with m6A regulators. ***p < 0.001, **p < 0.01, *p < 0.05, ns, not significant.

Apart from the immune-related functions, APOBEC3A was also identified as a genomic instability-related gene. Therefore, a variety of integrative analyses on genomic instability-related processes were performed further. Firstly, we analyzed the association between APOBEC3A expression and somatic mutation count, showing that patients with high APOBEC3A expression had more somatic mutation (**Figure 7A**, *p* = 0.00019). Secondly, correlation analysis of APOBEC3A expression with TMB was also conducted, whose results have demonstrated that the expression of APOBEC3A correlated positively with TMB in OC patients (**Figure 7B**, *p* = 0.00038). As many published literatures reported, TMB could serve as a biomarker to predict immunotherapy response (45). The above results further supported the idea that APOBEC3A could be a possible signature to acknowledge the effect of immunotherapy. Besides, survival analysis has indicated that patients with high TMB would possess a longer OS (**Figure 7C**, *p* = 0.002). When TMB combined with APOBEC3A expression, we could clearly see that high TMB with high APOBEC3A expression had the best prognosis, and contrarily, low TMB with low APOBEC3A expression had the worst (**Figure 7D**, *p* = 0.008). Thirdly, on account that APOBEC3A seemed to play an essential role in genomic instability of OC, we further wondered whether there would exist some differentially mutated genes by comparing APOBEC3A high- and low-expression cohorts. Naturally, such difference analysis focusing on somatic mutation data was applied then and there exactly existed some difference in these two cohorts. Interestingly, the mutation of TP53 and TTN ranked the first and the second in both cohorts (**Figures 7E, F**). Thirty-seven differentially mutated genes were acquired (**Table S5**), among which Phospholipase C Eta 1 (PLCH1) with high APOBEC3A expression mutated significantly more than the low one (**Figure 7G**). Besides, we analyzed the co-occurring and exclusive mutations of these two expression cohorts, indicating that PLCH1 and TP53 exhibited an exclusive mutation in the high APOBEC3A expression group (**Figures 7H, I**). The results meant that PLCH1 and TP53 might play a similar part in the same pathway with high APOBEC3A expression. TP53 mutation was reported to be connected with poor prognosis and metastasis of OC patients (46, 47). That is, patients with high APOBEC3A expression seemed to have more PLCH1 mutation and relatively less TP53 mutation, which was consistent with previous results that APOBEC3A was a protective factor in OC. Fourthly, correlation analysis of DDR genes and APOBEC3A was then performed since DDR was reported to be closely related to genomic instability. As expected, DDR genes like RAD51, FEN1, and BRCA2 were significantly positive-associated with APOBEC3A (**Figure 7J**). Last but not the least, APOBEC3A was also found to be significantly correlated with m6A regulators such as WTAP, METTL14, ZC3H13, RBM15B, and FMR1 (**Figure 7K**). Emerging evidence has suggested that the expression of m6A regulators was critical for carcinogenesis and cancer development (48), which strengthened the important position of APOBEC3A in OC. The comprehensive analyses confirmed the intimate relationship of APOBEC3A with genomic instability, demonstrating that APOBEC3A had the potential to be a helpful biomarker in OC.

### Experimental Verification of APOBEC3A in OC

#### APOBEC3A Expression Was Decreased in OC

**Figure 8:**
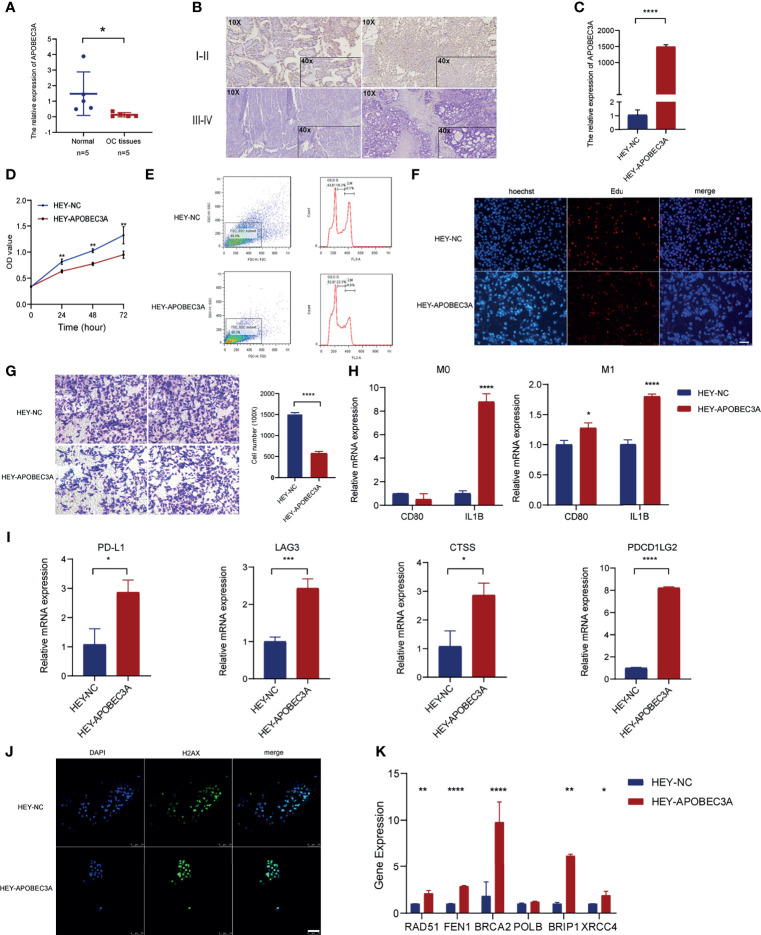
Functional verification of APOBEC3A in ovarian cancer (OC) cells. **(A)** The mRNA level of APOBEC3A in five OC tissues and five normal ovarian tissues. **(B)** The expression of APOBEC3A protein in I–II stage and III–IV stage OC tissues was detected by IHC (magnification ×100 and ×400). **(C)** The transfection efficiency of APOBEC3A was verified in HEY cells by RT-qPCR. **(D)** APOBEC3A-reduced proliferation rate of HEY cells by CCK-8. **(E)** Flow cytometry analysis showed overexpression of APOBEC3A enhanced HEY cell cycle arrest. APOBEC3A increased the percentage in the G0/G2 phase compared with HEY-NC cells. **(F)** EdU assay showed that the proliferative ability of HEY cells was inhibited by APOBEC3A. Nuclei are shown in blue (Hoechst) (magnification ×100) **(G)** Transwell assay showed cell migration ability of HEY cells was inhibited by APOBEC3A compared with HEY-NC cells. Bar equals 100 µm. **(H)** Co-culture assay showed APOBEC3A overexpressed in HEY promoted the expression of M1 markers (CD80 and IL1B) in macrophages (M0 and M1). **(I)** RT-qPCR showed that APOBEC3A was positively correlated with PD-L1, LAG3, CTSS, and PDCD1LG2. **(J)** H2Ax immunofluorescence showed that DNA damage foci formation was increased in HEY-APOBEC3A cells. Nuclei are shown in blue (DAPI). Green: H2Ax foci. Bar equals 75 µm. **(K)** RT-qPCR showed that APOBEC3A was positively correlated with RAD51, FEN1, BRCA2, BRIP1, and XRCC4. All *p < 0.05, **p < 0.01, ***p < 0.001, ****p < 0.0001; the data represent the mean ± SD from triplicate measurements.

Through the bioinformatic analysis, it could be apparently seen that APOBEC3A expression was gradually declining along with the advanced stage of OC. To further validate the results, we detected APOBEC3A expression in five specimens of OC and five normal ovarian tissues by RT-qPCR. The results demonstrated that the expression of APOBEC3A was even lower in OC tissues compared with normal ones (*p* < 0.05) (**Figure 8A**). We also assessed the protein expression of APOBEC3A in paraffin-embedded tissues. In accordance with the prediction, lower expression of APOBEC3A was associated with advanced stage classification (**Figure 8B**).

#### APOBEC3A Inhibited OC Cell Viability, Proliferation, and Migration

After transfection with APOBEC3A-pcDNA3.1 and pcDNA3.1 (empty vector) in HEY cells, the overexpression efficiency was confirmed by RT-qPCR (**Figure 8C**). APOBEC3A expression in the HEY-APOBEC3A group was higher than the HEY-NC group. Next, a series of functional assays were performed. CCK-8 assay results showed that overexpression of APOBEC3A inhibited HEY cell viability (**Figure 8D**). The known effect of APOBEC3A is leading to DNA double-strand breaks and DNA damage, thereby disrupting cell cycle of tumor cells. Therefore, we detect the cell cycle phase of HEY after transfection of APOBEC3A. As shown in **Figure 8E**, upregulation of APOBEC3A expression in HEY cells increased the proportion of cells in the G0/G1 phase and decreased the proportion of cells in the S and G2/M phase, indicating that APOBEC3A induced OC cell arrest and blocked mitosis. Consistent with the result of the CCK-8 assay, EdU assay confirmed that EdU staining was decreased in the HEY-APOBEC3A group (**Figure 8F**). Furthermore, transwell assay results indicated that overexpression of APOBEC3A could weaken the migration ability of HEY cells (**Figure 8G**).

#### APOBEC3A Promoted the Polarization of M1 Macrophages and Predicted the Immunotherapy Response

Knowing that APOBEC3A expression was positively correlated with the proportion of M1 macrophages, we investigated the impact of APOBEC3A on polarization of M1 macrophages. We found that IL1B expression of M0 macrophages was increased after co-culture of macrophages and HEY cells transfected with APOBEC3A overexpression plasmid (**Figure 8H**). Furthermore, the expression of IL1B and CD80, which are the markers of M1 macrophages, were significantly increased in M1 macrophages after coculturing M1 macrophages and HEY-APOBEC3A compared with control cells (**Figure 8H**). These results indicated that APOBEC3A could promote the polarization of M1 macrophages. At the same time, we verified the important role of APOBEC3A in predicting the effect of immunotherapy response. As illustrated in **Figure 8I**, the expression levels of these ICPs such as PDL1, LAG3, CTSS, and PDCD1LG2, with the overexpression of APOBEC3A, were increased significantly, indicating that APOBEC3A could predict a better response of immunotherapy in OC patients.

#### APOBEC3A Contributed to DNA Damage Foci Formation in OC Cells

Anti-H2Ax immunofluorescence could detect DNA damage response. So, we performed the IF assay using anti-H2Ax staining to detect the frequency of DNA damage foci under the effect of APOBEC3A. The result showed that upregulation of APOBEC3A expression in HEY increased the DNA damage foci. As shown in **Figure 8J**, HEY cells that are transfected with APOBEC3A were almost stained by the dye of anti-H2Ax antibody, suggesting that APOBEC3A could contribute to the DNA damage in tumor cells thereby playing a role of depressing tumor development.

#### The Correlation of APOBEC3A With DNA Repair Response Genes

Somatic mutation in the cancer genome is the result of continuous accumulation of mutations. Double-strand break (DSB) is the most disastrous form of DNA damage that causes genome instability. The correlation analysis predicted the association of APOBEC3A with DDR genes, and we performed RT-qPCR assay to further verify this point. The results indicated that APOBEC3A expression was positively associated with DDR genes such as RAD51 (*p* < 0.01), FEN1 (*p* < 0.0001), BRCA2 (*p* < 0.0001), BRIP1 (*p* < 0.01), and XRCC4 (*p* < 0.05) (**Figure 8K**).

## Discussion

Currently, emerging data have reported that ICB immunotherapy could be hopeful for OC patients with recurrence (49). A variety of previously published literatures have reported that OC patients who met the following criteria could probably benefit more from immunotherapy using anti-PD-1/PD-L1 antibodies: (i) highly expressed in PD-L1, (ii) high microsatellite instability (MSI-H), (iii) defective mismatch DNA repair (dMMR), (iv) high tumor-infiltrating lymphocytes with high TMB, and (v) T-cell inflammatory gene expression profile (GEP) (50–52). Disappointingly, the overall response rate of immunotherapy was still unsatisfactory since the TME of OC was generally in a state of immunosuppression (22). That is, the number of activated TICs and the capacity of them to kill tumor cells were all inhibited in such immunosuppressive microenvironment. Therefore, it is worthy of our consideration to distinguish OC patients who can benefit most from ICB immunotherapy. It is also urgently needed to explore a prognostic biomarker to change the immune microenvironment of OC from “cold” to “hot”. In this study, we tried to determine a promising biomarker for efficiently forecasting the effect of immunotherapy and prognosis of OC patients.

Apart from the impact of immune component, genomic instability would also play a crucial part in influencing immunotherapy response (53). Genomic instability was acknowledged as one of the hallmarks of cancer and could be frequently observed in a variety of malignant tumors, which was found to be closely related to tumor resistance and progression (23, 53). Additionally, multiple studies have made enormous efforts to discover the relationship between genomic instability and immunological processes, ultimately demonstrating that neoantigens could serve as an intermediary bridge for tumor-specific somatic mutations to induce the activation of T lymphocytes (53, 54). Nevertheless, systematic analyses on gene expression profiles targeting genomic instability and immune microenvironment of OC are still in its infancy and far from satisfactory. This study is an important practice to combine tumor immune microenvironment with the genomic instability in OC. Consequently, it is of great importance and clinical value to ascertain a powerful biomarker for predicting the immunotherapy response and overall prognosis of OC patients on the basis of genomic instability and immune microenvironment.

In this article, APOBEC3A was finally identified based on the gene intersection analysis of immune microenvironment and genomic instability. APOBEC3A belongs to APOBEC3 family and is located on the chromosomal 22q13 (55). Plenty of researches have illustrated that APOBEC3 family member expression was intently related to the survival of cancer patients (56). For example, APOBEC3B was found to express higher in patients with improved survival in clear cell ovarian cancer (CCOC), which was also expected to be a promising biomarker for forecasting the effect to platinum-based therapy (57–59). Besides, high APOBEC3G expression was also found to be associated with an improved progression-free survival, a prolonged OS, and a larger amount of T-cell infiltration in OC (60). However, the correlation of APOBEC3A expression with survival of OC patients has not been confirmed until now. In our study, APOBEC3A was discovered to be a protective prognostic biomarker in OC on account of the results that patients with high APOBEC3A expression had a longer survival time and its expression was declining with the advanced stage classification. Verification experiments using RT-qPCR and IHC were also conducted, demonstrating that lower expression of APOBEC3A was associated with advanced stage classification. Moreover, a series of functional assays were applied to further verify the association of APOBEC3A with viability, proliferation, and migration in HEY cells using CCK-8 assay, cell cycle detection, EdU staining, and transwell assay. The results illustrated that upregulation of APOBEC3A could weaken the viability, proliferation, migration of OC cells, confirming the important position of APOBEC3A as a protective factor in OC.

GSEA analysis has confirmed the hypothesis that APOBEC3A was intimately associated with immune-related biological pathways and genomic instability-related processes. From searching the existing studies, we found that APOBEC3B overexpression could act as a promising signature in predicting the ICB responsiveness of non-small cell lung cancer (NSCLC) (61). However, the relationship of APOBEC3A with immune microenvironment of OC has not been explored enough yet. According to the comprehensively integrative analysis, it could be easily concluded that APOBEC3A was able to act as an efficient biomarker for predicting the response of immunotherapy and representing the immune-dominant status of TME in OC patients. Increasing evidence has demonstrated the fact that many kinds of immune cells in TME could be crucial for tumor immunotherapy response, taking the CD8^+^ T lymphocytes, macrophages, and dendritic cells for instance. From the results of correlation analysis and ssGSEA, we could clearly see that OC patients with high APOBEC3A expression possessed a larger proportion of macrophage M1 and more activated immunological functions. Macrophage M1 is known to be remarkedly connected with anti-tumor effect and better prognosis, which means regulating the expression of APOBEC3A might be an available strategy to change the number of activated immune tumor-infiltrating cells, and thus improve the prognosis and sensitivity of immunotherapy in OC. In this study, CXCL11 was found and verified to be strongly positive-associated with APOBEC3A in OC patients, and upregulation of APOBEC3A could increase the secretion level of CXCL11 in M0 and M1 macrophages. Accumulating evidence and clinical trials have demonstrated that CXCL11 could be a biomarker to predict the survival and immunotherapy response of OC patients (62). Besides, IL1B and CD80 were detected to be significantly increased in the HEY cells with APOBEC3A upregulation, indicating that APOBEC3A could accelerate the transformation of M0 macrophages to M1 in tumor immune microenvironment of OC patients. That is to say, high expression of APOBEC3A might increase the secretion level of chemokine by M1 macrophages and promote the polarization of M1, thus an improved survival. Additionally, insufficient expression value of ICPs such as PD-L1, CTLA4, and TIGIT was one of the reasons causing low response rate of ICB therapy. Interestingly, APOBEC3A expression was discovered and confirmed to be positively related to the level of ICPs, illustrating that OC patients in the high APOBEC3A expression group would have a better response to ICB therapy. Moreover, IPS was acknowledged to be an evaluation index for assessing the response of ICB therapy, and higher IPS means better immunotherapy response. Similarly, correlation analysis of IPS with APOBEC3A confirmed the results that patients with high APOBEC3A expression would exhibit a relatively higher IPS after accepting ICB therapy targeting PD-L1 or CTLA4, and such patients could benefit more from immunotherapy. More importantly, further analyses on an immunotherapeutic cohort have demonstrated that higher expression of APOBEC3A was tightly related to the inflamed immune phenotype and a better clinical response to immunotherapy, which proved the predictive value of APOBEC3A on immunotherapy responsiveness. From the above comprehensive analyses concentrating on immune-related processes, we could come to the conclusion that APOBEC3A was a fantastic biomarker for improving the immunosuppressive microenvironment and foretelling the response to immunotherapy in OC patients.

As for the role in genomic instability, a series of analyses focusing on somatic mutation data were performed further by comparing APOBEC3A high- with low-expression cohorts. By carefully reviewing the existing literatures, we surprisedly realized the fact that the overexpression of APOBEC family genes is exactly the reason for unfavorable mutagenesis and genomic instability in cancers (63). Among them, APOBEC3A was known as a DNA/RNA editing enzyme catalytic polypeptide that could cause many undesirable passenger hotspot mutations in tumors (64–66). Nevertheless, there were few studies on exploring the association of APOBEC3A expression with somatic mutation in OC. In this article, APOBEC3A was initially found to be positively correlated with genomic instability, which meant more somatic mutation count would be detected in the high APOBEC3A expression group of OC patients. Additionally, cumulative somatic mutation could facilitate the production of tumor neoantigens and activate immunological processes, thus a better survival, which was consistent with the survival results we got before. Furthermore, accumulating reported studies have suggested TMB as a powerful biomarker to predict the response of immunotherapy (67). Besides, increasing clinical trials have confirmed the marvelous performance of TMB on predicting the efficacy of immunotherapy in many types of tumors such as melanoma and NSCLC (68–70). The results of correlation and survival analyses have demonstrated that OC patients with high expression of APOBEC3A would have a relatively high TMB and therefore a longer survival time. When TMB combined with APOBEC3A expression, there was still significant difference in survival. These results revealed that APOBEC3A could be a possible biomarker for predicting TMB and thus immunotherapy response of OC patients. Given that APOBEC3A was a mutation driver gene, investigating the differentially mutated genes in different APOBEC3A expression groups is of great importance. According to the results, we could obviously identify PLCH1 as the most differentially mutated gene. More importantly, PLCH1 was also found to be exclusively mutated with TP53, which illustrated that PLCH1 might have similar functions with TP53 in the same pathway. Additionally, the DDR pathway is acknowledged to maintain the genome stability when DNA damage occurs, which has been found to have a close correlation with response to ICB therapy (71–73). Also, APOBEC3A expression was reported to cause DNA damage responses of cancers (74, 75). In our study, APOBEC3A was actually confirmed to be associated with DDR genes such as RAD51, FEN1, and BRCA2. Furthermore, experimental verification using immunofluorescence and RT-qPCR confirmed the positive correlation of APOBEC3A with DNA damage and DDR genes. These results have verified the importance of APOBEC3A on genomic instability, indicating that APOBEC3A had the potential to serve as a biomarker to predict the immunotherapy response of OC.

Nowadays, immunotherapy has occupied an essential position for clinical therapeutic strategies of OC. Dismayingly, only a small percentage of patients have the opportunity to benefit from immunotherapy, which is an enormous challenge for scientists and doctors. Therefore, it is of vital importance to distinguish proper patients who have a better response to immunotherapy, which will be helpful to optimize personalized treatment strategies and rationally use medical resources. Compared to normal people, OC patients have unique characteristics in many aspects such as more immunosuppressive microenvironment and more unstable genomes, thus causing low response to immunotherapy to some extent. Purposefully, through the comprehensive analyses focusing on immune microenvironment and genomic instability of OC, a promising prognostic biomarker was ultimately identified and verified using basic experiments. That is, based on the transcriptome profiles and somatic mutation information, APOBEC3A was excitedly recognized and verified as a protective factor of OC, which could predict the prognoses of OC patients, especially for the response to immunotherapy. Equally important, this study has demonstrated that targeting the expression of APOBEC3A might be an advantageous strategy to transform the immune microenvironment from “cold” to “hot”, and therefore improve clinical treatment.

## Conclusion

In conclusion, APOBEC3A was identified as a protective factor from comprehensive analyses based on the immune microenvironment and genomic instability of OC. Additionally, APOBEC3A had the potential to serve as a promising prognostic biomarker for foretelling the survival and immunotherapy response of OC patients. The results shed new light on providing a powerful target for representing the immune-dominant status in TME and identifying OC patients who are effective in immunotherapy, which might accelerate the clinical application and improve treatment effect.

## Data Availability Statement

The original contributions presented in the study are included in the article/**Supplementary Material**. Further inquiries can be directed to the corresponding author.

## Ethics Statement

The studies involving human participants were reviewed and approved by the Medical Ethics Committee of Shanghai First Maternity and Infant Hospital. The patients/participants provided their written informed consent to participate in this study.

## Author Contributions

SX conceived and designed the manuscript. FX analyzed and interpreted the data. TL and ZZ performed the basic experiments. FX and TL wrote the manuscript. SX, ZZ, and CZ helped with manuscript and data review. All authors contributed to the article and approved the submitted version.

## Funding

This work was supported by the National Natural Science Foundation of China (grant no. 81772762), the Clinical Science and Technology Innovation Project of Shanghai Shenkang Hospital Development Center (grant no. SHDC12019X34), the Natural Science Foundation of Shanghai (grant no. 21ZR1450900), the Shanghai Science and Technology Planning Project (grant no. 21Y11907000), and the National Key R&D Program of China (Grant No. 2017YFA0104603).

## Conflict of Interest

The authors declare that the research was conducted in the absence of any commercial or financial relationships that could be construed as a potential conflict of interest.

## Publisher’s Note

All claims expressed in this article are solely those of the authors and do not necessarily represent those of their affiliated organizations, or those of the publisher, the editors and the reviewers. Any product that may be evaluated in this article, or claim that may be made by its manufacturer, is not guaranteed or endorsed by the publisher.

## References

[1] Sung H Ferlay J Siegel RL Laversanne M Soerjomataram I Jemal A. Global Cancer Statistics 2020: GLOBOCAN Estimates of Incidence and Mortality Worldwide for 36 Cancers in 185 Countries. CA Cancer J Clin (2021) 71(3):209–49. doi: 10.3322/caac.21660 33538338

[2] Allemani C Matsuda T Di Carlo V Harewood R Matz M Niksic M. Global Surveillance of Trends in Cancer Survival 2000-14 (CONCORD-3): Analysis of Individual Records for 37 513 025 Patients Diagnosed With One of 18 Cancers From 322 Population-Based Registries in 71 Countries. Lancet (2018) 391(10125):1023–75. doi: 10.1016/S0140-6736(17)33326-3 PMC5879496 29395269

[3] Reid BM Permuth JB Sellers TA. Epidemiology of Ovarian Cancer: A Review. Cancer Biol Med (2017) 14(1):9–32. doi: 10.20892/j.issn.2095-3941.2016.0084 28443200 PMC5365187

[4] Lheureux S Gourley C Vergote I Oza AM. Epithelial Ovarian Cancer. Lancet (2019) 393(10177):1240–53. doi: 10.1016/S0140-6736(18)32552-2 30910306

[5] Holmes D. Ovarian Cancer: Beyond Resistance. Nature (2015) 527(7579):S217. doi: 10.1038/527S217a 26605761

[6] Matsuo K Sheridan TB Mabuchi S Yoshino K Hasegawa K Studeman KD. Estrogen Receptor Expression and Increased Risk of Lymphovascular Space Invasion in High-Grade Serous Ovarian Carcinoma. Gynecol Oncol (2014) 133(3):473–9. doi: 10.1016/j.ygyno.2014.03.563 PMC4170217 24674832

[7] Pennington KP Walsh T Harrell MI Lee MK Pennil CC Rendi MH. Germline and Somatic Mutations in Homologous Recombination Genes Predict Platinum Response and Survival in Ovarian, Fallopian Tube, and Peritoneal Carcinomas. Clin Cancer Res (2014) 20(3):764–75. doi: 10.1158/1078-0432.CCR-13-2287 PMC3944197 24240112

[8] Lindzen M Ghosh S Noronha A Drago D Nataraj NB Leitner O. Targeting Autocrine Amphiregulin Robustly and Reproducibly Inhibits Ovarian Cancer in a Syngeneic Model: Roles for Wildtype P53. Oncogene (2021) 40(21):3665–79. doi: 10.1038/s41388-021-01784-8 PMC8154589 33941851

[9] Shanmughapriya S Senthilkumar G Vinodhini K Das BC Vasanthi N Natarajaseenivasan K. Viral and Bacterial Aetiologies of Epithelial Ovarian Cancer. Eur J Clin Microbiol Infect Dis (2012) 31(9):2311–7. doi: 10.1007/s10096-012-1570-5 22402815

[10] Kuroki L Guntupalli SR. Treatment of Epithelial Ovarian Cancer. BMJ (2020) 371:m3773. doi: 10.1136/bmj.m3773 33168565

[11] York A. Overcoming Hurdles in Cancer Immunotherapy. Nat Rev Cancer (2021) 21(4):214–5. doi: 10.1038/s41568-021-00343-3 33619387

[12] Hindson J. FMT for Immunotherapy-Refractory Melanoma. Nat Rev Gastroenterol Hepatol (2021) 18(2):82. doi: 10.1038/s41575-021-00413-9 33437017

[13] Rocconi RP Grosen EA Ghamande SA Chan JK Barve MA Oh J. Gemogenovatucel-T (Vigil) Immunotherapy as Maintenance in Frontline Stage III/IV Ovarian Cancer (VITAL): A Randomised, Double-Blind, Placebo-Controlled, Phase 2b Trial. Lancet Oncol (2020) 21(12):1661–72. doi: 10.1016/S1470-2045(20)30533-7 33271095

[14] Pfisterer J Shannon CM Baumann K Rau J Harter P Joly F. Bevacizumab and Platinum-Based Combinations for Recurrent Ovarian Cancer: A Randomised, Open-Label, Phase 3 Trial. Lancet Oncol (2020) 21(5):699–709. doi: 10.1016/S1470-2045(20)30142-X 32305099

[15] Gourd E. Olaparib Plus Bevacizumab Improves Progression-Free Survival in Ovarian Cancer. Lancet Oncol (2020) 21(2):e71. doi: 10.1016/S1470-2045(20)30005-X 31928926

[16] Binnewies M Roberts EW Kersten K Chan V Fearon DF Merad M. Understanding the Tumor Immune Microenvironment (TIME) for Effective Therapy. Nat Med (2018) 24(5):541–50. doi: 10.1038/s41591-018-0014-x PMC5998822 29686425

[17] Torphy RJ Schulick RD Zhu Y. Understanding the Immune Landscape and Tumor Microenvironment of Pancreatic Cancer to Improve Immunotherapy. Mol Carcinog (2020) 59(7):775–82. doi: 10.1002/mc.23179 32166821

[18] Zahn LM. Effects of the Tumor Microenvironment. Science (2017) 355(6332):1386–8. doi: 10.1126/science.355.6332.1386-l 28360308

[19] Pelly VS Moeini A Roelofsen LM Bonavita E Bell CR Hutton C. Anti-Inflammatory Drugs Remodel the Tumor Immune Environment to Enhance Immune Checkpoint Blockade Efficacy. Cancer Discov (2021) 11(10):2602–19. doi: 10.1158/2159-8290.CD-20-1815 PMC7611767 34031121

[20] Too NSH Ho NCW Adine C Iyer NG Fong ELS. Hot or Cold: Bioengineering Immune Contextures Into *In Vitro* Patient-Derived Tumor Models. Adv Drug Deliv Rev (2021) 175:113791. doi: 10.1016/j.addr.2021.05.001 33965462

[21] Liu YT Sun ZJ. Turning Cold Tumors Into Hot Tumors by Improving T-Cell Infiltration. Theranostics (2021) 11(11):5365–86. doi: 10.7150/thno.58390 PMC8039952 33859752

[22] Wall JA Meza-Perez S Scalise CB Katre A Londono AI Turbitt WJ. Manipulating the Wnt/beta-Catenin Signaling Pathway to Promote Anti-Tumor Immune Infiltration Into the TME to Sensitize Ovarian Cancer to ICB Therapy. Gynecol Oncol (2021) 160(1):285–94. doi: 10.1016/j.ygyno.2020.10.031 PMC9107782 33168307

[23] Bielski CM Taylor BS. Homing in on Genomic Instability as a Therapeutic Target in Cancer. Nat Commun (2021) 12(1):3663. doi: 10.1038/s41467-021-23965-5 34135330 PMC8209011

[24] Negrini S Gorgoulis VG Halazonetis TD. Genomic Instability–an Evolving Hallmark of Cancer. Nat Rev Mol Cell Biol (2010) 11(3):220–8. doi: 10.1038/nrm2858 20177397

[25] Tian J Lu Z Niu S Zhang S Ying P Wang L. Aberrant MCM10 SUMOylation Induces Genomic Instability Mediated by a Genetic Variant Associated With Survival of Esophageal Squamous Cell Carcinoma. Clin Transl Med (2021) 11(6):e485. doi: 10.1002/ctm2.485 34185429 PMC8236122

[26] Bao S Zhao H Yuan J Fan D Zhang Z Su J. Computational Identification of Mutator-Derived lncRNA Signatures of Genome Instability for Improving the Clinical Outcome of Cancers: A Case Study in Breast Cancer. Brief Bioinform (2020) 21(5):1742–55. doi: 10.1093/bib/bbz118 31665214

[27] Ashworth A Lord CJ. Synthetic Lethal Therapies for Cancer: What's Next After PARP Inhibitors? Nat Rev Clin Oncol (2018) 15(9):564–76. doi: 10.1038/s41571-018-0055-6 29955114

[28] Ott PA Hu Z Keskin DB Shukla SA Sun J Bozym DJ. An Immunogenic Personal Neoantigen Vaccine for Patients With Melanoma. Nature (2017) 547(7662):217–21. doi: 10.1038/nature22991 PMC5577644 28678778

[29] Ott PA Hu Z Keskin DB Shukla SA Sun J Bozym DJ. Corrigendum: An Immunogenic Personal Neoantigen Vaccine for Patients With Melanoma. Nature (2018) 555(7696):402. doi: 10.1038/nature25145 PMC6064631 29542692

[30] Schumacher TN Schreiber RD. Neoantigens in Cancer Immunotherapy. Science (2015) 348(6230):69–74. doi: 10.1126/science.aaa4971 25838375

[31] Desrichard A Snyder A Chan TA. Cancer Neoantigens and Applications for Immunotherapy. Clin Cancer Res (2016) 22(4):807–12. doi: 10.1158/1078-0432.CCR-14-3175 26515495

[32] Mariathasan S Turley SJ Nickles D Castiglioni A Yuen K Wang Y. TGFbeta Attenuates Tumour Response to PD-L1 Blockade by Contributing to Exclusion of T Cells. Nature (2018) 554(7693):544–8. doi: 10.1038/nature25501 PMC6028240 29443960

[33] Yoshihara K Shahmoradgoli M Martinez E Vegesna R Kim H Torres-Garcia W. Inferring Tumour Purity and Stromal and Immune Cell Admixture From Expression Data. Nat Commun (2013) 4:2612. doi: 10.1038/ncomms3612 24113773 PMC3826632

[34] Holleczek B Brenner H. Model Based Period Analysis of Absolute and Relative Survival With R: Data Preparation, Model Fitting and Derivation of Survival Estimates. Comput Methods Programs BioMed (2013) 110(2):192–202. doi: 10.1016/j.cmpb.2012.10.004 23116692

[35] Newman AM Liu CL Green MR Gentles AJ Feng W Xu Y. Robust Enumeration of Cell Subsets From Tissue Expression Profiles. Nat Methods (2015) 12(5):453–7. doi: 10.1038/nmeth.3337 PMC4739640 25822800

[36] Charoentong P Finotello F Angelova M Mayer C Efremova M Rieder D. Pan-Cancer Immunogenomic Analyses Reveal Genotype-Immunophenotype Relationships and Predictors of Response to Checkpoint Blockade. Cell Rep (2017) 18(1):248–62. doi: 10.1016/j.celrep.2016.12.019 28052254

[37] Yi M Nissley DV McCormick F Stephens RM. ssGSEA Score-Based Ras Dependency Indexes Derived From Gene Expression Data Reveal Potential Ras Addiction Mechanisms With Possible Clinical Implications. Sci Rep (2020) 10(1):10258. doi: 10.1038/s41598-020-66986-8 32581224 PMC7314760

[38] Mayakonda A Lin DC Assenov Y Plass C Koeffler HP. Maftools: Efficient and Comprehensive Analysis of Somatic Variants in Cancer. Genome Res (2018) 28(11):1747–56. doi: 10.1101/gr.239244.118 PMC6211645 30341162

[39] Robinson DR Wu YM Lonigro RJ Vats P Cobain E Everett J. Integrative Clinical Genomics of Metastatic Cancer. Nature (2017) 548(7667):297–303. doi: 10.1038/nature23306 28783718 PMC5995337

[40] Chen XY Zhang J Zhu JS. The Role of M(6)A RNA Methylation in Human Cancer. Mol Cancer (2019) 18(1):103. doi: 10.1186/s12943-019-1033-z 31142332 PMC6540575

[41] Liu S Li Q Chen K Zhang Q Li G Zhuo L. The Emerging Molecular Mechanism of M(6)A Modulators in Tumorigenesis and Cancer Progression. BioMed Pharmacother (2020) 127:110098. doi: 10.1016/j.biopha.2020.110098 32299028

[42] Li Y Xiao J Bai J Tian Y Qu Y Chen X. Molecular Characterization and Clinical Relevance of M(6)A Regulators Across 33 Cancer Types. Mol Cancer (2019) 18(1):137. doi: 10.1186/s12943-019-1066-3 31521193 PMC6744659

[43] Alhmoud JF Woolley JF Al Moustafa AE Malki MI. DNA Damage/Repair Management in Cancers. Cancers (Basel) (2020) 12(4):1050. doi: 10.3390/cancers12041050 PMC7226105 32340362

[44] Hoeijmakers JH. DNA Damage, Aging, and Cancer. N Engl J Med (2009) 361(15):1475–85. doi: 10.1056/NEJMra0804615 19812404

[45] Shum B Larkin J Turajlic S. Predictive Biomarkers for Response to Immune Checkpoint Inhibition. Semin Cancer Biol (2021) S1044-579X(21):00097–3. doi: 10.1016/j.semcancer.2021.03.036 33819567

[46] Emmanuel C Chiew YE George J Etemadmoghadam D Anglesio MS Sharma R. Genomic Classification of Serous Ovarian Cancer With Adjacent Borderline Differentiates RAS Pathway and TP53-Mutant Tumors and Identifies NRAS as an Oncogenic Driver. Clin Cancer Res (2014) 20(24):6618–30. doi: 10.1158/1078-0432.CCR-14-1292 25316818

[47] Soragni A Janzen DM Johnson LM Lindgren AG Thai-Quynh Nguyen A Tiourin E. A Designed Inhibitor of P53 Aggregation Rescues P53 Tumor Suppression in Ovarian Carcinomas. Cancer Cell (2016) 29(1):90–103. doi: 10.1016/j.ccell.2015.12.002 26748848 PMC4733364

[48] Barbieri I Kouzarides T. Role of RNA Modifications in Cancer. Nat Rev Cancer (2020) 20(6):303–22. doi: 10.1038/s41568-020-0253-2 32300195

[49] Pardoll DM. The Blockade of Immune Checkpoints in Cancer Immunotherapy. Nat Rev Cancer (2012) 12(4):252–64. doi: 10.1038/nrc3239 PMC4856023 22437870

[50] Hamanishi J Mandai M Ikeda T Minami M Kawaguchi A Murayama T. Safety and Antitumor Activity of Anti-PD-1 Antibody, Nivolumab, in Patients With Platinum-Resistant Ovarian Cancer. J Clin Oncol (2015) 33(34):4015–22. doi: 10.1200/JCO.2015.62.3397 26351349

[51] Varga A Piha-Paul S Ott PA Mehnert JM Berton-Rigaud D Morosky A. Pembrolizumab in Patients With Programmed Death Ligand 1-Positive Advanced Ovarian Cancer: Analysis of KEYNOTE-028. Gynecol Oncol (2019) 152(2):243–50. doi: 10.1016/j.ygyno.2018.11.017 30522700

[52] Nishio S Matsumoto K Takehara K Kawamura N Hasegawa K Takeshima N. Pembrolizumab Monotherapy in Japanese Patients With Advanced Ovarian Cancer: Subgroup Analysis From the KEYNOTE-100. Cancer Sci (2020) 111(4):1324–32. doi: 10.1111/cas.14340 PMC7156846 32012411

[53] Gupta RG Li F Roszik J Lizee G. Exploiting Tumor Neoantigens to Target Cancer Evolution: Current Challenges and Promising Therapeutic Approaches. Cancer Discov (2021) 11(5):1024–39. doi: 10.1158/2159-8290.CD-20-1575 PMC8102318 33722796

[54] Anderson P Aptsiauri N Ruiz-Cabello F Garrido F. HLA Class I Loss in Colorectal Cancer: Implications for Immune Escape and Immunotherapy. Cell Mol Immunol (2021) 18(3):556–65. doi: 10.1038/s41423-021-00634-7 PMC8027055 33473191

[55] Fox EJ Prindle MJ Loeb LA. Do Mutator Mutations Fuel Tumorigenesis? Cancer Metastasis Rev (2013) 32(3-4):353–61. doi: 10.1007/s10555-013-9426-8 PMC3987827 23592419

[56] Svoboda M Meshcheryakova A Heinze G Jaritz M Pils D Castillo-Tong DC. AID/APOBEC-Network Reconstruction Identifies Pathways Associated With Survival in Ovarian Cancer. BMC Genomics (2016) 17(1):643. doi: 10.1186/s12864-016-3001-y 27527602 PMC4986275

[57] Serebrenik AA Argyris PP Jarvis MC Brown WL Bazzaro M Vogel RI. The DNA Cytosine Deaminase APOBEC3B is a Molecular Determinant of Platinum Responsiveness in Clear Cell Ovarian Cancer. Clin Cancer Res (2020) 26(13):3397–407. doi: 10.1158/1078-0432.CCR-19-2786 PMC7334080 32060098

[58] Du Y Tao X Wu J Yu H Yu Y Zhao H. APOBEC3B Up-Regulation Independently Predicts Ovarian Cancer Prognosis: A Cohort Study. Cancer Cell Int (2018) 18:78. doi: 10.1186/s12935-018-0572-5 29853799 PMC5975489

[59] Leonard B Hart SN Burns MB Carpenter MA Temiz NA Rathore A. APOBEC3B Upregulation and Genomic Mutation Patterns in Serous Ovarian Carcinoma. Cancer Res (2013) 73(24):7222–31. doi: 10.1158/0008-5472.CAN-13-1753 PMC3867573 24154874

[60] Leonard B Starrett GJ Maurer MJ Oberg AL Van Bockstal M Van Dorpe J. APOBEC3G Expression Correlates With T-Cell Infiltration and Improved Clinical Outcomes in High-Grade Serous Ovarian Carcinoma. Clin Cancer Res (2016) 22(18):4746–55. doi: 10.1158/1078-0432.CCR-15-2910 PMC5026552 27016308

[61] Wang S Jia M He Z Liu XS. APOBEC3B and APOBEC Mutational Signature as Potential Predictive Markers for Immunotherapy Response in Non-Small Cell Lung Cancer. Oncogene (2018) 37(29):3924–36. doi: 10.1038/s41388-018-0245-9 PMC6053356 29695832

[62] Shi Z Zhao Q Lv B Qu X Han X Wang H. Identification of Biomarkers Complementary to Homologous Recombination Deficiency for Improving the Clinical Outcome of Ovarian Serous Cystadenocarcinoma. Clin Transl Med (2021) 11(5):e399. doi: 10.1002/ctm2.399 34047476 PMC8131501

[63] Revathidevi S Murugan AK Nakaoka H Inoue I Munirajan AK. APOBEC: A Molecular Driver in Cervical Cancer Pathogenesis. Cancer Lett (2021) 496:104–16. doi: 10.1016/j.canlet.2020.10.004 PMC7539941 33038491

[64] Buisson R Langenbucher A Bowen D Kwan EE Benes CH Zou L. Passenger Hotspot Mutations in Cancer Driven by APOBEC3A and Mesoscale Genomic Features. Science (2019) 364(6447):eaaw2872. doi: 10.1126/science.aaw2872 31249028 PMC6731024

[65] Langenbucher A Bowen D Sakhtemani R Bournique E Wise JF Zou L. An Extended APOBEC3A Mutation Signature in Cancer. Nat Commun (2021) 12(1):1602. doi: 10.1038/s41467-021-21891-0 33707442 PMC7952602

[66] Law EK Levin-Klein R Jarvis MC Kim H Argyris PP Carpenter MA. APOBEC3A Catalyzes Mutation and Drives Carcinogenesis In Vivo. J Exp Med (2020) 217(12):e20200261. doi: 10.1084/jem.20200261 32870257 PMC7953736

[67] Chen DS Mellman I. Oncology Meets Immunology: The Cancer-Immunity Cycle. Immunity (2013) 39(1):1–10. doi: 10.1016/j.immuni.2013.07.012 23890059

[68] Chan TA Wolchok JD Snyder A. Genetic Basis for Clinical Response to CTLA-4 Blockade in Melanoma. N Engl J Med (2015) 373(20):1984. doi: 10.1056/NEJMc1508163 26559592

[69] Rizvi NA Hellmann MD Snyder A Kvistborg P Makarov V Havel JJ. Cancer Immunology. Mutational Landscape Determines Sensitivity to PD-1 Blockade in Non-Small Cell Lung Cancer. Science (2015) 348(6230):124–8. doi: 10.1126/science.aaa1348 PMC4993154 25765070

[70] Leapman MS Presley CJ Zhu W Soulos PR Adelson KB Miksad RA. Association of Programmed Cell Death Ligand 1 Expression Status With Receipt of Immune Checkpoint Inhibitors in Patients With Advanced Non-Small Cell Lung Cancer. JAMA Netw Open (2020) 3(6):e207205. doi: 10.1001/jamanetworkopen.2020.7205 32511721 PMC7280954

[71] Zhou C Lin A Cao M Ding W Mou W Guo N. Activation of the DDR Pathway Leads to the Down-Regulation of the TGFbeta Pathway and a Better Response to ICIs in Patients With Metastatic Urothelial Carcinoma. Front Immunol (2021) 12:634741. doi: 10.3389/fimmu.2021.634741 34220801 PMC8253049

[72] Brossel H Fontaine A Hoyos C Jamakhani M Willems M Hamaidia M. Activation of DNA Damage Tolerance Pathways May Improve Immunotherapy of Mesothelioma. Cancers (Basel) (2021) 13(13):3211. doi: 10.3390/cancers13133211 34199066 PMC8269013

[73] Zhou BB Elledge SJ. The DNA Damage Response: Putting Checkpoints in Perspective. Nature (2000) 408(6811):433–9. doi: 10.1038/35044005 11100718

[74] Green AM Landry S Budagyan K Avgousti DC Shalhout S Bhagwat AS. APOBEC3A Damages the Cellular Genome During DNA Replication. Cell Cycle (2016) 15(7):998–1008. doi: 10.1080/15384101.2016.1152426 26918916 PMC4889253

[75] Green AM Budagyan K Hayer KE Reed MA Savani MR Wertheim GB. Cytosine Deaminase APOBEC3A Sensitizes Leukemia Cells to Inhibition of the DNA Replication Checkpoint. Cancer Res (2017) 77(17):4579–88. doi: 10.1158/0008-5472.CAN-16-3394 PMC5581702 28655787

