# Supplementary material for: Comprehensive Analyses Identify APOBEC3A as a Genomic Instability-Associated Immune Prognostic Biomarker in Ovarian Cancer
Source: Front Immunol. 2021 Oct 21;12:749369. doi: 10.3389/fimmu.2021.749369 (PMC8568129; doi:10.3389/fimmu.2021.749369)
Supplement: Supplementary file 1 [file DataSheet_1.pdf]

Supplementary Material

1.1 Supplementary Tables

Table S1. Clinical information of patients in TCGA-OV and ICGC OV-AU datasets.

Clinical Parameters	Subtype	TCGA-OV Samples	ICGC OV-AU Samples
Age	<60	190	50
	⩾60	171	43
Stage	I	1	/
	II	21	/
	III	286	79
	IV	53	14
OS times (months)	<12	62	11
	⩾12	299	82

Notes: TCGA, The Cancer Genome Atlas; ICGC, International Cancer Genome Consortium; OS, Overall survival.

Table S2. The results of immune-related differentially expressed genes (DEGs).

Gene	conMean	treatMean	logFC	pValue	FDR
TSPEAR	0.359972	0.136653	-1.39737	2.64E-08	2.49E-07
SYCP2L	0.207928	0.100134	-1.05415	0.00028	0.000979
CDHR4	1.745065	0.613497	-1.50815	0.009826	0.021175
SULT1C2	1.621802	1.030727	-0.65394	2.87E-05	0.000132
SLC9A9	0.738347	1.597264	1.113231	1.05E-30	4.82E-29
CD68	0.794459	1.856841	1.224806	2.68E-36	1.78E-34
NPM2	0.592847	0.257861	-1.20106	3.05E-07	2.32E-06
ST6GALNAC3	0.683108	0.360941	-0.92035	8.00E-06	4.29E-05
OSMR	6.433393	11.6608	0.858015	1.91E-13	3.51E-12
CFAP70	0.819098	0.524898	-0.642	1.74E-05	8.48E-05
FBN3	4.852862	1.73253	-1.48596	1.63E-12	2.72E-11
ZNF99	0.208317	0.117077	-0.83132	1.79E-06	1.13E-05
RSPH9	1.596742	0.904138	-0.82052	0.001751	0.004786
SEPTIN5	3.906884	2.421345	-0.69021	1.01E-05	5.24E-05
POSTN	9.570291	23.89095	1.319829	1.18E-10	1.59E-09
NHLRC4	0.998503	0.611544	-0.70731	0.000512	0.001652
C1orf158	0.863978	0.302027	-1.51632	8.86E-06	4.68E-05
CCDC81	0.497545	0.183069	-1.44244	3.81E-05	0.000169
TMIGD2	0.100914	0.436627	2.113274	3.79E-34	2.18E-32
TTC25	1.566488	0.847449	-0.88633	1.04E-10	1.41E-09
ABCA6	0.092077	0.160569	0.802274	3.21E-07	2.43E-06

Gene	conMean	treatMean	logFC	pValue	FDR
CFAP100	1.188425	0.452378	-1.39345	1.80E-07	1.44E-06
FAM167A	1.479431	0.972842	-0.60477	0.010086	0.021675
MYOD1	1.210053	0.453719	-1.4152	0.000317	0.001091
SPIB	0.148787	0.29714	0.997895	1.80E-17	4.21E-16
ZNF679	0.423219	0.194353	-1.12272	4.11E-07	3.02E-06
CHRNA4	0.173261	0.028442	-2.60687	2.57E-09	2.94E-08
CRISPLD1	3.126004	2.078664	-0.58866	0.002533	0.006614
SH2D5	0.194863	0.1117	-0.80283	5.76E-06	3.20E-05
NOG	1.668393	0.74132	-1.17029	0.01956	0.037981
PYCARD	9.606856	16.25206	0.758486	3.00E-18	7.24E-17
AQP12B	0.20886	0.049051	-2.0902	0.010249	0.021966
PRR18	0.44793	0.204339	-1.13231	0.005296	0.012471
ITGAX	1.211358	3.352529	1.468624	1.13E-38	8.68E-37
DLEU7	0.182702	0.320521	0.810929	3.93E-16	8.63E-15
ADAMTS8	0.652421	0.261305	-1.32007	1.36E-06	8.88E-06
GPR82	0.313838	0.653048	1.057166	9.78E-22	2.75E-20
TEKT4	0.739754	0.248409	-1.57433	0.000522	0.00168
EFCAB10	1.063362	0.448875	-1.24425	2.10E-08	2.02E-07
TFF2	2.65782	0.988331	-1.42718	0.001107	0.003222
WDR87	0.140138	0.072695	-0.94692	3.38E-06	1.99E-05
SLURP1	1.281996	2.536396	0.984388	0.011412	0.024102
TNFSF13B	2.22479	6.655539	1.580887	2.43E-44	3.70E-42
DNAH12	0.232311	0.101018	-1.20145	0.000488	0.001587
REM2	1.028757	0.687687	-0.58108	5.96E-06	3.30E-05
WDR93	0.770642	0.469999	-0.7134	1.58E-05	7.84E-05
OASL	4.775295	7.447358	0.641139	8.51E-11	1.17E-09
GRID2IP	0.654267	0.35457	-0.88381	0.000447	0.00147
CCDC173	1.379642	0.594608	-1.21428	6.92E-12	1.08E-10
SRGN	31.33119	90.16996	1.525047	1.21E-45	2.45E-43
WDR63	0.582682	0.229291	-1.34553	0.0007	0.002168
MYLK3	0.26614	0.144514	-0.88097	5.53E-05	0.000235
C16orf54	0.327228	0.922793	1.49571	4.39E-37	3.05E-35
CXCL5	1.39056	3.329877	1.259803	0.003342	0.008399
JAKMIP2	0.327775	0.178165	-0.87949	0.012452	0.025979
CAMK1G	1.444418	0.83081	-0.7979	0.000145	0.000548
HTRA4	0.079615	0.226107	1.505893	2.04E-26	7.27E-25
CMKLR1	1.030875	2.039123	0.984079	2.70E-21	7.42E-20
INA	4.280601	1.475514	-1.5366	0.000382	0.001282
SLC16A11	1.700028	0.968261	-0.81209	0.000268	0.000944
ACTA2	36.94142	69.85329	0.919089	8.40E-10	1.03E-08
GJA3	0.310623	0.516056	0.732362	0.00107	0.003128
GIMAP6	1.42239	3.781833	1.410769	4.85E-41	4.61E-39
NELL1	0.551908	0.100171	-2.46197	8.14E-06	4.36E-05
IL4R	3.452214	6.266343	0.860102	2.95E-18	7.13E-17
IGFALS	1.200692	0.787409	-0.60868	7.23E-06	3.92E-05

Gene	conMean	treatMean	logFC	pValue	FDR
CDH11	3.857347	6.263828	0.699436	8.43E-06	4.48E-05
MRC1	0.856743	2.157696	1.332557	5.47E-20	1.40E-18
APOL2	10.77689	18.178	0.754253	3.58E-15	7.38E-14
TUBA3D	1.481381	0.680325	-1.12265	0.000839	0.002538
LRRC34	0.727665	0.454949	-0.67757	6.37E-06	3.49E-05
ARL11	0.474171	0.948003	0.999485	7.38E-24	2.32E-22
FMO1	0.979801	1.769672	0.852921	2.74E-09	3.11E-08
CFAP74	0.278172	0.106928	-1.37934	0.002214	0.005876
AK7	1.313799	0.806666	-0.7037	0.012617	0.026301
GPC3	17.86385	5.60723	-1.67168	0.000483	0.001573
SLC7A10	0.730386	0.334114	-1.12832	6.97E-05	0.000288
MYBPHL	1.398241	0.843826	-0.7286	0.002872	0.007381
CFAP53	2.079232	1.382377	-0.5889	2.22E-05	0.000105
LYSMD2	4.153017	6.452333	0.635661	1.08E-15	2.30E-14
PLPPR4	0.474594	0.880285	0.891277	1.45E-08	1.43E-07
RBM24	1.765151	1.047076	-0.75343	0.002653	0.006889
SLC47A2	0.248627	0.161237	-0.6248	0.000518	0.001669
AGAP2	0.250178	0.525476	1.070671	4.01E-27	1.50E-25
TAS2R19	0.188973	0.120665	-0.64717	8.39E-06	4.47E-05
RTN1	0.677128	1.337965	0.982538	5.62E-19	1.39E-17
SLA	0.663194	1.99695	1.590296	2.07E-46	5.35E-44
KRT16	1.966296	4.015537	1.030113	3.32E-08	3.06E-07
ZNF850	1.313475	0.781996	-0.74816	1.31E-06	8.62E-06
DUSP9	3.229004	1.891371	-0.77166	0.000505	0.001634
GLIS1	0.80458	0.531362	-0.59854	0.020104	0.038835
TCTE1	0.428215	0.178998	-1.25839	1.86E-05	8.98E-05
TMEM59L	1.699671	0.420564	-2.01486	1.70E-11	2.52E-10
ISM2	0.708925	0.328687	-1.10892	4.03E-05	0.000178
TRDN	0.234431	0.140046	-0.74327	0.001905	0.00515
CFAP57	0.919067	0.394427	-1.22041	7.71E-07	5.36E-06
CELSR3	0.895601	0.559958	-0.67754	0.000337	0.001149
HK3	0.57201	1.713771	1.583062	2.68E-36	1.78E-34
LAG3	1.583582	3.970109	1.325986	5.99E-24	1.90E-22
GRM4	0.4529	0.204996	-1.1436	6.00E-07	4.26E-06
ADGRF4	0.150498	0.312983	1.056343	1.39E-09	1.66E-08
ZNF10	1.758573	1.123311	-0.64665	6.27E-12	9.85E-11
DOK3	1.30042	2.701841	1.054966	1.89E-34	1.13E-32
COLEC12	2.024451	3.3099	0.709257	1.21E-10	1.63E-09
FXYD6	8.507765	4.238782	-1.00513	2.54E-07	1.97E-06
LAT2	1.211704	3.25886	1.42733	5.74E-46	1.25E-43
CIB4	0.187541	0.086839	-1.11079	3.80E-06	2.21E-05
OSM	0.877584	2.489129	1.504031	2.79E-24	9.03E-23
RAB31	6.833693	12.85393	0.911472	1.88E-22	5.50E-21
MYT1	0.578127	0.282475	-1.03326	1.10E-06	7.34E-06
SLC13A5	0.847678	0.515209	-0.71836	0.015257	0.030825
ARHGAP9	0.657939	2.039487	1.632181	7.64E-48	2.52E-45

Gene	conMean	treatMean	logFC	pValue	FDR
NIBAN1	1.940315	3.536869	0.866182	7.63E-18	1.81E-16
CYP11A1	1.741696	0.981306	-0.82772	9.66E-06	5.05E-05
EFR3B	0.63563	0.280742	-1.17894	1.05E-11	1.60E-10
WASF1	10.40096	5.694147	-0.86917	5.21E-07	3.75E-06
CD38	0.481399	1.563591	1.699558	5.51E-25	1.84E-23
CD48	1.426227	5.638118	1.98301	1.05E-54	3.71E-51
CCDC33	1.048416	0.657	-0.67425	2.27E-06	1.40E-05
ACVR2B	2.018164	1.236177	-0.70716	3.44E-12	5.57E-11
TGFBI	13.73598	29.79194	1.116962	4.75E-26	1.66E-24
CXCL11	4.236244	13.6058	1.683364	2.65E-25	9.06E-24
GZMB	1.122263	5.10075	2.184299	2.76E-33	1.48E-31
SIX3	5.294023	1.383491	-1.93605	4.67E-08	4.19E-07
RAX2	0.271395	0.125109	-1.1172	0.000253	0.000894
ZBP1	0.72527	1.348409	0.894668	1.76E-13	3.26E-12
KRT6B	0.379021	0.758772	1.001389	0.000815	0.002479
LILRB4	1.25555	4.718977	1.910155	1.77E-48	6.86E-46
PYGO1	0.686323	0.426546	-0.68619	0.003156	0.007992
SLC39A5	0.51232	0.115193	-2.15299	2.09E-07	1.65E-06
SCIMP	0.430953	1.241472	1.52645	1.08E-40	1.01E-38
TMEM252	0.547946	0.147133	-1.89691	0.021537	0.04116
STRA6	5.052852	3.136311	-0.68803	0.00289	0.007415
COL28A1	0.448559	0.176096	-1.34894	3.53E-05	0.000158
C8orf34	0.184917	0.103988	-0.83046	0.001658	0.004566
NCF4	1.829447	4.95382	1.437134	1.74E-48	6.86E-46
SV2A	3.231649	1.821512	-0.82713	0.001435	0.004024
TLR6	0.277459	0.599913	1.112478	6.29E-29	2.56E-27
DUOX2	0.174044	0.112785	-0.62587	0.013577	0.027978
EFHC2	2.039692	1.258737	-0.69637	0.000624	0.001962
CABP1	0.13382	0.083935	-0.67296	0.025681	0.04776
LUM	49.60071	115.1115	1.214599	1.37E-11	2.05E-10
LPAR5	1.893647	3.630532	0.939014	4.64E-22	1.33E-20
HAVCR2	1.857386	5.838874	1.652417	3.45E-51	3.06E-48
CACNA2D4	0.345709	0.620448	0.843753	1.11E-23	3.44E-22
GLRX	1.343586	2.724406	1.019853	4.74E-38	3.55E-36
AL645941.2	0.130883	0.27681	1.080614	7.69E-12	1.20E-10
IL11RA	4.848528	3.072542	-0.65811	0.007606	0.017003
PCYT1B	0.454853	0.220687	-1.0434	0.004004	0.009807
MMP13	1.261966	3.665112	1.538184	1.85E-07	1.48E-06
AIM2	0.370661	1.222511	1.721676	5.69E-36	3.70E-34
GRIK4	0.44827	0.227441	-0.97888	1.74E-05	8.48E-05
GPR25	0.245158	0.586184	1.257639	5.41E-14	1.04E-12
NEFH	5.032091	3.066963	-0.71435	0.025558	0.047586
HAS1	0.248396	0.569974	1.198254	0.000167	0.000623
CCDC170	2.241059	1.412897	-0.66552	0.000335	0.001146
CSF1R	6.478676	19.9099	1.619715	2.43E-36	1.64E-34

Gene	conMean	treatMean	logFC	pValue	FDR
STK17B	5.480969	8.341256	0.605834	3.26E-16	7.20E-15
FN1	54.16565	124.3622	1.199097	1.73E-13	3.19E-12
SLC18A3	0.770211	0.473139	-0.70299	0.000119	0.00046
SLC35D3	0.130246	0.401861	1.625458	2.64E-07	2.04E-06
TMEM253	0.181386	0.095258	-0.92914	3.70E-06	2.16E-05
ADARB2	0.320169	0.096343	-1.73258	1.60E-08	1.57E-07
SCN4B	0.732173	0.460083	-0.67029	0.002149	0.00572
PLCB2	1.326873	2.488393	0.907184	3.86E-21	1.05E-19
FBN1	2.367358	4.508605	0.929403	8.02E-08	6.89E-07
TLR8	0.201793	0.83988	2.05731	9.26E-40	7.77E-38
CD207	0.740893	1.669505	1.172084	0.008589	0.018886
GPR1	0.39607	0.822857	1.054885	8.30E-10	1.02E-08
TLR3	1.639173	2.594994	0.662763	1.85E-12	3.06E-11
MCIDAS	1.081069	0.550438	-0.97381	0.000814	0.002473
GRID2	0.265557	0.094455	-1.49133	3.15E-06	1.87E-05
FZD10	9.095835	4.976811	-0.86998	0.000104	0.000409
ITGB6	2.492895	5.088306	1.029363	1.36E-10	1.82E-09
PLEKHB1	17.47989	11.44819	-0.61058	4.92E-07	3.56E-06
NKAIN1	0.932152	0.421612	-1.14465	5.33E-09	5.78E-08
TMEM154	0.219166	0.391309	0.836282	1.53E-19	3.86E-18
CERKL	0.819311	1.274913	0.637916	7.45E-22	2.11E-20
SCGB1C1	0.949509	0.440021	-1.10961	0.0033	0.008313
FMO2	0.569739	1.799082	1.658889	2.16E-12	3.54E-11
PPIL6	2.086622	1.390749	-0.58531	4.99E-07	3.60E-06
AKR7A3	1.261924	0.789808	-0.67605	0.001854	0.005031
FSTL4	0.748598	0.345101	-1.11717	8.38E-07	5.77E-06
B4GALNT3	4.704625	2.630667	-0.83865	3.79E-10	4.86E-09
NPY2R	0.213733	0.082285	-1.3771	0.000908	0.002717
CTXND1	0.740924	0.16175	-2.19556	2.82E-09	3.21E-08
SSC4D	4.287016	2.608462	-0.71677	2.37E-09	2.73E-08
IRAK2	1.378496	2.439542	0.823515	1.32E-12	2.23E-11
MYO7B	0.674963	0.427651	-0.65838	0.00748	0.016761
FGFBP1	1.600229	2.618155	0.710272	0.006801	0.01545
CCDC138	1.931283	1.25967	-0.61651	1.04E-13	1.94E-12
APOC2	0.111689	0.323885	1.535992	4.47E-26	1.56E-24
GNAO1	1.751826	0.818261	-1.09823	0.000449	0.001474
TMIGD3	1.158603	2.925966	1.336527	8.68E-30	3.77E-28
GBX2	0.164116	0.048152	-1.76905	0.001274	0.003636
SPI1	8.717976	26.95166	1.628309	3.87E-55	2.74E-51
MYO5A	1.143941	1.772468	0.631747	5.88E-15	1.20E-13
VENTX	0.196715	0.484737	1.301094	3.32E-23	1.01E-21
S100A7A	0.19069	0.486179	1.350263	4.00E-07	2.95E-06
MS4A14	0.187947	0.444912	1.243194	4.67E-23	1.41E-21
HKDC1	3.944794	2.561576	-0.62292	0.000298	0.001033
PRSS33	11.85437	3.96802	-1.57893	5.62E-05	0.000238
DMBX1	0.759119	1.38032	0.862605	0.013398	0.027682

Gene	conMean	treatMean	logFC	pValue	FDR
P2RX7	0.958485	1.812748	0.919351	4.71E-27	1.76E-25
SLC3A1	0.323928	0.147312	-1.1368	0.000105	0.000413
FIGNL2	1.406401	0.864732	-0.70168	0.00177	0.004835
FCGBP	3.470674	6.827029	0.976042	2.15E-05	0.000102
RSPH14	0.50574	0.271869	-0.89548	8.56E-09	8.95E-08
NOX4	1.320374	2.017255	0.611446	7.62E-06	4.12E-05
MYLPF	5.520939	3.621628	-0.60828	0.007113	0.016061
CFAP44	0.772241	0.491282	-0.6525	3.37E-07	2.53E-06
CFAP73	2.660061	1.426119	-0.89937	0.000273	0.000958
LRRTM1	20.58715	12.19547	-0.7554	0.018934	0.036989
MAK	1.030338	0.669326	-0.62234	3.53E-05	0.000158
CASP1	2.66974	5.673986	1.087663	1.07E-32	5.43E-31
TLR2	1.622961	4.194078	1.369725	2.33E-40	2.06E-38
SALL2	8.154051	4.603272	-0.82486	1.04E-08	1.06E-07
PPP1R42	0.553486	0.232287	-1.25264	2.34E-06	1.44E-05
NOS1	0.275589	0.176967	-0.63904	0.00076	0.002332
C1S	23.91902	46.97206	0.973645	3.03E-21	8.29E-20
POU3F3	1.565043	0.103137	-3.92357	0.005941	0.013766
DNAH11	0.228729	0.105986	-1.10976	2.83E-08	2.65E-07
SEMA6A	1.909666	0.974981	-0.96987	0.001109	0.003223
RHOU	16.99168	11.34642	-0.58259	1.72E-08	1.67E-07
FERMT3	3.332738	9.736891	1.546754	1.83E-51	1.73E-48
SEMA4G	2.990546	1.60186	-0.90066	1.73E-06	1.09E-05
BIRC3	2.598427	4.509962	0.795477	4.57E-15	9.39E-14
HFM1	0.267662	0.112892	-1.24546	1.11E-09	1.33E-08
SH2B3	3.735042	6.404435	0.777947	7.18E-22	2.04E-20
CARMIL3	0.436499	0.196978	-1.14794	1.75E-10	2.31E-09
ZNF726	1.182231	0.72677	-0.70194	3.36E-09	3.77E-08
MASP1	0.182102	0.046142	-1.98061	6.69E-07	4.72E-06
NALCN	0.147175	0.235164	0.676136	1.50E-06	9.65E-06
FBXO6	8.920763	13.65771	0.614476	1.31E-11	1.97E-10
ADH1B	0.537975	1.877129	1.802916	2.87E-06	1.72E-05
ABCC3	1.198533	1.80787	0.593021	5.39E-09	5.84E-08
PDYN	2.0308	1.302268	-0.64102	0.00606	0.013999
KLK10	50.20504	76.92688	0.615655	1.13E-05	5.80E-05
SPRR1A	1.045041	0.652862	-0.67871	0.002257	0.005976
ATP8B4	0.133434	0.296424	1.15153	2.14E-29	9.04E-28
STAR	1.830956	0.935019	-0.96953	3.68E-06	2.14E-05
RNF157	1.605197	0.796022	-1.01187	2.09E-07	1.65E-06
NR4A3	0.655415	1.500436	1.194901	7.24E-05	0.000298
CCL14	0.064276	0.143905	1.162752	0.003615	0.008977
CTNND2	0.684069	0.371289	-0.8816	0.00555	0.012985
COTL1	10.68851	16.31563	0.610194	2.48E-20	6.54E-19
ARHGDIG	0.66086	0.340022	-0.95872	0.023593	0.044419
ALDH1A3	0.939992	1.684739	0.841804	9.51E-08	8.06E-07

Gene	conMean	treatMean	logFC	pValue	FDR
MLN	0.14099	0.081634	-0.78836	0.000946	0.002813
CASP5	0.054252	0.17262	1.669856	5.72E-28	2.25E-26
CFD	4.858538	13.04799	1.425233	5.95E-25	1.98E-23
GBP3	6.269449	10.39858	0.729976	3.33E-12	5.39E-11
SCGB2B2	0.413368	0.241539	-0.77517	3.78E-05	0.000168
ODF3L1	0.471607	0.309345	-0.60837	0.00023	0.000823
ETS1	3.371958	5.461228	0.695639	1.04E-15	2.22E-14
TNFAIP2	64.84923	100.985	0.638979	7.30E-06	3.96E-05
CLEC4F	0.379328	0.222041	-0.77262	1.14E-05	5.82E-05
C1orf194	8.555842	3.092808	-1.46799	3.47E-07	2.60E-06
ADGRL3	0.583528	0.273767	-1.09185	0.000178	0.000658
ATP1B2	6.155169	2.64485	-1.21861	8.57E-07	5.88E-06
TEKT2	6.196009	3.063541	-1.01614	2.81E-07	2.14E-06
GPAT3	0.490805	0.747583	0.607084	1.67E-11	2.48E-10
CLEC10A	0.279101	1.24575	2.158157	7.80E-32	3.81E-30
TPSD1	0.51705	1.096232	1.084176	7.03E-05	0.00029
VMO1	1.705843	3.144859	0.88251	5.43E-20	1.39E-18
CACNG6	0.814587	0.212909	-1.93583	0.011399	0.024089
VASH2	0.857684	0.558494	-0.61891	0.000505	0.001634
FOXA2	5.196147	2.247999	-1.2088	0.000988	0.002924
PYHIN1	0.111827	0.497999	2.154878	1.20E-40	1.11E-38
FRAS1	1.774843	1.166543	-0.60545	4.79E-06	2.72E-05
HPSE2	0.985309	0.09108	-3.43537	1.89E-08	1.83E-07
HIVEP3	0.568484	0.94033	0.726047	2.84E-14	5.55E-13
MAGEE2	0.187557	0.094265	-0.99254	0.000936	0.002788
RPRM	2.477784	1.280387	-0.95247	3.93E-09	4.38E-08
TESPA1	0.090438	0.33551	1.891346	1.10E-46	2.95E-44
MS4A4A	1.943333	5.473052	1.493812	2.35E-37	1.66E-35
LINGO4	0.295256	0.194515	-0.60209	0.022855	0.043243
SOWAHD	0.541815	0.889131	0.714595	1.24E-17	2.92E-16
SLC7A7	2.958846	4.837341	0.70918	9.04E-35	5.52E-33
CCDC80	5.197448	9.851885	0.922596	1.48E-08	1.46E-07
CTSS	9.301059	24.59614	1.402965	4.79E-41	4.59E-39
CCL3L1	1.288259	2.50386	0.958732	3.16E-14	6.17E-13
SEMA5B	6.119882	3.13211	-0.96637	8.07E-06	4.32E-05
TIMP4	0.812235	1.66503	1.035579	1.01E-07	8.54E-07
DLK1	20.11822	1.39409	-3.85111	9.43E-08	8.00E-07
ARHGAP45	2.492021	4.851572	0.961136	6.86E-27	2.52E-25
GBP6	0.084061	0.159717	0.926001	4.78E-13	8.37E-12
FLVCR2	0.637857	1.011625	0.665369	1.06E-16	2.39E-15
PCED1B	2.429434	3.663679	0.592673	1.96E-20	5.19E-19
PLXNA4	1.703674	0.660261	-1.36754	0.000343	0.001166
SFRP4	8.991441	25.61228	1.510211	3.61E-13	6.39E-12
TTC23L	0.308243	0.16827	-0.87329	3.92E-11	5.58E-10
CA14	1.066964	0.504052	-1.08187	9.06E-10	1.10E-08
FTH1	860.0183	1316.721	0.61451	1.20E-12	2.03E-11

Gene	conMean	treatMean	logFC	pValue	FDR
P2RY10	0.141672	0.76861	2.439694	3.27E-43	4.25E-41
APOBEC3G	2.730302	5.055484	0.888789	2.85E-21	7.80E-20
DACT2	4.088464	1.825726	-1.16309	5.24E-09	5.69E-08
CBR3	2.349336	3.853512	0.713921	2.22E-13	4.05E-12
GNAZ	2.927119	1.93783	-0.59504	2.15E-06	1.33E-05
FAM155A	0.170591	0.258136	0.597592	4.16E-07	3.06E-06
SLC38A5	1.890263	3.438619	0.863242	8.10E-12	1.25E-10
BAMBI	4.294582	2.005821	-1.09833	1.14E-05	5.84E-05
LIPA	11.18885	16.76707	0.583569	2.53E-15	5.29E-14
OPRK1	0.247137	0.162181	-0.60771	0.00072	0.002223
TRIM71	0.651003	0.283718	-1.19821	1.85E-06	1.17E-05
ERAP2	3.510691	5.420675	0.626717	3.29E-05	0.000149
IL18	5.068261	7.606752	0.58579	2.61E-13	4.70E-12
P2RY13	0.567596	1.840355	1.697048	7.36E-33	3.82E-31
PACRG	1.373267	0.668098	-1.03948	4.19E-09	4.65E-08
CFAP65	0.368952	0.154179	-1.25883	8.15E-10	1.00E-08
CFAP47	0.355403	0.174505	-1.02619	1.40E-05	7.02E-05
MYO3B	1.170878	0.779705	-0.58659	0.002115	0.005642
ABTB2	3.083688	1.830566	-0.75237	4.48E-05	0.000195
FIGN	1.957599	1.200337	-0.70565	4.28E-05	0.000188
PRTG	0.2349	0.107987	-1.12119	1.69E-05	8.31E-05
FABP4	1.929816	6.695299	1.794685	0.001201	0.003455
ADRB2	0.165577	0.306666	0.889165	2.12E-15	4.44E-14
ANO1	8.905888	4.334461	-1.03891	0.008877	0.019448
PDE4B	0.701836	1.590049	1.179866	1.07E-29	4.64E-28
BMP7	25.9338	13.68986	-0.92173	8.73E-07	5.98E-06
C9orf24	8.915642	3.887834	-1.19737	0.000186	0.000684
BCL11B	0.200494	0.306444	0.612063	1.65E-12	2.75E-11
KIF5A	1.533053	0.949013	-0.69191	4.32E-05	0.000189
RBP2	0.705305	0.447353	-0.65683	0.000414	0.001377
TMEM176A	11.56796	19.85013	0.779014	2.57E-15	5.36E-14
VSIR	7.492258	12.66372	0.757229	4.13E-18	9.91E-17
RAPSN	0.760256	0.404998	-0.90857	0.000121	0.000467
WNT8B	0.299199	0.144237	-1.05267	0.01219	0.025519
FBXO17	6.788365	3.810035	-0.83326	6.24E-07	4.42E-06
TMEM132B	0.70316	0.202599	-1.79523	4.02E-10	5.14E-09
TMSB15A	7.646376	2.164901	-1.82048	7.18E-05	0.000296
UPP1	2.24598	3.729051	0.731464	2.31E-13	4.20E-12
MATN4	0.833102	0.213921	-1.96142	8.67E-08	7.40E-07
ALDH3A1	1.738591	1.063217	-0.70948	2.27E-07	1.78E-06
RADIL	0.699381	0.419621	-0.73699	0.002719	0.007039
RNASE1	176.0253	274.1773	0.639327	9.97E-12	1.52E-10
SLA2	0.288979	1.040511	1.848257	5.74E-46	1.25E-43
TP73	1.093561	0.691317	-0.66161	0.004981	0.011841
EPPIN	0.138838	0.08226	-0.75514	0.006021	0.013924

Gene	conMean	treatMean	logFC	pValue	FDR
SNX20	0.316831	1.270944	2.004114	1.38E-51	1.39E-48
IL15	0.615019	1.447174	1.234535	1.32E-24	4.28E-23
TLL2	0.158576	0.072107	-1.13697	0.00293	0.00751
GYPC	5.866552	8.957828	0.610636	4.89E-17	1.13E-15
TMEM52B	0.189196	0.572622	1.597703	3.74E-39	2.99E-37
CACNA2D2	3.684197	1.337243	-1.46209	2.05E-12	3.37E-11
HGFAC	1.081093	0.609387	-0.82706	1.71E-06	1.08E-05
PON1	1.298023	0.679073	-0.93468	0.000465	0.00152
BST1	2.574821	4.567402	0.826902	6.15E-15	1.25E-13
MCUB	5.45088	8.469165	0.635731	2.50E-12	4.07E-11
ALOX5	4.348043	10.39827	1.257906	1.83E-28	7.26E-27
MYOM3	0.368547	0.206332	-0.83689	0.007638	0.017066
ABI3	2.143772	6.656652	1.634645	4.83E-54	1.37E-50
DNAH10	0.213263	0.124181	-0.78018	8.83E-05	0.000355
TMEM238L	0.238169	0.093674	-1.34627	0.009869	0.021251
HCST	7.318767	17.02954	1.218367	1.82E-35	1.15E-33
IL7R	0.6218	2.149656	1.789585	1.62E-33	8.97E-32
NTN4	8.034261	12.18435	0.600792	1.74E-07	1.40E-06
FGF1	0.467734	0.767233	0.713977	9.47E-07	6.43E-06
TMEM71	0.387369	0.645444	0.736583	2.33E-19	5.83E-18
ATP1A2	1.176281	0.265546	-2.1472	6.62E-08	5.79E-07
SLC30A3	0.226624	0.094102	-1.268	0.020103	0.038835
CD86	1.466902	4.884888	1.735553	2.07E-52	2.67E-49
LRRC10B	3.329037	1.554078	-1.09905	5.38E-06	3.01E-05
CXCL6	0.854677	1.64713	0.946504	1.46E-08	1.44E-07
MATK	0.561923	1.027136	0.870182	2.41E-26	8.54E-25
PLD4	0.73819	1.822279	1.303679	5.24E-18	1.25E-16
PDE3A	4.688624	3.087985	-0.6025	0.001622	0.004478
SPINK5	1.13415	0.625293	-0.85901	4.52E-09	5.00E-08
MSR1	2.227749	6.077337	1.447853	6.49E-38	4.76E-36
XDH	0.921951	1.532041	0.732694	0.001052	0.003086
TCIRG1	15.97711	24.36623	0.608877	1.51E-15	3.18E-14
TNFRSF19	7.805849	4.013377	-0.95974	8.94E-07	6.10E-06
VWA5B2	0.422767	0.175166	-1.27114	5.41E-08	4.78E-07
TMEM86A	1.541444	2.350318	0.608574	1.44E-16	3.22E-15
CALHM6	3.723063	9.794694	1.39551	3.02E-31	1.43E-29
CHGA	4.317555	0.837448	-2.36614	0.001968	0.005297
KCNJ10	0.204174	0.318289	0.640539	9.04E-05	0.000362
KCNMB2	1.058972	0.589367	-0.84543	5.69E-05	0.000241
PIP5K1B	0.777228	0.374518	-1.0533	0.004058	0.009914
CADPS	0.322066	0.191326	-0.75132	0.000789	0.002407
GBP2	4.879737	11.62307	1.252115	2.35E-32	1.18E-30
KCNG1	3.468308	1.715933	-1.01524	4.87E-09	5.34E-08
MST1	3.49633	2.119948	-0.72181	1.30E-07	1.07E-06
PLEK2	2.62999	4.148054	0.657377	1.78E-14	3.54E-13
GIMAP5	0.084495	0.273113	1.692555	3.44E-34	1.99E-32

Gene	conMean	treatMean	logFC	pValue	FDR
CNNM1	0.289803	0.097782	-1.56743	0.000362	0.001225
CNIH2	3.420844	2.071902	-0.7234	3.13E-05	0.000143
BSN	0.675921	0.313228	-1.10964	4.23E-11	6.01E-10
FAM110B	3.646865	2.252977	-0.69482	2.05E-08	1.98E-07
NAALAD2	0.348482	0.191655	-0.86257	0.004094	0.009994
PAPPA	0.106245	0.19408	0.869253	3.45E-06	2.03E-05
CXCL8	6.871517	12.19152	0.827177	4.66E-07	3.38E-06
RAB9B	0.444959	0.2817	-0.65951	0.002479	0.006489
HEPH	1.134495	1.786723	0.655266	1.22E-07	1.01E-06
DLX4	0.944965	0.572774	-0.72229	0.000455	0.001493
ABCG5	0.178708	0.097668	-0.87165	4.98E-09	5.45E-08
GEM	1.681142	2.538959	0.594795	1.91E-06	1.20E-05
MYO1F	1.086015	2.815537	1.374366	5.89E-38	4.37E-36
DRC1	1.612439	0.697823	-1.20831	9.88E-05	0.000391
S100A12	0.433642	0.932946	1.105287	1.44E-11	2.15E-10
SLC2A3	3.885928	6.836901	0.815083	4.97E-07	3.59E-06
WARS1	21.83302	32.84614	0.589213	8.67E-11	1.19E-09
SLC6A20	0.91609	0.394668	-1.21485	0.00085	0.002568
NCKAP1L	0.827216	2.685165	1.698675	4.94E-45	8.14E-43
SPATA17	1.14389	0.628265	-0.8645	2.95E-09	3.33E-08
C1orf162	4.236546	10.28583	1.279697	1.32E-42	1.56E-40
MAF	3.833037	6.041073	0.656317	1.45E-15	3.06E-14
SLC4A8	0.611088	0.38839	-0.65387	9.41E-08	7.99E-07
C9orf92	0.628106	0.356333	-0.81778	0.0013	0.003699
MCEMP1	0.168762	0.374682	1.150672	3.16E-09	3.57E-08
HLA-F	5.357011	12.07892	1.172991	3.52E-26	1.24E-24
CD44	5.491076	10.13289	0.883886	1.09E-21	3.05E-20
IL18RAP	0.065932	0.237681	1.84997	9.64E-38	6.97E-36
NTM	0.680963	1.373622	1.012337	1.45E-10	1.93E-09
TECTA	0.368785	0.182958	-1.01127	0.015534	0.0313
NKX6-2	0.300783	0.113257	-1.40912	0.003541	0.008824
HPCAL4	0.267747	0.117139	-1.19265	6.96E-05	0.000288
TPSB2	1.049106	1.933218	0.881843	6.76E-11	9.42E-10
ALPP	9.098287	3.344571	-1.44377	0.001477	0.004128
PURG	0.289111	0.133844	-1.11107	1.63E-06	1.04E-05
ACTL6B	0.303051	0.014061	-4.42975	0.000845	0.002553
SERPINA6	0.582053	0.112821	-2.36712	0.021636	0.041327
DUSP26	0.345628	0.169055	-1.03173	0.014526	0.029637
PCDHA7	0.125026	0.075635	-0.7251	0.002735	0.007077
MARCHF1	0.650391	1.432676	1.139333	1.02E-36	7.01E-35
CD7	1.113166	4.116471	1.88674	3.88E-40	3.42E-38
FGF7	0.400787	0.910811	1.184317	4.46E-14	8.64E-13
ADAM12	1.851462	3.92841	1.085281	9.56E-09	9.79E-08
ELOVL2	0.778817	0.384672	-1.01766	0.000177	0.000656
SPOCK3	0.987093	0.417568	-1.24118	0.007604	0.017003

Gene	conMean	treatMean	logFC	pValue	FDR
CCL21	3.362454	11.91935	1.825719	1.42E-06	9.20E-06
BDKRB1	0.135026	0.20877	0.62867	1.21E-05	6.14E-05
CLDN9	13.75427	7.188984	-0.93602	3.95E-07	2.92E-06
TYMP	11.27437	33.35021	1.564649	4.49E-43	5.73E-41
C15orf48	6.230182	13.08332	1.070383	3.30E-19	8.23E-18
MNDA	1.59655	5.582373	1.805921	3.65E-50	2.07E-47
CKB	83.04361	47.20025	-0.81507	9.61E-08	8.13E-07
ITGAD	0.088332	0.179972	1.026767	2.15E-08	2.07E-07
CCDC74B	1.836095	0.917935	-1.00018	1.12E-08	1.13E-07
EML6	0.185602	0.111166	-0.7395	4.49E-08	4.03E-07
INHBE	0.393993	0.222042	-0.82734	4.60E-05	0.0002
UCP1	0.178205	0.110291	-0.69223	0.000259	0.000914
EPYC	3.795484	11.51431	1.601073	1.79E-10	2.37E-09
MT2A	107.7304	182.8591	0.763307	7.09E-13	1.22E-11
CD33	0.313414	0.874069	1.479677	1.95E-45	3.69E-43
TGM3	0.24433	0.138499	-0.81895	0.000468	0.00153
ZMYND10	4.688866	2.1553	-1.12135	4.20E-05	0.000185
HHIPL2	0.508386	0.147148	-1.78865	0.002475	0.006481
ADAM19	1.156916	2.274559	0.975303	3.54E-18	8.51E-17
MARCO	1.318128	4.311323	1.709641	3.58E-16	7.88E-15
CCR8	0.281398	0.424371	0.592716	1.32E-19	3.34E-18
HPGDS	0.544475	1.174629	1.109267	3.22E-19	8.04E-18
ITK	0.079683	0.383782	2.267937	3.02E-46	7.38E-44
DOK2	1.291666	3.662138	1.503453	3.79E-44	5.60E-42
KLHL32	0.859093	0.510481	-0.75096	4.44E-06	2.54E-05
MMP19	1.819048	3.480526	0.936122	2.27E-17	5.30E-16
RENBP	3.066529	5.319491	0.794682	4.79E-19	1.19E-17
PDE11A	0.604071	0.401546	-0.58915	0.008828	0.019363
PDZK1IP1	37.09506	74.38445	1.003774	5.77E-14	1.11E-12
CIITA	2.271944	5.151896	1.181176	5.53E-19	1.37E-17
ALPL	61.17052	32.84247	-0.89727	3.03E-06	1.81E-05
VWA3B	0.443247	0.21504	-1.04351	0.009451	0.020501
C22orf23	0.565337	0.362229	-0.64221	1.40E-07	1.15E-06
PCDHA5	0.126201	0.081061	-0.63864	7.41E-05	0.000304
SEC14L5	0.253618	0.086105	-1.55849	2.85E-08	2.67E-07
DMTN	12.96789	8.028902	-0.69167	1.33E-07	1.09E-06
ABHD12B	0.349742	0.180571	-0.95372	0.016645	0.033189
PF4	0.160414	1.291513	3.009194	0.002024	0.005427
FLRT3	2.941145	1.515285	-0.95679	4.53E-05	0.000197
DNAI1	1.2416	0.580708	-1.09632	1.70E-07	1.37E-06
CYTIP	0.717401	2.227102	1.634317	1.15E-44	1.81E-42
NREP	15.72885	10.32041	-0.60791	0.000128	0.000492
ZMAT4	0.157887	0.073498	-1.10311	0.00517	0.01222
ADAMDEC1	0.833061	4.364588	2.389351	3.08E-31	1.46E-29
TBX21	0.085254	0.375272	2.138102	7.70E-43	9.40E-41
IL2RA	0.399791	1.230813	1.622292	9.58E-35	5.83E-33

Gene	conMean	treatMean	logFC	pValue	FDR
MUC3A	0.169741	0.306983	0.854819	0.014792	0.030053
CH25H	0.654857	1.260621	0.944882	5.15E-15	1.05E-13
ZNF682	1.560104	1.034311	-0.59297	1.11E-10	1.50E-09
HSPB6	27.51225	15.32368	-0.84431	0.003322	0.008356
LYL1	1.103684	2.007907	0.863365	3.60E-29	1.50E-27
KCNH8	0.349263	0.199704	-0.80645	1.02E-05	5.30E-05
KCNH3	3.765136	1.878851	-1.00285	8.35E-10	1.02E-08
IQUB	0.245596	0.09701	-1.34009	1.71E-06	1.08E-05
ANKRD45	2.344355	1.17084	-1.00165	4.17E-10	5.31E-09
CST7	3.698734	10.33415	1.482317	9.32E-36	6.03E-34
ZNF660	1.10724	0.668348	-0.7283	4.24E-14	8.23E-13
CSAG2	0.227469	0.450746	0.986646	1.30E-05	6.56E-05
TET1	0.932852	0.462506	-1.01217	9.90E-12	1.52E-10
PSMB10	10.2438	19.6717	0.94137	3.73E-25	1.26E-23
HOPX	1.875159	3.750061	0.999901	1.69E-13	3.13E-12
PLAUR	6.518998	11.92755	0.871576	7.04E-23	2.10E-21
MAP1B	5.38437	3.50161	-0.62076	0.001324	0.003754
TMEM200A	0.645532	1.111053	0.783367	1.93E-12	3.18E-11
STX11	0.769027	2.289317	1.573812	3.74E-46	8.99E-44
SLCO1A2	0.40609	0.167638	-1.27645	2.76E-07	2.11E-06
TNFSF10	33.31352	59.70242	0.841682	3.82E-15	7.88E-14
IGFN1	0.208465	0.0326	-2.67688	3.65E-06	2.13E-05
KCNN1	1.266683	0.591221	-1.09929	0.006355	0.014561
NINL	3.507551	2.306158	-0.60497	2.30E-08	2.19E-07
DNAH7	0.260785	0.135816	-0.94121	1.63E-09	1.92E-08
SDS	2.140998	4.984671	1.219215	1.77E-28	7.06E-27
ZC3H12B	0.351142	0.224946	-0.64248	0.000174	0.000646
IL12A	1.106054	1.780969	0.68724	2.52E-06	1.53E-05
FAP	1.004136	2.450598	1.287179	4.12E-13	7.24E-12
TCF24	0.215998	0.133495	-0.69423	0.000523	0.001683
ST8SIA2	1.20615	0.553374	-1.12408	0.000486	0.001582
CTLA4	0.160894	0.774847	2.267799	1.29E-40	1.19E-38
C1QTNF9B	0.313457	0.092179	-1.76576	1.11E-06	7.38E-06
CCDC146	18.48308	8.530288	-1.11554	1.22E-07	1.01E-06
IQSEC3	4.427613	2.825877	-0.64783	0.012953	0.026886
COL5A1	20.53262	39.57965	0.946841	4.12E-06	2.37E-05
SEZ6	1.398787	0.303358	-2.20508	0.004118	0.010034
TTLL9	0.685304	0.381027	-0.84685	8.61E-09	8.99E-08
CFAP77	1.753429	0.592374	-1.5656	7.54E-05	0.000309
ASPN	2.355894	3.698144	0.650527	3.37E-07	2.53E-06
GPR171	0.201018	0.884551	2.137622	5.15E-38	3.84E-36
VCX	0.335948	0.141482	-1.24762	4.60E-05	0.0002
PNMA3	4.153835	1.987079	-1.06379	3.28E-13	5.82E-12
CLDN2	0.28471	0.062466	-2.18836	0.00061	0.001927
CCDC65	2.955398	1.692428	-0.80426	1.01E-08	1.03E-07

Gene	conMean	treatMean	logFC	pValue	FDR
XCR1	0.042185	0.165414	1.971272	3.48E-27	1.31E-25
SLC34A3	0.517432	0.253916	-1.02702	2.50E-06	1.52E-05
TRARG1	0.081423	0.380965	2.226155	0.00064	0.002004
CXCL1	8.828949	16.67234	0.917143	2.61E-09	2.99E-08
LRTM2	0.462493	0.0479	-3.27132	1.16E-08	1.17E-07
NUPR1	12.71268	20.06349	0.658304	1.15E-09	1.37E-08
ORM1	0.714982	1.375331	0.9438	0.000604	0.001912
CNTFR	9.123331	5.695954	-0.67962	1.27E-07	1.05E-06
AGTR1	0.187688	0.318957	0.765023	4.25E-06	2.44E-05
RIBC1	2.298085	1.44902	-0.66535	7.59E-05	0.00031
MMP9	8.439042	33.03664	1.968916	2.52E-23	7.66E-22
ITGB7	0.714923	1.329487	0.89501	2.16E-16	4.80E-15
CTSK	20.11946	43.2987	1.105732	2.17E-14	4.29E-13
PCDHB3	1.57385	0.79072	-0.99306	0.001038	0.003051
TMEM38A	6.830471	4.181644	-0.70791	8.48E-07	5.83E-06
THEMIS	0.065859	0.327031	2.311976	1.39E-40	1.27E-38
GPR65	0.423544	1.380975	1.705105	4.51E-46	1.06E-43
IGSF6	1.152355	3.226137	1.485222	4.75E-45	8.01E-43
WAS	2.707252	7.373475	1.445515	6.22E-48	2.15E-45
GDF6	0.215109	0.333296	0.631736	0.000989	0.002928
CPA2	13.2278	0.192134	-6.10532	1.18E-06	7.83E-06
ITGBL1	0.261506	0.611935	1.226536	2.08E-11	3.04E-10
LHX1	11.70361	4.439415	-1.39851	1.69E-06	1.07E-05
WSCD1	0.262433	0.136541	-0.94261	0.025619	0.047676
MCHR1	0.118694	0.536068	2.175176	1.81E-05	8.79E-05
PRAM1	0.520191	0.917286	0.818332	1.11E-16	2.50E-15
LILRB3	0.314129	0.731656	1.21981	2.36E-33	1.28E-31
ST8SIA3	0.222147	0.014376	-3.94975	1.31E-08	1.31E-07
AOC3	1.435293	2.267888	0.660004	2.25E-08	2.16E-07
COPZ2	3.59361	5.842619	0.701181	3.64E-10	4.67E-09
GFRA2	1.094092	0.381247	-1.52094	0.003071	0.00781
CORO1A	5.520445	12.88547	1.222888	2.00E-34	1.19E-32
UBE2U	0.160536	0.077856	-1.04401	0.006617	0.015084
FOXP3	0.820113	2.181629	1.411512	3.40E-34	1.97E-32
CD74	665.6254	1512.714	1.184357	3.71E-28	1.46E-26
GALNT9	0.774882	0.242797	-1.67423	0.013331	0.027563
ODAM	0.24254	0.071598	-1.76022	0.005545	0.012975
SELPLG	3.578884	10.54321	1.558732	4.39E-45	7.49E-43
SHISA8	0.532687	0.320546	-0.73275	0.00782	0.017422
ZNF676	1.030055	0.550599	-0.90365	0.000384	0.001288
CCN5	0.552918	0.914304	0.725609	0.020054	0.038754
SLC16A3	6.031036	9.471679	0.651214	3.42E-13	6.05E-12
LRRC43	1.334364	0.782729	-0.76957	2.17E-06	1.34E-05
CCDC74A	7.14278	4.645175	-0.62075	2.85E-05	0.000131
CECR2	0.596695	0.263257	-1.18052	7.56E-06	4.09E-05
DTNA	1.319475	0.649353	-1.02289	3.81E-05	0.000169

Gene	conMean	treatMean	logFC	pValue	FDR
C1R	28.10157	44.87367	0.675219	7.50E-17	1.70E-15
SLAMF6	0.316052	1.407985	2.155399	9.36E-42	9.90E-40
CD300LF	0.364042	1.335326	1.875014	8.18E-49	3.41E-46
COL8A1	3.858759	8.802493	1.189775	2.10E-10	2.75E-09
CFAP43	1.111382	0.597661	-0.89496	1.36E-08	1.35E-07
RGS4	1.37336	2.73112	0.991783	2.28E-08	2.18E-07
UPK1A	1.838023	0.849702	-1.11313	0.014563	0.029701
C7orf57	1.869375	0.756692	-1.30478	0.001807	0.00492
GPC5	0.472875	0.267806	-0.82027	0.022633	0.042902
BRINP2	0.615238	0.215865	-1.51101	3.14E-05	0.000143
SPAG8	1.980217	1.200384	-0.72216	4.48E-07	3.27E-06
CD3E	1.105592	5.369645	2.280008	1.12E-47	3.60E-45
UNC119B	9.942262	6.362047	-0.64408	6.14E-13	1.06E-11
KCNA3	0.114536	0.336914	1.556582	3.90E-24	1.25E-22
CRHR2	0.171938	0.09629	-0.83643	0.007521	0.016836
AGR3	7.046352	3.11566	-1.17734	2.89E-07	2.21E-06
C3AR1	3.348786	10.82642	1.692846	1.98E-45	3.69E-43
TYROBP	54.42428	158.465	1.541842	7.43E-49	3.28E-46
TSNAXIP1	0.685248	0.398333	-0.78265	5.69E-10	7.12E-09
IKZF1	0.500051	1.508503	1.59297	3.98E-40	3.48E-38
ENO4	0.384157	0.226285	-0.76356	1.07E-08	1.09E-07
SOX18	19.29708	29.09771	0.592523	1.54E-06	9.88E-06
PPP1R1A	1.897867	1.177988	-0.68805	0.010644	0.022688
SRCIN1	0.856151	0.560705	-0.61062	1.92E-05	9.25E-05
SULT1E1	0.445674	0.272858	-0.70784	0.000193	0.000707
SOX21	0.753563	0.180332	-2.06307	0.006078	0.01403
PAEP	8.061427	23.61824	1.550794	8.46E-05	0.000342
PLEKHO2	6.761839	11.78797	0.801828	1.70E-28	6.79E-27
SELENOM	17.90377	27.84947	0.637386	7.42E-08	6.43E-07
EFCAB1	1.487329	0.454172	-1.71141	0.000743	0.002284
PRB3	0.430475	0.173084	-1.31446	0.000211	0.000764
MS4A1	0.045023	0.295413	2.714015	8.58E-25	2.82E-23
HLA-DQA1	8.010353	28.09167	1.810205	4.62E-40	3.97E-38
FAM181B	1.038462	0.550626	-0.9153	0.010389	0.02222
CYP24A1	0.548331	0.83565	0.607851	5.28E-08	4.68E-07
RNASE6	6.195626	18.25244	1.558768	1.06E-43	1.51E-41
FKBP5	3.923042	5.875225	0.582671	1.86E-07	1.49E-06
SPTBN4	0.529007	0.275139	-0.94312	0.000396	0.001326
TEX45	0.993368	0.599215	-0.72926	5.78E-06	3.21E-05
COLEC11	5.499892	2.348557	-1.22763	3.69E-08	3.37E-07
KIAA0895	1.890273	1.212538	-0.64056	3.58E-12	5.79E-11
IGDCC3	1.433001	0.128956	-3.47409	8.01E-09	8.41E-08
C4orf54	0.44789	0.123185	-1.86232	4.61E-09	5.08E-08
CSTL1	0.176678	0.113526	-0.6381	0.006138	0.014142
FGD2	0.728052	1.350113	0.890968	1.31E-21	3.64E-20

Gene	conMean	treatMean	logFC	pValue	FDR
SLC29A4	2.760075	1.536971	-0.84462	0.014907	0.03023
TCL1A	0.163884	0.256764	0.647768	7.80E-14	1.48E-12
LGSN	0.470079	0.210433	-1.15954	2.80E-07	2.14E-06
CD1A	0.21218	1.288594	2.60244	2.04E-21	5.65E-20
LGALS14	0.259853	0.405718	0.642786	0.000777	0.002378
SPIRE2	2.031497	1.069383	-0.92576	4.62E-11	6.53E-10
SCN11A	0.128588	0.074199	-0.79329	5.94E-07	4.23E-06
CASC1	2.74113	1.199857	-1.19191	6.48E-09	6.94E-08
TCEAL5	1.891653	0.680776	-1.4744	1.04E-05	5.40E-05
TTLL10	0.893485	0.488498	-0.87109	2.87E-05	0.000132
LYZ	13.83425	74.59471	2.430829	1.27E-41	1.30E-39
FAM117B	6.018127	3.946807	-0.60863	1.47E-13	2.73E-12
JAKMIP1	0.088496	0.189841	1.101112	5.36E-21	1.45E-19
MEDAG	1.551005	2.862177	0.88391	4.65E-06	2.64E-05
RP1	0.189166	0.112927	-0.74427	0.000352	0.001195
TUBA3E	0.35631	0.079488	-2.16432	0.011859	0.024945
ADGRG5	0.200525	0.451888	1.172184	1.06E-23	3.30E-22
GRAP	0.124795	0.199973	0.680246	3.08E-12	4.99E-11
FRZB	12.61759	5.882823	-1.10086	0.018604	0.03644
CFAP54	0.288068	0.180963	-0.67071	0.000136	0.00052
HLA-A	189.0387	337.2456	0.835118	3.47E-21	9.44E-20
PTGDR	0.05282	0.154861	1.551822	1.40E-30	6.34E-29
FREM2	0.331403	0.063318	-2.38789	5.66E-07	4.05E-06
GAREM2	3.004437	1.785142	-0.75106	0.000664	0.002069
CASR	0.228438	0.14667	-0.63923	1.61E-06	1.03E-05
AEBP1	49.4041	90.86265	0.879057	2.30E-07	1.80E-06
C19orf44	3.537421	2.320997	-0.60795	1.27E-09	1.51E-08
HRK	0.860792	0.560079	-0.62004	0.000576	0.001835
SERPINI2	1.82801	0.589696	-1.63223	2.03E-05	9.71E-05
CCDC78	2.295318	1.464743	-0.64805	9.58E-05	0.000381
WFIKKN2	0.380454	0.159244	-1.25648	1.22E-05	6.21E-05
HLA-C	228.0127	456.9122	1.002803	1.68E-21	4.66E-20
NCF2	1.584468	4.426577	1.482193	3.83E-42	4.21E-40
PTAFR	3.020319	6.518972	1.109944	5.97E-29	2.45E-27
FGL2	2.25097	8.026652	1.834251	1.13E-45	2.33E-43
SMTNL2	1.164778	0.45665	-1.35089	0.001925	0.005198
SECTM1	10.23104	20.28313	0.987328	2.71E-15	5.64E-14
TRAF1	1.096886	1.69854	0.630882	9.23E-15	1.86E-13
PCDHGC4	0.139671	0.078976	-0.82254	1.65E-05	8.10E-05
ATP2A3	1.519908	2.516911	0.727671	1.95E-14	3.86E-13
TCAP	1.209047	0.500242	-1.27317	8.61E-07	5.91E-06
FAM169A	0.38825	0.201216	-0.94824	1.78E-07	1.43E-06
GPLD1	0.444143	0.289705	-0.61644	1.64E-06	1.05E-05
HLA-DOA	7.406687	16.93376	1.193002	9.50E-20	2.42E-18
NKX6-3	0.211515	0.104988	-1.01054	0.001339	0.003792
SVEP1	0.519495	1.027804	0.984384	1.76E-07	1.42E-06

Gene	conMean	treatMean	logFC	pValue	FDR
SIGLEC5	0.072201	0.197236	1.449833	2.73E-26	9.65E-25
SIRPB2	0.228671	0.804634	1.815058	8.92E-40	7.53E-38
SPP1	119.4698	310.6508	1.378648	9.21E-25	3.02E-23
IGFL1	0.851083	2.14	1.330239	5.81E-05	0.000245
GAL3ST1	2.2312	0.896392	-1.31562	1.32E-08	1.32E-07
MYLK2	0.535487	0.291152	-0.87908	0.001859	0.005044
C1QB	59.65439	204.5137	1.777497	1.42E-44	2.19E-42
LAIR2	0.060227	0.184083	1.611879	2.80E-21	7.67E-20
CYP27C1	0.130967	0.084059	-0.63973	0.019171	0.037395
KLHDC7B	1.18286	2.819591	1.253206	8.40E-23	2.50E-21
LINC00514	0.220073	0.126621	-0.79747	0.000417	0.001386
FCRL5	0.057631	0.226641	1.97548	1.28E-19	3.24E-18
LY9	0.052155	0.218446	2.06639	2.55E-42	2.89E-40
ARID3C	0.859864	0.34725	-1.30813	5.19E-07	3.73E-06
CD1C	0.217245	1.069496	2.299538	9.96E-27	3.62E-25
CELF3	0.187032	0.02308	-3.0186	9.92E-07	6.71E-06
RHOH	0.128087	0.477547	1.898514	2.33E-49	1.10E-46
ZAP70	0.255099	0.813904	1.673801	2.12E-35	1.33E-33
IQCA1	4.09414	2.689002	-0.60649	4.86E-08	4.33E-07
RGS20	0.384	0.183863	-1.06247	0.00251	0.00656
MSX1	20.53187	7.868059	-1.38379	2.46E-05	0.000115
SERPINA1	12.64574	30.08198	1.250248	6.68E-28	2.62E-26
BPIFA2	0.306285	0.154315	-0.98899	2.71E-06	1.63E-05
TTPA	0.230589	0.390873	0.761376	0.000884	0.002656
MICB	2.969867	5.165959	0.798638	1.88E-12	3.10E-11
NEK10	0.146282	0.083419	-0.8103	6.06E-05	0.000255
SOX3	1.133562	0.114306	-3.30989	5.18E-09	5.64E-08
GALNT17	7.45893	4.570689	-0.70656	0.000682	0.002117
STAP1	0.122137	0.350639	1.521488	3.83E-22	1.10E-20
MNS1	3.634549	2.36759	-0.61836	3.83E-07	2.84E-06
SMIM6	1.457625	0.578679	-1.33278	0.000251	0.000889
PLCB4	2.526095	1.464808	-0.7862	4.02E-09	4.48E-08
VCAM1	2.123293	6.139369	1.531787	5.32E-22	1.52E-20
GPR87	0.369069	0.613558	0.733311	0.000587	0.001864
GPR141	0.458008	1.093892	1.256025	3.48E-25	1.18E-23
SLC6A13	1.72373	0.727654	-1.24421	7.78E-07	5.41E-06
PGR	0.985429	0.644415	-0.61276	0.012	0.025196
CFAP61	0.338268	0.146469	-1.20757	2.27E-07	1.78E-06
PRRT2	1.274115	0.804368	-0.66357	0.002426	0.006372
CAMK2B	0.631707	0.356982	-0.8234	1.27E-08	1.27E-07
CD1D	0.433486	0.807841	0.898085	6.69E-25	2.21E-23
ARC	1.64176	1.065345	-0.62392	0.000369	0.001245
GNA15	1.447738	3.890132	1.426019	3.04E-43	3.99E-41
GAD1	0.325422	0.199081	-0.70896	1.85E-05	8.95E-05
APOBEC3A	0.44543	0.996489	1.161655	4.56E-14	8.82E-13

Gene	conMean	treatMean	logFC	pValue	FDR
CFAP161	0.753149	0.323342	-1.21987	0.0002	0.000731
FAM155B	2.393058	1.209999	-0.98385	4.40E-08	3.96E-07
NME8	0.220533	0.345043	0.645784	3.19E-13	5.68E-12
SCN5A	0.722023	0.399193	-0.85496	2.01E-09	2.34E-08
DRC3	1.260414	0.775655	-0.70041	5.14E-10	6.50E-09
CCL22	0.193594	0.624258	1.689108	5.74E-31	2.68E-29
GCSAM	0.172589	0.263447	0.610173	7.20E-10	8.91E-09
LOX	5.231193	10.94669	1.065283	6.36E-10	7.91E-09
GPX3	102.1739	240.1868	1.23313	2.16E-09	2.51E-08
LGALS1	204.9425	314.2719	0.616795	1.72E-14	3.42E-13
GLP1R	0.23545	0.114492	-1.04018	5.95E-05	0.000251
NKG7	3.322437	15.95923	2.264077	2.98E-46	7.38E-44
AQP9	3.24685	5.354984	0.721842	6.70E-09	7.14E-08
HCK	2.2858	6.559244	1.52083	3.28E-45	5.74E-43
NLRP3	0.33149	0.869948	1.391966	2.14E-35	1.34E-33
SCGB3A1	16.92556	4.297188	-1.97774	3.57E-05	0.00016
CHST11	2.964851	4.737432	0.676146	2.17E-17	5.06E-16
PPARGC1A	0.286326	0.113903	-1.32986	0.001738	0.004756
KRTAP5-1	0.423535	0.255035	-0.73178	1.71E-05	8.39E-05
MYOG	0.442305	0.094852	-2.2213	0.000457	0.0015
XIRP1	0.046201	0.18151	1.974054	1.63E-11	2.43E-10
ZNF366	0.144677	0.219869	0.603815	4.35E-07	3.18E-06
PRR29	1.485316	0.913178	-0.7018	0.001637	0.004516
IL6	0.957876	1.912865	0.997825	3.79E-08	3.46E-07
HELT	0.173758	0.026979	-2.68719	0.000757	0.002325
ADGRG2	5.92417	3.779148	-0.64855	0.006936	0.015709
STEAP4	0.588482	1.024875	0.800378	3.15E-09	3.56E-08
ACTC1	1.300845	0.143421	-3.18112	0.01053	0.022483
MS4A8	1.423702	0.512908	-1.47288	0.002688	0.006966
GPR84	0.600722	2.324743	1.952301	4.27E-45	7.38E-43
PILRA	4.273242	7.654246	0.840929	1.56E-23	4.80E-22
KCNRG	0.585071	0.30874	-0.92222	4.21E-07	3.09E-06
SMIM2	0.140646	0.065468	-1.10322	0.020292	0.03913
HNMT	3.277053	5.322472	0.699697	3.11E-20	8.13E-19
SGSM1	0.437645	0.287162	-0.6079	1.38E-06	8.98E-06
CD3D	0.824261	4.328164	2.392582	4.17E-47	1.21E-44
MAPK8IP1	26.17284	13.15353	-0.99262	5.08E-14	9.76E-13
MILR1	1.631202	3.777325	1.211429	4.38E-33	2.30E-31
GLI1	0.594839	0.229122	-1.37638	5.32E-08	4.70E-07
SLCO2B1	1.986669	5.759345	1.535553	1.02E-30	4.68E-29
SAMSN1	0.973528	3.091836	1.667169	7.76E-50	3.93E-47
ADAMTS19	0.214774	0.054345	-1.9826	1.52E-09	1.80E-08
CCR4	0.103582	0.405294	1.968196	5.43E-36	3.56E-34
HMOX1	8.71964	17.78931	1.02867	4.49E-29	1.86E-27
NEXN	1.435007	2.333508	0.701443	8.85E-09	9.20E-08
DAND5	0.940311	0.253307	-1.89225	0.025496	0.04749

Gene	conMean	treatMean	logFC	pValue	FDR
SIT1	0.695824	2.668296	1.939125	3.94E-43	5.08E-41
PLN	0.707365	1.347095	0.929325	3.68E-11	5.26E-10
MUC5AC	0.254581	0.078181	-1.70324	8.64E-06	4.59E-05
ARRDC5	0.086537	0.22407	1.372568	1.23E-24	4.01E-23
IL1B	1.579187	4.019721	1.347914	1.03E-25	3.57E-24
BTK	0.481862	1.562573	1.697233	1.04E-49	5.08E-47
CLUL1	2.137374	1.287857	-0.73087	2.06E-06	1.28E-05
METTL24	2.67075	1.609809	-0.73036	0.001581	0.004381
KLHDC8A	7.243874	4.794124	-0.59549	1.96E-07	1.56E-06
KCNJ4	0.534089	0.264556	-1.01351	4.34E-05	0.00019
FAM161A	1.965303	1.302828	-0.5931	1.17E-09	1.39E-08
AMBP	1.446593	0.140738	-3.36158	0.006056	0.013993
JPH4	1.041687	0.433388	-1.26519	1.08E-06	7.20E-06
UPK2	6.163724	2.763045	-1.15754	0.001734	0.004745
CCL4L2	2.127146	5.297673	1.316439	1.04E-24	3.42E-23
CENPV	5.649983	3.026423	-0.90063	2.71E-05	0.000126
CFAP69	0.786987	0.499588	-0.6556	2.21E-08	2.12E-07
GJA5	2.779807	5.347337	0.943836	3.56E-08	3.26E-07
GRIN2C	0.322617	0.193288	-0.73907	0.001011	0.002984
LIN28A	0.192965	0.049335	-1.96766	0.000905	0.002708
HSPA6	1.617176	2.455097	0.602303	2.33E-08	2.22E-07
SASH3	1.861496	6.532201	1.811106	5.54E-53	9.82E-50
TUBB8B	0.328544	0.168279	-0.96523	1.85E-06	1.16E-05
ZNF804A	0.088239	0.150659	0.771792	1.62E-19	4.07E-18
GBP4	4.441136	13.87477	1.643463	1.42E-29	6.05E-28
ASGR2	0.060044	0.180814	1.59042	2.64E-34	1.55E-32
ANKDD1B	0.19301	0.097994	-0.9779	7.03E-06	3.83E-05
LRRK2	0.156972	0.370099	1.237398	6.40E-24	2.02E-22
PTGFR	0.096236	0.174627	0.859636	5.49E-10	6.89E-09
DNAAF3	2.514293	1.594257	-0.65727	1.61E-06	1.03E-05
TAS1R1	0.319631	0.210316	-0.60385	6.79E-06	3.70E-05
PLAC8	0.388115	0.728758	0.908955	4.05E-16	8.89E-15
TDO2	1.011345	2.461241	1.28311	4.66E-14	9.00E-13
WIF1	1.255051	0.240575	-2.38319	0.008247	0.018228
HABP2	0.310267	0.062352	-2.31501	0.002666	0.006918
ACP5	10.3651	17.94472	0.791826	1.75E-16	3.90E-15
SLC28A3	1.718148	3.215371	0.904131	7.01E-12	1.09E-10
SNX10	4.09392	6.718964	0.714756	4.36E-22	1.25E-20
ZNF711	2.03655	1.136019	-0.84214	6.47E-11	9.04E-10
MUC6	1.925904	0.758637	-1.34405	0.000211	0.000764
GABRE	2.878655	5.436673	0.917329	6.13E-09	6.58E-08
IL2RB	1.134478	5.022469	2.146368	1.93E-45	3.69E-43
LILRA6	0.210562	0.481868	1.194396	5.40E-25	1.81E-23
COL9A2	21.48385	10.42724	-1.0429	5.17E-05	0.000222
PRDM8	0.170622	0.318986	0.902693	3.01E-20	7.88E-19

Gene	conMean	treatMean	logFC	pValue	FDR
ANKLE1	2.162064	1.151188	-0.90929	1.18E-07	9.83E-07
TNFRSF17	0.240674	0.89595	1.896335	2.26E-14	4.44E-13
VNN3	0.212623	0.519236	1.288094	1.24E-11	1.88E-10
FRRS1L	0.608838	0.316986	-0.94164	1.13E-07	9.46E-07
MEF2C	1.002253	1.774276	0.823984	1.28E-23	3.96E-22
MARCHF11	0.358265	0.096983	-1.88522	8.76E-09	9.14E-08
CORO2B	4.283603	2.41233	-0.8284	3.25E-05	0.000147
AC005041.1	6.636183	4.336437	-0.61384	4.95E-11	6.98E-10
OIT3	0.156843	0.092882	-0.75585	0.003868	0.009518
UBD	1.343837	6.964636	2.37369	9.29E-28	3.58E-26
COL11A2	1.136825	0.645698	-0.81608	0.01108	0.023494
MYOZ3	0.219699	0.11004	-0.9975	0.000235	0.000839
PCDHB11	2.904069	1.483144	-0.96942	3.57E-05	0.00016
OTOS	0.488551	0.106601	-2.19628	1.83E-05	8.87E-05
EDN3	3.77358	0.561853	-2.74767	7.81E-08	6.72E-07
CCL17	0.285068	1.17484	2.043085	6.01E-21	1.62E-19
TMEM156	0.116662	0.395547	1.761515	9.26E-39	7.21E-37
GPR174	0.066475	0.28736	2.111983	3.83E-33	2.02E-31
NCR3	0.130487	0.568435	2.123094	1.54E-38	1.17E-36
EZHIP	0.381277	0.110026	-1.793	0.000368	0.001241
ATRNL1	0.296865	0.189719	-0.64595	0.000119	0.000461
FEZF2	0.154116	0.416821	1.435409	0.002233	0.005919
SBK3	0.23234	0.114887	-1.01602	1.14E-06	7.56E-06
PRKCG	1.418627	0.540842	-1.39122	0.003801	0.009368
SNX31	0.296909	0.481781	0.698359	0.000207	0.00075
CCDC187	0.52155	0.31459	-0.72934	3.44E-06	2.02E-05
MARCHF4	0.169005	0.04446	-1.92649	0.000317	0.001094
NPW	21.90456	13.7928	-0.66732	0.001021	0.00301
HLA-DOB	1.490474	2.648674	0.829499	2.41E-12	3.93E-11
FLI1	0.69187	1.377894	0.993892	1.29E-33	7.24E-32
SLC9A4	1.504285	0.908227	-0.72795	0.001622	0.004478
IL32	15.07656	31.01188	1.040513	4.15E-20	1.07E-18
DNAH9	0.238674	0.11257	-1.08422	1.29E-05	6.51E-05
TNNC2	1.384393	0.855498	-0.69442	0.000755	0.002319
CXCR1	0.060632	0.16306	1.42726	9.41E-05	0.000375
CATSPERD	0.308368	0.108804	-1.50293	0.00673	0.015307
CYTH4	1.017597	2.95306	1.537045	8.23E-43	9.88E-41
IL5RA	0.414248	0.172219	-1.26625	0.001202	0.003457
COL10A1	5.43317	12.22417	1.16987	4.43E-12	7.09E-11
NPHS1	0.861446	0.172964	-2.31629	0.003052	0.007774
CST11	0.174769	0.116506	-0.58505	0.004736	0.011344
GIMAP8	0.620874	1.493204	1.266038	1.28E-30	5.84E-29
AIF1	7.361899	23.09317	1.649317	3.76E-53	7.61E-50
FAS	3.992714	6.3312	0.665109	3.09E-16	6.83E-15
PSTPIP1	0.660095	1.206823	0.870468	1.84E-26	6.58E-25
TIMP3	2.609338	3.977649	0.608232	0.005054	0.011982

Gene	conMean	treatMean	logFC	pValue	FDR
SIAH3	0.998181	0.556447	-0.84306	8.43E-05	0.000341
APOL1	25.0065	50.41269	1.011484	3.07E-18	7.41E-17
TNFSF14	0.894515	1.51061	0.755954	7.20E-14	1.37E-12
TDRD12	1.051475	0.323093	-1.70239	0.000183	0.000675
TP53TG3D	0.429221	0.226677	-0.92108	5.42E-05	0.000231
DCAF12L1	1.516648	0.827259	-0.87448	3.45E-06	2.02E-05
CCR2	0.169341	0.830149	2.29344	3.85E-38	2.90E-36
PTGIS	5.327165	10.33241	0.955738	1.18E-06	7.80E-06
BRSK2	0.600409	0.218171	-1.46048	7.50E-08	6.49E-07
CLIC2	1.276108	2.424132	0.925718	1.16E-26	4.18E-25
PDLIM3	2.23599	4.501275	1.00942	2.09E-10	2.74E-09
HAMP	0.36725	0.967183	1.397026	9.13E-23	2.71E-21
SYP	0.926478	0.505413	-0.87429	0.004387	0.010615
GNB4	2.206101	3.685202	0.740245	1.01E-20	2.70E-19
GSTA3	0.344977	0.146737	-1.23327	0.001817	0.004943
PRR36	15.80539	9.480348	-0.7374	8.61E-06	4.57E-05
LBX2	0.561734	0.286598	-0.97086	0.019855	0.038459
CCL26	0.223888	0.453096	1.017037	1.12E-10	1.51E-09
TM4SF4	0.658378	0.241213	-1.44861	0.005869	0.013615
APOC1	16.94357	46.43472	1.454466	5.41E-32	2.66E-30
PLEKHG4B	3.992978	2.211857	-0.85221	1.48E-12	2.50E-11
MDFIC	2.270483	3.600104	0.665039	8.77E-15	1.77E-13
TOGARAM2	0.159504	0.08384	-0.92788	1.37E-05	6.89E-05
DMKN	24.44581	14.38092	-0.76543	1.10E-08	1.11E-07
ARSI	0.547267	0.825558	0.593123	8.69E-06	4.60E-05
PPM1E	0.289342	0.129196	-1.16321	1.08E-05	5.59E-05
NUGGC	0.179093	0.369604	1.045273	1.86E-15	3.91E-14
MYCN	11.55003	7.038302	-0.7146	0.004839	0.011555
FAM171A2	18.75453	10.05954	-0.89868	4.89E-08	4.35E-07
MT1G	76.15013	138.3855	0.861775	5.76E-05	0.000243
FILIP1	2.710601	1.346097	-1.00983	1.91E-05	9.21E-05
HLA-E	112.0912	203.6155	0.861174	3.46E-30	1.53E-28
IL4I1	25.46635	46.38793	0.865157	1.20E-12	2.02E-11
GNGT1	1.229254	0.795994	-0.62695	5.01E-07	3.62E-06
USH1C	0.436684	0.143635	-1.60419	7.69E-05	0.000314
LST1	2.226571	7.536521	1.759075	4.91E-51	3.66E-48
MEX3A	18.32754	10.25831	-0.83722	4.99E-12	7.95E-11
EPHA6	0.455052	0.284952	-0.67531	0.018839	0.036829
ADGRG3	0.278681	0.426844	0.615093	2.32E-07	1.81E-06
LILRB5	0.235717	0.415198	0.816744	6.01E-13	1.04E-11
TMEM272	0.128194	0.074333	-0.78626	2.89E-05	0.000133
NFAM1	0.686834	1.978089	1.526074	3.51E-39	2.83E-37
DNALI1	12.08001	7.037065	-0.77958	1.79E-06	1.13E-05
ADRA2B	1.804984	1.126711	-0.67987	0.000168	0.000627
GAS2	0.628191	0.181798	-1.78886	4.83E-09	5.31E-08

Gene	conMean	treatMean	logFC	pValue	FDR
SERPINB8	2.05776	3.224499	0.648	7.10E-14	1.35E-12
VGF	1.892532	0.171245	-3.46619	2.52E-05	0.000118
FOXP4	56.14222	33.03169	-0.76524	9.33E-07	6.35E-06
SNAI2	4.255725	7.068636	0.732027	1.24E-07	1.03E-06
CLC	0.120473	0.246942	1.035458	3.95E-05	0.000175
IDO1	5.06496	12.33198	1.283782	1.92E-18	4.67E-17
NLGN3	1.388994	0.848117	-0.7117	0.005802	0.013486
RETREG1	2.654927	1.666348	-0.67198	3.72E-07	2.77E-06
KRTAP5-8	0.179037	0.111876	-0.67836	0.00033	0.00113
PAG1	0.724266	1.153712	0.671692	6.16E-17	1.41E-15
PARP15	0.137356	0.36863	1.42425	2.05E-29	8.69E-28
MCTP1	0.189187	0.377983	0.998509	7.33E-28	2.86E-26
SIRPA	7.834282	13.4571	0.780495	5.34E-17	1.23E-15
PIWIL1	0.343658	0.207354	-0.72888	0.000498	0.001616
FGFR1	18.81564	11.72805	-0.68197	9.88E-09	1.01E-07
LILRA5	0.299622	0.780485	1.381228	2.50E-30	1.12E-28
IL17RD	5.166221	2.745805	-0.91188	1.55E-05	7.70E-05
C1QA	45.27682	150.1373	1.729438	1.10E-45	2.30E-43
GBP5	0.959065	3.630007	1.920272	2.55E-36	1.71E-34
INPP4B	0.379408	0.778976	1.037829	7.18E-15	1.45E-13
CSF2RA	0.836659	2.394979	1.517301	4.98E-40	4.25E-38
SUCNR1	0.35809	0.948172	1.404826	1.13E-25	3.89E-24
KCNN3	1.116239	0.740677	-0.59173	0.000449	0.001474
CD2	1.365192	7.179632	2.394806	2.65E-50	1.56E-47
MS4A4E	0.088327	0.20775	1.233923	8.31E-16	1.79E-14
TPPP	2.037989	1.292713	-0.65674	3.06E-06	1.82E-05
LCN8	0.379156	0.098081	-1.95074	0.000127	0.000487
TEX46	0.169821	0.108433	-0.64721	2.15E-07	1.69E-06
TMEM145	0.962656	0.128615	-2.90396	8.09E-07	5.61E-06
DEUP1	0.604216	0.328023	-0.88127	0.002661	0.006907
CD72	0.838608	1.573831	0.908212	2.78E-25	9.50E-24
SEMA6C	5.535501	2.810466	-0.9779	3.47E-06	2.03E-05
ARHGEF38	0.178551	0.099502	-0.84354	0.001801	0.004908
CLEC18B	1.749147	0.761755	-1.19925	0.000105	0.000412
H2BW1	0.376047	0.152928	-1.29806	4.79E-06	2.71E-05
TLR10	0.091341	0.303556	1.732623	2.16E-28	8.55E-27
DCDC1	0.147652	0.088418	-0.73978	3.05E-07	2.32E-06
C19orf67	0.283495	0.184112	-0.62274	0.002989	0.007628
SLC6A3	0.168756	0.069621	-1.27734	0.002883	0.007397
DEFB126	2.220986	1.178581	-0.91415	0.000463	0.001515
PIH1D3	0.258837	0.094773	-1.4495	0.000427	0.001412
DEPP1	13.82092	22.7856	0.721269	9.50E-14	1.78E-12
NANOS1	2.145324	1.152219	-0.89678	1.18E-07	9.83E-07
CLEC12A	0.254437	0.678376	1.414775	5.72E-19	1.41E-17
NGB	0.686933	0.355156	-0.95172	0.00701	0.015854
CIDEC	0.185454	0.643348	1.794535	0.00011	0.000432

Gene	conMean	treatMean	logFC	pValue	FDR
LRRC75A	1.34844	0.880862	-0.6143	1.65E-08	1.62E-07
NEK5	0.977683	0.580697	-0.75158	1.07E-07	9.00E-07
KLRB1	0.362834	1.415779	1.964213	7.01E-44	1.00E-41
GAL3ST3	7.860554	3.174763	-1.30798	2.77E-09	3.15E-08
ALX3	0.424874	0.167281	-1.34476	0.009433	0.020474
NRXN2	2.204451	1.397379	-0.6577	0.019658	0.038135
GALNT13	0.64283	0.346449	-0.89179	0.000563	0.001798
APOL3	1.734103	5.045965	1.540941	1.43E-33	7.95E-32
BMP6	2.473007	1.489781	-0.73117	0.01606	0.03221
PIEZO2	0.232368	0.355857	0.614889	0.000485	0.001577
RETN	0.164344	0.395109	1.265529	3.73E-12	6.01E-11
GDAP1	1.558161	0.912775	-0.77151	1.01E-05	5.26E-05
BEX1	16.78367	4.835798	-1.79523	1.72E-08	1.67E-07
CFB	16.19624	25.36874	0.647393	9.22E-10	1.11E-08
LRRC14B	0.534482	0.244321	-1.12937	9.95E-10	1.20E-08
B4GALNT4	15.04085	9.027772	-0.73644	1.67E-09	1.96E-08
CLDN6	95.31784	49.89068	-0.93398	0.00014	0.000534
SCGB1D2	105.0811	40.98469	-1.35835	0.00063	0.001979
C1QC	72.40289	235.8056	1.703479	3.24E-45	5.73E-43
SP140	0.22197	0.677353	1.609547	1.99E-37	1.41E-35
PRB4	0.155229	0.10325	-0.58826	0.001113	0.003234
RASGRF2	0.44179	0.694727	0.653086	8.67E-08	7.40E-07
CYBB	4.952377	18.66941	1.914483	5.74E-46	1.25E-43
ADCYAP1R1	3.101011	1.286714	-1.26905	3.71E-06	2.16E-05
MEGF10	0.192354	0.337259	0.810097	0.000988	0.002924
AP3B2	1.018406	0.654364	-0.63815	2.06E-05	9.85E-05
C10orf55	0.260568	0.528303	1.019707	9.43E-18	2.24E-16
OAS2	16.15442	25.02632	0.631517	1.07E-08	1.08E-07
CLECL1	0.183686	0.469127	1.352736	1.21E-29	5.19E-28
RGS7BP	0.27757	0.1545	-0.84524	0.000705	0.002183
PLEK	2.652255	8.724862	1.717913	2.33E-47	7.03E-45
MT1E	71.9049	110.1498	0.615305	2.28E-06	1.40E-05
CAMKV	0.234508	0.031595	-2.89187	6.78E-11	9.43E-10
SMO	16.93275	9.774994	-0.79265	2.55E-13	4.62E-12
PCDHB9	0.897335	0.547348	-0.71319	7.83E-05	0.000319
UBASH3A	0.142892	0.495637	1.79436	5.89E-39	4.66E-37
SERPINB5	1.250972	0.757633	-0.72348	8.42E-07	5.80E-06
CEP126	0.675397	0.390527	-0.79031	9.42E-07	6.40E-06
CCR1	2.471685	6.290572	1.347697	3.43E-39	2.78E-37
DRAM1	3.049578	5.603415	0.877697	9.26E-30	4.01E-28
ZNF683	0.346208	2.05893	2.572183	6.48E-41	6.12E-39
PDCD1	0.468493	1.418373	1.598139	1.91E-26	6.84E-25
GDAP1L1	0.249655	0.06423	-1.95861	6.58E-06	3.60E-05
KCNMB1	0.350751	0.611196	0.801188	2.13E-30	9.52E-29
RSPH4A	1.602929	0.730398	-1.13396	3.29E-07	2.48E-06

Gene	conMean	treatMean	logFC	pValue	FDR
ANK1	0.370756	0.16558	-1.16294	0.014377	0.029361
PEG10	27.47509	17.25596	-0.67103	2.03E-07	1.61E-06
TLR4	0.800563	2.196304	1.455991	1.59E-30	7.21E-29
FAM166C	0.950038	0.41135	-1.20762	2.84E-09	3.22E-08
SFMBT2	0.476206	0.918424	0.947573	2.75E-26	9.68E-25
SPATA46	0.142185	0.092367	-0.62232	0.000329	0.001129
CYYR1	12.24399	4.572956	-1.42087	0.000113	0.000441
C1orf189	1.82977	1.158865	-0.65895	0.008081	0.017921
BFSP1	0.587521	0.343262	-0.77533	0.004538	0.010938
GPR183	2.699143	8.887701	1.719309	6.03E-39	4.75E-37
PTGIR	0.4985	0.954146	0.936617	2.38E-17	5.55E-16
VEPH1	1.432974	0.944746	-0.60101	4.37E-05	0.000191
CXCL9	3.460244	25.3208	2.871377	3.06E-33	1.63E-31
CRIP3	0.615216	0.384447	-0.67831	0.002367	0.006225
CHIT1	0.297486	0.565926	0.927792	3.92E-06	2.27E-05
IRF4	0.200453	0.792665	1.983447	6.29E-29	2.56E-27
OLFML1	2.623354	4.001244	0.609036	4.19E-08	3.78E-07
JAML	0.180019	0.692298	1.943242	6.04E-43	7.64E-41
NELL2	1.227915	0.701117	-0.80848	0.003273	0.008253
SAMD9L	3.391382	6.025487	0.829204	3.30E-14	6.43E-13
MS4A6A	2.279699	7.839336	1.781888	1.75E-47	5.40E-45
CAPN6	7.39864	1.980066	-1.90171	0.000165	0.000619
WFDC1	0.549598	0.290444	-0.92011	0.001124	0.003258
P2RY8	0.262609	0.756442	1.526315	5.62E-31	2.63E-29
TNFRSF11A	0.324141	0.681918	1.072978	1.11E-26	4.03E-25
EMB	3.355338	5.114397	0.608106	2.43E-13	4.40E-12
FUT7	0.06585	0.199616	1.599977	2.25E-32	1.13E-30
ADAMTS9	3.249314	1.786452	-0.86304	0.000125	0.000482
SCARF1	1.029285	1.575932	0.614563	1.08E-17	2.56E-16
LY86	3.069224	8.399024	1.452348	6.15E-42	6.60E-40
IGFL3	0.077239	0.25643	1.731172	0.000222	0.000797
IGF2BP1	0.796698	0.227348	-1.80913	0.003756	0.009274
LAIR1	1.420903	4.581528	1.68902	4.26E-50	2.32E-47
TMEM163	0.814317	0.539377	-0.5943	0.01956	0.037981
VAT1L	0.244668	0.384538	0.652303	0.000119	0.00046
SCUBE1	0.411513	0.227326	-0.85617	0.009023	0.019736
SAMD15	1.341363	0.821741	-0.70694	3.06E-05	0.00014
TNFRSF18	1.543883	4.078455	1.40146	8.92E-16	1.92E-14
SAMD13	0.734153	0.466418	-0.65446	3.14E-06	1.87E-05
ASCL1	0.16295	0.066512	-1.29275	0.008193	0.018138
GALNT16	0.759159	0.461661	-0.71757	0.000371	0.001248
ESPNL	1.135809	0.610099	-0.8966	0.000776	0.002377
RSPH1	4.353212	2.391074	-0.86442	3.95E-05	0.000175
ENKUR	1.58721	0.75998	-1.06246	1.39E-06	9.01E-06
PTPN22	0.348401	1.16944	1.746997	6.39E-52	6.97E-49
DGKG	1.151486	0.589864	-0.96504	0.015375	0.031015

Gene	conMean	treatMean	logFC	pValue	FDR
STK36	8.765643	5.726621	-0.61418	7.92E-10	9.75E-09
FCMR	1.303488	2.444658	0.907255	1.01E-23	3.15E-22
PRAP1	2.722768	1.106561	-1.29899	7.01E-09	7.44E-08
ANKRD66	0.347201	0.100072	-1.79473	0.000782	0.002392
FCER1A	0.399606	1.394811	1.803421	4.17E-16	9.14E-15
MAB21L2	0.197065	0.299449	0.603641	1.43E-06	9.24E-06
C16orf74	1.051911	0.693935	-0.60014	0.022744	0.043072
TNFRSF9	0.220594	0.647815	1.55419	2.61E-30	1.16E-28
GASK1B	2.705793	4.119515	0.606423	2.65E-08	2.50E-07
C11orf88	0.915884	0.371048	-1.30356	0.007376	0.016569
TM6SF1	0.408907	0.871579	1.091858	3.22E-24	1.04E-22
CCR5	0.458109	1.990159	2.119122	9.99E-53	1.42E-49
ZNF559-ZNF177	0.274461	0.165031	-0.73386	5.20E-08	4.61E-07
HLA-B	238.4393	507.4848	1.089742	5.19E-25	1.74E-23
NTN3	0.681788	0.342283	-0.99413	1.36E-06	8.88E-06
TNFSF18	0.090686	0.199908	1.140383	8.78E-14	1.65E-12
TIGD1	4.784664	3.116699	-0.6184	7.38E-13	1.27E-11
PLP1	0.177926	0.07714	-1.20572	0.022174	0.042185
GPC2	1.322599	0.662404	-0.99759	1.74E-08	1.70E-07
SIRPB1	0.245749	0.54122	1.139029	2.17E-18	5.27E-17
CCDC181	0.996541	0.320232	-1.63781	2.60E-17	6.05E-16
FYB1	1.154746	4.226666	1.871944	3.32E-52	3.92E-49
CPA3	0.249513	0.716993	1.522848	3.92E-13	6.91E-12
CCDC113	2.74834	1.704489	-0.68922	5.77E-07	4.12E-06
PODXL2	30.8217	20.00914	-0.62329	2.43E-07	1.89E-06
PAK5	0.244157	0.112654	-1.11591	0.000321	0.001106
CCL13	0.421159	2.298707	2.448387	3.72E-18	8.93E-17
APOA1	122.278	64.23377	-0.92876	0.000611	0.001928
CD53	6.099012	21.55064	1.821083	7.11E-56	1.01E-51
GPR156	0.932651	0.593258	-0.65268	1.17E-07	9.74E-07
PRRT4	0.3882	0.104238	-1.89691	0.003317	0.00835
PIFO	5.78082	2.945605	-0.97271	1.57E-06	1.01E-05
FGF3	3.801299	0.522067	-2.86419	9.45E-05	0.000377
CD247	0.237225	0.926543	1.9656	3.60E-48	1.31E-45
FLACC1	0.309693	0.143855	-1.10622	3.52E-06	2.06E-05
CLEC2B	2.05422	4.417626	1.104681	6.45E-27	2.39E-25
CPLX2	3.029023	1.366831	-1.14802	0.002002	0.005378
NLGN1	0.326926	0.186044	-0.81332	0.001666	0.004583
VWA3A	1.807672	0.95876	-0.91489	3.26E-09	3.68E-08
KRTDAP	2.19645	0.625356	-1.81242	3.18E-08	2.94E-07
RNFT2	2.134853	1.293419	-0.72295	1.54E-10	2.04E-09
EBI3	1.180407	3.660623	1.632805	1.49E-34	8.96E-33
FOXN1	0.785938	0.5227	-0.58843	0.022495	0.042697
SERPINB7	0.568418	1.2341	1.118434	3.45E-05	0.000156
SLC66A1L	0.651993	1.063165	0.705438	0.001011	0.002984

Gene	conMean	treatMean	logFC	pValue	FDR
CYP2W1	1.700859	0.607416	-1.48551	1.85E-05	8.95E-05
C5AR2	0.126285	0.19331	0.61423	7.72E-16	1.66E-14
GAL3ST4	3.085488	6.705705	1.11989	2.07E-27	7.87E-26
PPP1R32	1.924094	1.155143	-0.73611	5.47E-05	0.000232
DUSP19	1.329728	0.849955	-0.64567	9.34E-11	1.28E-09
CAMK4	0.114756	0.182625	0.670307	8.65E-10	1.05E-08
NPPC	0.693741	0.357373	-0.95696	0.00023	0.000823
ANPEP	10.59441	19.11872	0.851683	2.66E-11	3.87E-10
FZD2	16.55265	10.77215	-0.61976	0.008999	0.019688
SLC2A5	0.850786	1.899069	1.158425	7.80E-28	3.02E-26
SLC25A27	2.5818	1.608468	-0.68269	4.25E-06	2.44E-05
MYO15A	0.267006	0.142583	-0.90507	4.37E-05	0.000191
FASLG	0.113914	0.640526	2.491307	7.22E-45	1.18E-42
SIGLEC1	1.886067	5.206685	1.464984	9.57E-27	3.50E-25
GABRG2	0.170169	0.05114	-1.73446	0.00019	0.000697
TMEM176B	19.5368	39.53061	1.016776	3.09E-20	8.07E-19
SDK2	2.309532	0.881283	-1.38992	1.94E-11	2.84E-10
GSDMA	0.250613	0.41772	0.737073	1.28E-13	2.38E-12
AFAP1L2	9.845156	6.229831	-0.66022	0.013331	0.027563
THBS1	16.15375	29.32981	0.860499	1.05E-10	1.43E-09
DHRS9	0.265248	0.614099	1.211129	1.13E-11	1.71E-10
KYNU	0.157149	0.367986	1.227515	3.71E-32	1.85E-30
KIAA0513	0.469439	0.713984	0.604953	1.63E-11	2.42E-10
IGLL5	6.909335	20.84348	1.592977	3.34E-21	9.11E-20
COL2A1	4.061582	0.546085	-2.89485	3.06E-15	6.32E-14
TCAF2	0.243677	0.376777	0.628739	8.23E-09	8.63E-08
ADCY7	0.913902	1.453432	0.669353	6.56E-09	6.99E-08
CASS4	0.410496	0.703432	0.777042	3.31E-22	9.62E-21
HES6	12.07886	5.934796	-1.02521	1.16E-05	5.93E-05
RASGRP4	0.520366	0.823332	0.661948	1.24E-19	3.14E-18
DDN	0.177883	0.090896	-0.96864	6.58E-07	4.64E-06
SIRPG	0.243814	1.145422	2.232023	1.26E-43	1.75E-41
SV2C	0.570491	0.221236	-1.36662	7.24E-11	1.00E-09
GRIK5	8.096094	5.133468	-0.65729	0.000205	0.000744
TEKT1	1.47906	0.560533	-1.39981	0.000153	0.000577
SCTR	0.187154	0.056166	-1.73644	0.006005	0.013891
EOMES	0.144808	0.538758	1.895495	2.46E-33	1.34E-31
STMND1	0.470294	0.166288	-1.49988	7.42E-09	7.84E-08
KMO	0.16529	0.309574	0.905286	2.26E-27	8.58E-26
CCL18	1.466245	6.106165	2.05814	5.50E-13	9.60E-12
GSDMC	0.731265	1.418103	0.955496	2.09E-14	4.14E-13
KRT15	0.971796	1.885207	0.955997	0.000673	0.002093
CFAP45	3.634255	1.764877	-1.04209	3.15E-06	1.87E-05
CBX2	6.814552	3.327508	-1.03418	2.67E-09	3.05E-08
LRRC23	6.377362	4.022977	-0.6647	1.48E-07	1.21E-06
CD40	6.685364	11.07592	0.728348	9.36E-14	1.76E-12

Gene	conMean	treatMean	logFC	pValue	FDR
ERFE	5.167656	2.244216	-1.2033	0.012127	0.025407
DYDC1	0.317935	0.170213	-0.90139	5.92E-05	0.000249
DLGAP3	2.653496	1.234494	-1.10398	4.75E-05	0.000206
SGK1	5.673904	8.65615	0.609384	8.42E-12	1.30E-10
C2orf72	0.582822	0.348448	-0.74211	2.79E-05	0.000129
SELENOV	0.742603	0.173806	-2.09511	4.97E-06	2.81E-05
CST1	16.58688	9.192117	-0.85157	0.02394	0.044983
PCDHGA12	0.418116	0.72015	0.784394	1.70E-06	1.08E-05
PHOX2A	11.45806	7.249487	-0.66041	0.01265	0.026347
CXCR2	0.101241	0.318403	1.653053	6.04E-15	1.23E-13
TMEM232	0.195859	0.117013	-0.74315	0.000273	0.000958
CXCR6	0.268582	1.22412	2.188313	5.66E-46	1.25E-43
PECAM1	4.688566	8.331005	0.829344	2.50E-23	7.61E-22
LEFTY2	1.425013	0.455325	-1.64601	8.20E-07	5.67E-06
DCN	17.35628	33.87438	0.964737	1.46E-12	2.47E-11
SIGLEC12	0.11265	0.385113	1.773429	1.05E-11	1.60E-10
ITM2C	118.8687	63.35122	-0.90792	1.23E-06	8.15E-06
TRPS1	4.583485	7.683592	0.745336	3.55E-09	3.98E-08
FXYD7	4.762746	1.796044	-1.40697	6.48E-09	6.94E-08
SSTR3	0.286466	0.136604	-1.06837	0.001087	0.003173
CXCR3	0.66846	2.918788	2.126457	3.74E-44	5.59E-42
DPT	1.218558	1.862897	0.612373	1.55E-07	1.26E-06
VAX2	5.830926	3.4179	-0.77061	2.26E-08	2.16E-07
GDF10	1.508849	0.188321	-3.00218	6.59E-08	5.77E-07
TACC1	3.306549	5.021538	0.602803	8.10E-12	1.25E-10
LINGO1	12.47759	8.053899	-0.63158	6.56E-05	0.000274
FABP7	0.193178	0.028259	-2.77314	2.81E-05	0.00013
CD274	0.487772	1.117379	1.195839	5.79E-23	1.74E-21
NOD2	0.564785	1.161997	1.040832	2.42E-25	8.34E-24
CLDN19	1.55622	0.348206	-2.16003	1.34E-06	8.78E-06
NRAP	0.273099	0.107174	-1.34947	6.40E-05	0.000267
C2orf73	0.370306	0.162983	-1.184	5.33E-06	2.99E-05
GBP1	13.19256	27.87808	1.079406	7.87E-22	2.23E-20
SMIM10L2A	2.108534	1.301588	-0.69597	2.61E-07	2.01E-06
ARHGDIB	23.02697	51.30898	1.155887	2.12E-45	3.90E-43
C20orf85	20.24492	5.736238	-1.81938	0.000417	0.001385
TLR1	0.954697	2.159494	1.177579	1.01E-30	4.65E-29
TFEC	0.281967	1.044497	1.889212	5.71E-49	2.61E-46
IGLL1	0.107496	0.365162	1.764255	5.46E-05	0.000232
NPC1L1	0.224524	0.122614	-0.87275	6.16E-05	0.000258
RSPO4	5.390432	3.404324	-0.66303	0.001981	0.005324
TFPI2	8.501946	18.74594	1.140713	9.08E-08	7.72E-07
ANKRD13B	3.715483	2.079741	-0.83715	2.24E-09	2.59E-08
PCSK9	0.561338	0.255229	-1.13708	0.009349	0.020323
RDH12	0.271929	0.155638	-0.80503	4.08E-06	2.35E-05

Gene	conMean	treatMean	logFC	pValue	FDR
MMP24	3.738821	2.344715	-0.67317	0.023478	0.04422
TMPRSS2	0.736788	0.414901	-0.82848	0.000912	0.002725
CD37	2.641654	7.697198	1.542892	1.47E-50	9.06E-48
RIMKLA	0.992356	0.553797	-0.8415	0.00032	0.001102
CCDC178	0.246796	0.143072	-0.78658	0.001169	0.003371
ADAP2	2.07397	4.812431	1.214371	7.59E-38	5.55E-36
LRRC55	1.976657	3.514348	0.830195	4.04E-06	2.33E-05
GABRQ	0.52211	0.155779	-1.74486	0.00567	0.013222
SLC12A8	4.057398	6.115307	0.59187	1.26E-08	1.26E-07
GRIN2A	1.225193	0.658898	-0.89488	0.002224	0.005898
TRO	4.884353	2.74332	-0.83224	6.52E-09	6.96E-08
LPXN	3.05298	6.318678	1.049405	7.23E-43	8.91E-41
MPL	0.355706	0.237673	-0.58171	1.25E-06	8.24E-06
BATF3	0.427934	0.640401	0.581586	4.41E-17	1.02E-15
ENPP3	0.952234	0.382098	-1.31738	0.001285	0.003662
MROH2A	0.451023	0.281718	-0.67895	0.000201	0.000735
PCDH8	0.468719	0.033468	-3.80789	6.12E-05	0.000257
GRB14	1.172869	0.62427	-0.9098	0.000409	0.001363
ADGRV1	0.173714	0.080592	-1.10801	2.91E-07	2.21E-06
PODN	2.891743	5.145358	0.831332	1.29E-08	1.28E-07
IHH	2.028553	0.165311	-3.61719	2.08E-06	1.29E-05
RIPPLY1	0.656132	0.076566	-3.09921	0.009145	0.01997
CCL3	2.218007	4.978961	1.166581	8.76E-24	2.74E-22
PSMB8	38.32048	65.80746	0.780136	1.45E-17	3.40E-16
BCL2A1	1.650325	4.939747	1.581687	4.25E-35	2.62E-33
CXCL13	1.371813	8.430766	2.61958	3.93E-27	1.48E-25
NCKAP5	0.395084	0.256489	-0.62326	2.95E-06	1.76E-05
MARCHF10	0.287363	0.148153	-0.95578	1.70E-06	1.08E-05
LILRB1	0.634143	2.027593	1.676889	6.32E-44	9.13E-42
EVPLL	0.209666	0.136947	-0.61448	0.007922	0.017617
RND2	1.848806	0.99284	-0.89696	2.89E-10	3.74E-09
TNIP3	0.136602	0.487124	1.834309	1.41E-29	6.04E-28
SCUBE3	2.320298	1.282584	-0.85526	0.001035	0.003045
CLEC18A	0.240533	0.110982	-1.11591	0.000263	0.000926
HCLS1	3.229264	6.187041	0.938044	2.73E-29	1.14E-27
KLHL13	0.764172	0.335868	-1.186	0.000212	0.000768
HLA-DPA1	31.4225	91.89776	1.548232	2.35E-36	1.59E-34
TBX1	2.013928	0.659141	-1.61135	7.86E-06	4.23E-05
CRTAC1	17.94128	10.64856	-0.75262	0.009426	0.020461
PRF1	0.660871	2.679287	2.019409	9.60E-43	1.14E-40
SEMA3D	0.425271	0.706774	0.732866	0.002356	0.006203
MYL7	0.434943	0.207176	-1.06997	2.54E-07	1.97E-06
GZMK	0.308694	1.641872	2.411091	1.80E-42	2.08E-40
UBE2L6	58.53392	91.62386	0.646451	8.03E-15	1.62E-13
FCGR1B	0.152295	0.467313	1.617519	1.09E-43	1.53E-41
CLEC7A	1.013798	3.155853	1.63826	6.30E-46	1.35E-43

Gene	conMean	treatMean	logFC	pValue	FDR
G0S2	7.594759	12.1944	0.683142	5.12E-12	8.13E-11
GFAP	1.194952	0.688495	-0.79544	1.54E-05	7.64E-05
ADAMTS18	0.278913	0.151714	-0.87846	0.02672	0.049367
C11orf53	0.237547	0.122072	-0.96048	0.003152	0.007986
THSD7B	0.420614	0.250944	-0.74513	0.000128	0.000492
DIO3	0.273346	0.534152	0.966522	5.17E-07	3.72E-06
CCDC169	0.207965	0.114099	-0.86605	2.62E-07	2.02E-06
CCDC39	0.476957	0.273908	-0.80017	0.00026	0.000915
TUBA4B	2.113369	0.648768	-1.70377	0.013613	0.028035
GDF11	6.996197	4.090731	-0.77421	5.46E-08	4.81E-07
CASQ1	0.811795	0.355628	-1.19075	7.65E-06	4.13E-05
TNFSF12	6.436536	9.840029	0.612378	2.47E-22	7.22E-21
MEST	64.57797	30.10167	-1.1012	2.86E-09	3.24E-08
GPM6A	0.769843	0.328382	-1.22919	0.000364	0.00123
PRDM1	1.348236	2.672242	0.986978	7.59E-25	2.51E-23
PCDHA1	0.230377	0.11191	-1.04166	3.23E-07	2.44E-06
PNMA8C	0.194092	0.061362	-1.66133	2.09E-13	3.82E-12
IBSP	0.853861	2.33934	1.454029	3.66E-09	4.09E-08
FOXN4	1.378542	0.37204	-1.88961	9.22E-11	1.26E-09
FCGR2A	3.700573	10.35572	1.484607	6.20E-43	7.77E-41
SAXO2	0.971857	0.549654	-0.82222	6.04E-06	3.34E-05
STAB1	3.597249	8.283129	1.203282	8.36E-26	2.91E-24
OR2L13	0.348893	0.144164	-1.27507	0.000163	0.000611
CD80	0.13659	0.422856	1.63032	5.19E-34	2.95E-32
ZBTB8B	0.192332	0.095402	-1.0115	9.20E-09	9.50E-08
CSF2RB	0.584281	2.034749	1.800117	1.52E-40	1.38E-38
JCHAIN	12.08754	69.37788	2.520955	3.16E-25	1.08E-23
TGM5	0.083931	0.130084	0.632162	0.000729	0.002247
AC136428.1	0.117566	0.351432	1.579771	1.15E-12	1.95E-11
FGF9	1.749026	0.744337	-1.23252	1.49E-09	1.76E-08
GZMH	0.529947	2.654864	2.324717	1.65E-42	1.91E-40
IL33	1.137033	1.818361	0.677365	2.30E-05	0.000108
KAZALD1	5.760256	2.5492	-1.17609	3.44E-10	4.43E-09
OR2I1P	0.368067	2.685741	2.867281	1.71E-35	1.09E-33
TCP11	0.643053	0.388781	-0.72598	7.12E-05	0.000294
FFAR4	0.214619	0.425002	0.985692	1.08E-23	3.35E-22
NLRC3	0.465914	0.726542	0.640982	3.79E-10	4.86E-09
RGS18	0.229465	0.744714	1.698414	4.56E-40	3.94E-38
TPSAB1	1.84048	3.259137	0.824408	2.50E-12	4.07E-11
MRO	0.144596	0.235778	0.705398	2.16E-13	3.95E-12
MAP3K15	0.188214	0.100731	-0.90187	7.59E-06	4.10E-05
DACH1	6.011568	2.334909	-1.36437	3.12E-08	2.88E-07
PTPRE	0.994224	1.50854	0.601511	3.34E-16	7.36E-15
SERPINA3	0.110008	0.300307	1.448837	0.001413	0.003969
INSYN1	0.703414	0.325236	-1.11289	0.017074	0.03392

Gene	conMean	treatMean	logFC	pValue	FDR
RD3	0.306049	0.131803	-1.21537	0.000155	0.000583
CHRNB2	0.354334	0.063985	-2.46931	0.000371	0.001248
CLVS1	0.3332	0.148766	-1.16334	1.71E-05	8.37E-05
EMP3	11.48588	21.68559	0.916875	3.67E-31	1.73E-29
C19orf38	1.137628	1.710566	0.588445	1.06E-15	2.27E-14
DACH2	0.172	0.11337	-0.60136	6.11E-06	3.37E-05
GREM2	0.134343	0.203469	0.59888	0.000698	0.002163
PLAAT4	49.30285	89.8563	0.865949	7.03E-15	1.42E-13
GNB3	0.611841	0.334525	-0.87104	0.006849	0.015541
CIB3	0.070814	0.182073	1.362417	0.002668	0.006924
ZEB2	0.573302	1.250302	1.12491	5.91E-30	2.59E-28
SOSTDC1	7.116607	3.139457	-1.18067	7.16E-08	6.23E-07
TNNC1	8.938103	5.049287	-0.82389	0.000215	0.000774
GDPD2	2.326473	1.232815	-0.91619	0.002316	0.006111
ARHGEF4	1.662216	1.100507	-0.59494	2.13E-06	1.32E-05
ABCG8	0.354392	0.176943	-1.00206	3.66E-05	0.000164
OMD	0.681614	1.997222	1.550969	1.45E-08	1.43E-07
ATOH7	0.335834	0.180925	-0.89235	0.000812	0.00247
ZNF483	0.184961	0.119049	-0.63566	2.32E-07	1.81E-06
ROPN1L	4.399085	1.750792	-1.3292	4.77E-05	0.000206
TNNT2	1.567772	0.676703	-1.21212	2.54E-05	0.000118
PEAK3	0.172347	0.363562	1.076881	6.52E-27	2.41E-25
FBP1	6.234814	11.65581	0.902631	1.50E-13	2.79E-12
KLRC1	0.050501	0.252231	2.320375	1.53E-29	6.48E-28
AQP6	0.826937	0.544817	-0.60201	0.021949	0.041818
SAA4	0.201874	0.367325	0.863604	2.50E-06	1.52E-05
ZNF729	0.176883	0.085454	-1.04957	3.30E-07	2.48E-06
WIPF1	2.824976	5.545081	0.97297	1.84E-33	1.01E-31
PIK3AP1	1.928005	3.819789	0.986384	1.01E-30	4.65E-29
FCN1	0.413403	1.524166	1.882401	7.82E-27	2.86E-25
ASTL	0.905564	0.476237	-0.92714	5.83E-07	4.15E-06
LGALS2	1.278363	2.07563	0.699252	4.54E-13	7.96E-12
FCER1G	37.44779	116.2286	1.634012	9.56E-53	1.42E-49
EFNB3	6.663331	4.1642	-0.6782	1.78E-07	1.43E-06
DCAF12L2	0.815293	0.428376	-0.92844	2.11E-07	1.66E-06
TREM1	0.595603	1.362637	1.193978	2.79E-15	5.80E-14
USP6	0.19308	0.117635	-0.71488	2.11E-07	1.67E-06
PTPN7	0.607406	1.633967	1.427645	1.16E-46	3.05E-44
RIPK3	2.042112	3.281862	0.684453	6.07E-12	9.56E-11
SPSB4	1.072538	0.32171	-1.7372	0.000432	0.001426
CA10	0.200436	0.025317	-2.98498	0.004195	0.010204
LRRC46	3.046104	1.580326	-0.94674	0.000668	0.002081
SAA2-SAA4	0.32938	0.711458	1.111025	8.53E-10	1.04E-08
ZNF98	0.748643	0.48964	-0.61256	1.49E-06	9.60E-06
SLC38A8	0.565609	0.242644	-1.22097	2.03E-07	1.61E-06
NME9	0.480782	0.270295	-0.83085	3.71E-08	3.39E-07

Gene	conMean	treatMean	logFC	pValue	FDR
NXNL1	0.184399	0.03735	-2.30366	1.78E-07	1.43E-06
SKIDA1	0.781452	0.471834	-0.72788	0.003128	0.007932
TNNI1	1.778796	0.687717	-1.37101	2.16E-12	3.54E-11
IGLON5	4.801715	2.127198	-1.1746	0.012902	0.026812
CLEC4A	0.732355	2.498577	1.770492	5.28E-48	1.87E-45
DPF1	1.183126	0.537585	-1.13804	4.13E-05	0.000182
DYNLRB2	0.756916	0.429545	-0.81732	0.000791	0.00241
IL2RG	2.968277	12.33192	2.0547	9.92E-51	6.39E-48
CPB2	0.170489	0.095946	-0.82938	5.93E-07	4.22E-06
RASL11B	2.812986	1.77647	-0.66309	3.16E-05	0.000144
CCDC30	0.751761	0.473785	-0.66604	3.55E-09	3.98E-08
IL24	0.329907	0.523854	0.667107	3.21E-13	5.72E-12
TMEM88	4.732714	2.989703	-0.66267	0.004118	0.010034
DOCK2	0.390288	1.236344	1.663471	4.09E-42	4.46E-40
KLHL6	0.347193	0.62296	0.843403	8.69E-22	2.45E-20
ONECUT2	0.497209	0.160809	-1.62851	0.000169	0.000629
SLAMF8	1.578326	6.672732	2.079883	7.63E-49	3.28E-46
GJB2	6.04685	15.20369	1.330165	3.15E-13	5.62E-12
PLA2G2D	0.219918	1.30361	2.567475	2.19E-29	9.19E-28
AC003002.2	0.120343	0.080304	-0.5836	0.001557	0.004324
ICAM1	17.66027	31.68258	0.843182	4.31E-16	9.41E-15
CD79B	0.693573	1.205483	0.797491	3.06E-13	5.47E-12
CD27	1.159399	3.263425	1.493009	1.99E-33	1.09E-31
MYH7B	1.003735	0.491224	-1.03093	1.10E-05	5.67E-05
CRMP1	4.621143	2.743223	-0.75238	2.61E-05	0.000122
FAM78A	0.754204	1.707724	1.179047	5.30E-36	3.50E-34
HHATL	0.182705	0.038082	-2.26234	1.43E-05	7.17E-05
MDH1B	2.019287	1.321605	-0.61155	2.12E-08	2.04E-07
IGSF5	0.139349	0.074735	-0.89885	1.31E-06	8.62E-06
SLC28A2	0.462331	0.300774	-0.62025	4.41E-05	0.000193
SMTNL1	0.965626	1.579318	0.709765	0.017915	0.035289
RPL3L	0.291228	0.183487	-0.66647	2.38E-05	0.000112
WDR35	3.073981	1.977992	-0.63607	1.51E-12	2.54E-11
GUCY1A1	1.086536	2.035525	0.905665	2.77E-11	4.01E-10
DLEC1	0.656504	0.353656	-0.89246	0.00017	0.000633
EYA1	0.78414	0.281823	-1.47632	0.006298	0.014452
LAPTM5	28.40656	95.91171	1.755483	5.34E-51	3.79E-48
COL3A1	164.3945	308.2926	0.907138	6.00E-09	6.45E-08
FAM177B	0.192911	0.364876	0.919471	2.38E-13	4.32E-12
RARRES1	22.48904	43.48828	0.951405	8.62E-11	1.18E-09
PPP1R18	8.358263	13.72886	0.715937	2.06E-21	5.69E-20
CD300LB	0.123998	0.293276	1.24194	1.02E-20	2.74E-19
SLC13A4	0.645344	0.347725	-0.89212	3.66E-05	0.000164
DUOX1	1.577578	0.885468	-0.8332	0.009764	0.021057
ZNF20	0.40951	0.272416	-0.58809	3.55E-06	2.07E-05

Gene	conMean	treatMean	logFC	pValue	FDR
RNF103-CHMP3	0.144985	0.094957	-0.61055	2.93E-06	1.75E-05
APOH	0.295016	0.058072	-2.34488	0.01236	0.025819
CSAG3	0.568282	0.951844	0.744119	3.84E-05	0.00017
ADCY8	2.796589	0.77018	-1.8604	2.96E-07	2.25E-06
EPHA7	0.419856	0.145567	-1.52821	9.58E-11	1.31E-09
CCRL2	0.412819	1.052995	1.350917	2.26E-45	4.11E-43
PDIA2	2.675838	0.869223	-1.62219	5.63E-06	3.14E-05
CPNE4	0.859793	0.44406	-0.95323	0.000344	0.001172
TNFRSF14	4.110684	6.954695	0.758609	1.39E-16	3.10E-15
CCNO	10.41114	5.938257	-0.81002	0.011624	0.024512
REEP1	0.954561	0.59176	-0.68983	9.60E-06	5.03E-05
SNORC	2.309638	1.511469	-0.61171	6.23E-05	0.000261
SPOCD1	0.143196	0.299736	1.065696	8.28E-12	1.28E-10
CTSG	0.065804	0.19435	1.562401	2.05E-06	1.28E-05
ZNF219	12.00631	7.396418	-0.69889	8.70E-10	1.06E-08
LRRC71	0.70625	0.236236	-1.57995	1.02E-06	6.85E-06
FGR	1.001183	2.874997	1.521855	2.38E-43	3.21E-41
SPTB	0.392182	0.193648	-1.01809	0.000202	0.000735
VDR	3.297019	5.546428	0.750397	7.90E-14	1.49E-12
CD93	3.178409	5.100738	0.682401	2.55E-12	4.15E-11
RAC2	5.818171	15.81117	1.442307	6.09E-47	1.69E-44
ZNF423	4.154062	1.619031	-1.35939	2.32E-09	2.67E-08
NT5E	4.369367	7.080791	0.696486	7.62E-09	8.02E-08
GSTM3	6.13847	3.813964	-0.68659	0.000946	0.002813
INPP5D	1.643537	3.504334	1.092336	7.41E-28	2.88E-26
SLC37A2	0.831499	2.201253	1.404538	1.85E-34	1.11E-32
FMNL1	3.456847	6.240697	0.852251	1.44E-27	5.52E-26
NCF1	0.505794	1.7815	1.81647	2.63E-48	9.81E-46
PHF21B	0.286614	0.045493	-2.65539	8.72E-08	7.44E-07
IRF1	8.123837	15.73792	0.954012	1.16E-24	3.79E-23
ZNF727	0.595685	0.372249	-0.67828	0.000131	0.000503
IFNG	0.064269	0.335378	2.383593	2.47E-31	1.18E-29
PRG4	0.499668	0.801338	0.681441	0.019463	0.037854
SPNS3	0.20392	0.328838	0.68937	2.57E-07	1.99E-06
PLA2G2A	2.066155	6.240198	1.594643	9.93E-07	6.71E-06
OLR1	1.688243	5.80734	1.782355	8.99E-38	6.53E-36
BICC1	1.301419	2.10463	0.693481	8.28E-07	5.72E-06
CAPSL	5.245109	1.710936	-1.61619	8.68E-06	4.60E-05
GFY	0.193865	0.079097	-1.29337	0.000783	0.002393
ARRB1	1.771411	2.702603	0.609451	2.22E-14	4.38E-13
ASCL5	0.429136	0.209121	-1.0371	1.65E-12	2.75E-11
GPR31	0.099928	0.157314	0.654691	1.98E-09	2.31E-08
CYSLTR1	0.195891	0.765176	1.965741	3.43E-36	2.27E-34
H2BW2	0.152227	0.051421	-1.56578	4.79E-09	5.26E-08
ELFN2	0.208172	0.121113	-0.78142	0.023192	0.043754
ASIC1	1.135649	0.723016	-0.65142	0.018651	0.036512

Gene	conMean	treatMean	logFC	pValue	FDR
ADGRE1	0.25265	0.589126	1.221437	3.66E-25	1.24E-23
LILRA4	0.053626	0.216005	2.010063	1.44E-29	6.12E-28
ADAMTS2	2.52632	4.949156	0.970145	6.03E-08	5.30E-07
MYH6	0.443827	0.09315	-2.25237	3.05E-08	2.83E-07
TEX15	0.157005	0.065528	-1.26064	0.000183	0.000676
BATF2	2.651888	4.393525	0.728359	3.93E-13	6.91E-12
KY	0.212545	0.103099	-1.04374	5.28E-07	3.79E-06
CXorf21	0.435146	1.280125	1.556714	7.73E-41	7.25E-39
CDH22	1.185961	0.775671	-0.61254	0.018675	0.036552
TEKT3	0.23148	0.15356	-0.59209	1.09E-05	5.64E-05
XKR7	0.476476	0.11696	-2.02639	0.007885	0.017551
LIN28B	0.672484	0.306703	-1.13266	0.001981	0.005324
RASL10B	3.811384	2.319263	-0.71665	0.016184	0.032407
MALL	0.474712	0.803896	0.759958	0.000205	0.000746
SCT	1.140149	2.31253	1.02025	1.49E-09	1.76E-08
FAM163A	0.233372	0.1154	-1.01598	0.000185	0.000682
ELAVL3	0.359297	0.074968	-2.26082	5.32E-12	8.44E-11
SMCO2	0.152372	0.279659	0.876073	3.02E-10	3.89E-09
TIMD4	0.203752	0.807362	1.986404	7.81E-25	2.57E-23
NKPD1	0.272059	0.169084	-0.68618	2.56E-06	1.55E-05
DUSP10	3.857645	6.57423	0.769102	2.08E-11	3.04E-10
TNNT3	1.53457	0.672549	-1.19012	5.04E-07	3.63E-06
PNMT	2.874923	0.743732	-1.95067	4.66E-09	5.13E-08
CRYGC	2.532213	0.816	-1.63376	0.015213	0.030759
CFAP157	2.682117	1.6757	-0.67861	9.21E-05	0.000368
NOVA1	0.836228	0.462051	-0.85584	1.89E-06	1.19E-05
IFFO1	0.761366	1.58357	1.056519	2.38E-29	9.99E-28
NCAM1	3.758856	2.121717	-0.82506	8.30E-05	0.000336
CRYGB	1.993004	0.478011	-2.05983	3.76E-05	0.000167
CALN1	0.089041	0.201319	1.176944	0.010248	0.021966
RIIAD1	0.421326	0.195548	-1.10742	0.00142	0.003986
GPR68	0.544773	1.144776	1.071339	1.29E-21	3.62E-20
TNFAIP3	6.835699	12.1166	0.825824	4.11E-17	9.51E-16
HLA-DMB	9.642335	20.53784	1.09083	2.65E-25	9.06E-24
HTR1D	0.137743	0.068857	-1.00031	0.000141	0.000537
KLHL38	0.147165	0.499132	1.761982	4.44E-08	4.00E-07
MAFA	0.659584	0.299307	-1.13993	4.36E-06	2.50E-05
SOX11	3.032395	0.582965	-2.37898	6.65E-05	0.000277
CSRNP3	0.829931	0.489368	-0.76207	6.03E-14	1.15E-12
FAM216B	1.465634	0.449775	-1.70425	0.014646	0.029834
TEX11	0.094206	0.160327	0.767119	2.70E-08	2.55E-07
QPCT	4.440627	7.507423	0.757554	1.36E-07	1.12E-06
SELE	0.075338	0.207326	1.460449	1.64E-11	2.43E-10
GAS2L2	0.611088	0.302985	-1.01213	1.59E-07	1.29E-06
EVA1C	6.510749	10.8114	0.731658	6.11E-12	9.61E-11

Gene	conMean	treatMean	logFC	pValue	FDR
LRRC31	0.384686	0.104319	-1.88269	0.00086	0.002593
DRD5	0.134331	0.077974	-0.78473	0.001808	0.00492
SLIT3	4.519206	2.938422	-0.62103	0.000196	0.000717
DHRS2	1.643098	0.293532	-2.48483	0.000354	0.001201
MYCBPAP	0.310155	0.201049	-0.62544	0.000269	0.000946
CATIP	0.487581	0.28895	-0.75482	0.000748	0.002299
COL11A1	4.347525	10.44793	1.264951	2.24E-07	1.76E-06
GPR83	0.282161	0.095467	-1.56344	1.19E-05	6.09E-05
C5orf49	4.320941	2.228755	-0.95511	9.60E-06	5.03E-05
TCF7L1	11.61413	4.753126	-1.28893	1.94E-10	2.55E-09
SIM2	1.34949	0.607176	-1.15223	9.81E-06	5.12E-05
PDCL2	2.415149	1.524508	-0.66377	0.001358	0.003834
FCGR1A	0.998424	3.137733	1.651998	1.41E-47	4.44E-45
MSTN	0.254105	0.040544	-2.64787	4.59E-05	0.000199
AGXT	0.195678	0.011625	-4.07318	3.22E-05	0.000146
SLC10A4	0.755868	0.345393	-1.1299	0.00014	0.000534
ODC1	89.57852	58.25621	-0.62074	3.00E-08	2.79E-07
ACSM3	3.807018	2.277142	-0.74144	1.84E-08	1.79E-07
CTNNA2	1.557545	0.616074	-1.3381	6.38E-08	5.60E-07
SLC26A10	0.291012	0.160898	-0.85493	0.001285	0.003662
CETP	0.312781	0.56687	0.857865	2.61E-13	4.70E-12
IRX6	0.727537	0.486486	-0.58062	0.010895	0.023139
LRATD1	4.352689	1.745444	-1.31831	1.62E-05	7.98E-05
PAX2	10.54625	3.431807	-1.61969	1.38E-06	8.98E-06
BANK1	0.687127	1.08571	0.65999	1.04E-06	6.96E-06
PCDHB10	1.863964	1.13128	-0.72042	0.000888	0.002666
TMPRSS6	1.456289	0.958507	-0.60344	0.010292	0.022038
HMGA2	5.92819	1.781458	-1.73453	1.00E-08	1.02E-07
DPYSL5	1.602619	0.105986	-3.91849	8.69E-12	1.34E-10
MSI1	14.97468	6.611998	-1.17937	6.07E-12	9.56E-11
IGSF21	0.540303	1.439892	1.414122	2.07E-26	7.37E-25
GABRP	2.255442	4.041438	0.841458	2.57E-06	1.56E-05
FDCSP	0.243257	1.632287	2.74634	7.70E-14	1.46E-12
ATP4B	0.229149	0.048703	-2.23421	2.05E-11	2.99E-10
FPR2	0.08538	0.274171	1.683111	3.76E-22	1.09E-20
SLC16A12	0.301338	0.049466	-2.60687	5.06E-08	4.49E-07
EMX2	66.48357	38.31044	-0.79526	1.73E-10	2.29E-09
COL24A1	0.172576	0.302614	0.810251	0.001236	0.00354
P2RX1	0.174174	0.357603	1.037829	7.51E-10	9.26E-09
CXXC4	0.607054	0.285936	-1.08613	0.005574	0.013031
OTULINL	2.265441	3.775593	0.736911	5.90E-16	1.28E-14
TRAF3IP3	0.146432	0.497233	1.763692	1.33E-45	2.62E-43
CYP17A1	2.119979	0.925484	-1.19577	0.000915	0.002734
ITGB2	5.2799	17.08722	1.694335	2.69E-45	4.82E-43
TOX	3.890422	2.048659	-0.92525	0.002727	0.00706
CTSW	1.26498	5.2636	2.056936	1.79E-40	1.62E-38

Gene	conMean	treatMean	logFC	pValue	FDR
SAMD5	0.49113	0.248124	-0.98505	0.000161	0.000605
CA8	3.846653	2.309445	-0.73606	4.06E-05	0.000179
CD96	0.324218	1.129664	1.800856	4.19E-42	4.54E-40
PDCD1LG2	0.491489	1.848783	1.911345	2.45E-42	2.80E-40
MT3	1.197085	0.770999	-0.63473	0.020004	0.038689
GPR34	1.994524	5.696313	1.513984	4.20E-32	2.08E-30
CLCN4	1.797418	1.140514	-0.65624	5.86E-07	4.17E-06
ADCY1	0.459315	0.283048	-0.69844	1.60E-06	1.02E-05
CLEC3B	0.845594	1.600919	0.920863	0.001139	0.003295
ST8SIA6	0.238358	0.45842	0.943541	2.08E-05	9.92E-05
CEL	10.87696	1.593821	-2.77071	0.000208	0.000753
SLC22A17	6.833736	4.562155	-0.58296	0.000164	0.000615
SELL	1.410472	3.884734	1.461638	5.20E-31	2.44E-29
ZNF285	0.987021	0.643521	-0.61709	3.07E-07	2.33E-06
LHFPL3	0.24529	0.080135	-1.61399	0.000209	0.000759
GALNT5	0.330913	0.650172	0.974368	3.23E-07	2.44E-06
EFEMP1	6.44557	11.01022	0.772463	2.88E-07	2.20E-06
ITGA4	0.474	1.102576	1.217919	1.71E-24	5.54E-23
NPL	1.376976	2.948199	1.098331	1.22E-35	7.83E-34
SLC49A3	2.039119	3.18274	0.642323	1.19E-11	1.79E-10
CLMP	1.486517	2.496119	0.747751	3.87E-08	3.52E-07
CROCC2	0.423392	0.21772	-0.95952	4.86E-09	5.32E-08
RGS1	6.71441	19.87665	1.565742	6.16E-29	2.52E-27
ZNF730	0.732001	0.420197	-0.80078	3.70E-09	4.13E-08
NR5A1	3.306138	1.518047	-1.12293	2.11E-06	1.31E-05
NR0B1	0.517852	0.250745	-1.04632	0.021968	0.041836
ANKRD22	0.725066	2.450937	1.75715	3.25E-38	2.46E-36
CD209	0.777697	1.485456	0.933625	7.13E-13	1.23E-11
ADGRE2	0.232969	0.682162	1.549977	7.63E-32	3.74E-30
CLEC18C	0.23051	0.118921	-0.95483	0.000238	0.000849
DLX2	0.764605	0.166049	-2.20311	0.006124	0.014116
SLAMF7	0.763755	3.223171	2.077298	1.49E-35	9.51E-34
MSC	2.03952	3.865141	0.922292	1.25E-15	2.65E-14
DPY19L2	0.546581	0.29363	-0.89644	2.67E-06	1.61E-05
ZNF286A	1.245638	0.820202	-0.60283	9.17E-14	1.72E-12
TMEM89	0.150109	0.085493	-0.81214	5.77E-06	3.21E-05
SPARC	205.7082	335.6258	0.706254	2.28E-06	1.40E-05
TAP1	16.44725	35.3552	1.104076	1.39E-23	4.30E-22
SOD2	7.744653	16.00334	1.047101	1.25E-22	3.70E-21
CSF1	15.78145	31.39227	0.99218	5.86E-19	1.44E-17
CD200R1	0.394806	0.686487	0.798088	1.04E-17	2.46E-16
CYTL1	5.125967	1.820089	-1.49382	0.000391	0.00131
MAP6	2.030295	1.166684	-0.79928	0.000436	0.001439
TBX4	0.206853	0.086772	-1.25331	1.36E-05	6.82E-05
TBX2	11.66552	6.398726	-0.86639	1.62E-05	8.00E-05

Gene	conMean	treatMean	logFC	pValue	FDR
TMEM221	4.548723	2.159695	-1.07463	1.63E-09	1.92E-08
CAPS	26.75147	16.94495	-0.65876	0.008107	0.017969
LCP2	1.358989	4.47015	1.717789	1.05E-54	3.71E-51
MORN5	3.693431	1.081293	-1.7722	7.25E-10	8.95E-09
FPR3	3.165884	11.05601	1.80415	2.16E-40	1.92E-38
COL26A1	7.587029	3.703627	-1.0346	0.001601	0.004432
CD180	0.487201	1.414678	1.537886	4.33E-41	4.20E-39
C1orf87	0.351903	0.151264	-1.2181	0.008192	0.018138
TUBB2B	12.6	1.661661	-2.92273	1.55E-09	1.83E-08
TMEFF1	0.944602	0.489661	-0.94792	5.00E-09	5.46E-08
CALY	0.434956	0.111872	-1.95902	5.24E-06	2.95E-05
C5orf46	0.444236	1.331887	1.584075	1.78E-11	2.62E-10
SULT1C4	2.27275	0.962514	-1.23956	2.21E-09	2.55E-08
ITGA5	6.056127	10.37018	0.775974	2.35E-13	4.26E-12
WDR38	5.140418	2.065174	-1.31562	0.004755	0.011378
TRPM5	0.781158	0.461583	-0.75902	1.67E-06	1.06E-05
BPIFB1	0.544819	0.325505	-0.7431	0.000522	0.001679
GREM1	0.559068	0.879922	0.654352	5.33E-10	6.71E-09
DNAAF4	0.939058	0.620224	-0.59843	1.18E-08	1.19E-07
HPN	28.82032	15.12826	-0.92984	0.001001	0.00296
SMIM24	6.072789	2.20165	-1.46377	2.98E-05	0.000137
IMPG2	3.994636	1.386544	-1.52657	3.42E-06	2.01E-05
DAPP1	1.296645	2.388026	0.881036	7.36E-19	1.80E-17
MPEG1	2.496268	7.658024	1.617199	1.55E-37	1.10E-35
PIK3R5	0.497988	1.269358	1.349915	3.74E-33	1.99E-31
IL15RA	2.405255	5.380965	1.161675	1.08E-28	4.38E-27
ESRRB	0.253704	0.093005	-1.44777	0.002483	0.006497
MT1H	12.83878	27.34278	1.090652	7.15E-05	0.000295
FAM181A	6.149256	4.076887	-0.59294	6.77E-05	0.000281
PLEKHD1	0.19724	0.107674	-0.87328	5.15E-06	2.90E-05
PCDHGB1	0.920667	0.402463	-1.19382	0.000387	0.001297
GIMAP7	1.622771	4.651622	1.519274	2.33E-41	2.34E-39
DSCAML1	1.198	0.71797	-0.73863	0.008107	0.017969
ACKR2	1.662732	3.09093	0.894485	1.40E-07	1.15E-06
APOBEC2	0.410936	0.234308	-0.81051	0.000167	0.000624
NETO1	0.075897	0.166282	1.131521	5.31E-08	4.70E-07
FAM81B	1.959036	0.92131	-1.08839	0.000276	0.000967
SH2D1B	0.075338	0.21519	1.514157	4.40E-25	1.48E-23
KLF2	9.087485	14.63894	0.687858	1.85E-11	2.72E-10
OSCAR	1.531918	3.801093	1.311075	1.06E-38	8.21E-37
PCK1	3.348274	5.19584	0.633939	0.00228	0.006027
LHFPL1	0.324579	0.17912	-0.85765	1.30E-07	1.07E-06
VNN2	0.46842	1.307767	1.481233	1.35E-28	5.41E-27
COL1A2	139.0364	235.7702	0.761919	1.49E-06	9.60E-06
SLC5A12	0.356969	0.186638	-0.93556	1.60E-07	1.30E-06
DNAH6	0.411036	0.179204	-1.19766	1.62E-12	2.70E-11

Gene	conMean	treatMean	logFC	pValue	FDR
GABRR1	0.566294	0.214025	-1.40377	2.90E-05	0.000133
EPB41L3	0.636191	1.448994	1.18752	3.11E-32	1.55E-30
EGF	0.090098	0.182147	1.015546	0.007234	0.016305
FOXJ1	46.24073	28.19151	-0.7139	0.000394	0.001318
RASGRP3	0.419453	0.660784	0.655669	8.63E-19	2.11E-17
ARMC3	1.69138	0.898994	-0.91182	1.06E-05	5.48E-05
TREM2	7.673645	20.49394	1.417213	2.89E-31	1.38E-29
KCNJ13	0.367326	0.138134	-1.411	5.91E-06	3.27E-05
TAP2	4.323512	7.383876	0.772175	1.12E-16	2.52E-15
ZIC2	4.271161	2.499281	-0.77311	0.020694	0.03979
CCL11	1.697003	5.276292	1.636535	6.65E-22	1.89E-20
CNTN3	1.256234	0.714266	-0.81457	3.59E-05	0.000161
SIGLEC14	0.697136	2.451787	1.814322	9.41E-33	4.82E-31
NPIPB15	2.238972	1.148194	-0.96347	0.000218	0.000784
CD14	25.7339	71.90335	1.482389	2.71E-42	3.05E-40
TNFAIP8	1.396186	2.25991	0.694774	2.99E-15	6.20E-14
FBN2	2.322239	0.985196	-1.23703	0.004564	0.010991
ECRG4	9.709834	3.924632	-1.30689	2.31E-07	1.81E-06
TMEM158	7.525961	12.02214	0.675746	8.94E-08	7.61E-07
DKK4	4.602531	0.108533	-5.40622	0.025964	0.048203
RUFY4	0.420638	0.82299	0.968295	4.05E-07	2.99E-06
ZCCHC12	2.189587	0.606997	-1.8509	8.67E-08	7.40E-07
UNC79	0.355664	0.211587	-0.74926	0.0047	0.011267
WDR66	0.54889	0.313862	-0.80639	0.000965	0.002863
AC010325.1	0.402968	0.255159	-0.65927	0.002644	0.006874
GFI1	0.154404	0.4441	1.524177	3.34E-33	1.78E-31
LRRIQ1	0.981871	0.477236	-1.04083	7.17E-09	7.60E-08
PCDHA6	0.336945	0.220307	-0.613	0.003857	0.009495
GCGR	0.178779	0.046262	-1.95027	3.21E-07	2.43E-06
CCDC151	2.972618	1.315817	-1.17578	5.44E-11	7.63E-10
SLC44A5	1.412473	0.784529	-0.84832	5.23E-05	0.000224
GPBAR1	0.412102	0.625263	0.601462	2.27E-19	5.69E-18
AATK	1.610977	0.896233	-0.84599	0.014792	0.030053
GPR18	0.152543	0.425069	1.478482	1.40E-34	8.49E-33
OR7G2	0.052054	0.355871	2.773273	0.007501	0.0168
GNLY	0.988818	4.385549	2.14898	8.99E-29	3.64E-27
TBXAS1	0.89673	2.074717	1.21017	2.07E-34	1.23E-32
LRRC7	0.126575	0.073441	-0.78533	0.01147	0.02421
ARHGAP30	2.179778	4.643826	1.091132	2.69E-32	1.35E-30
APLP1	16.10029	9.036113	-0.83331	5.17E-05	0.000222
LILRB2	0.717724	2.329556	1.698553	1.24E-45	2.48E-43
CARD16	3.360503	5.747539	0.774267	2.10E-20	5.56E-19
ECM1	5.835438	11.00171	0.914814	1.66E-12	2.76E-11
MPO	0.154208	0.097241	-0.66524	0.00559	0.01306
APOBEC3D	1.756763	2.916656	0.731396	1.03E-13	1.93E-12

Gene	conMean	treatMean	logFC	pValue	FDR
KCNE4	0.981513	1.624576	0.726984	4.98E-09	5.45E-08
LRP2BP	0.617231	0.405986	-0.60438	0.000876	0.002634
EVI2B	2.965639	10.5126	1.825705	4.71E-51	3.66E-48
CRTAM	0.190358	0.535195	1.491349	5.19E-33	2.72E-31
LCN2	152.8127	312.6142	1.032619	2.86E-08	2.67E-07
CRLF1	3.136423	0.8087	-1.95544	2.35E-07	1.83E-06
RSAD2	6.319434	9.765263	0.627863	9.51E-09	9.76E-08
STOML3	0.442093	0.206286	-1.0997	3.36E-06	1.98E-05
EYA4	2.239723	1.079203	-1.05335	4.71E-06	2.67E-05
CCNB1IP1	22.08391	14.24674	-0.63236	6.03E-09	6.48E-08
DPYD	1.15929	2.773291	1.258357	1.13E-31	5.53E-30
CNMD	0.455577	0.056701	-3.00625	2.47E-05	0.000115
CD36	0.670604	1.294021	0.948328	1.66E-07	1.34E-06
GIMAP2	1.731343	3.572371	1.044991	2.97E-30	1.32E-28
VSIG4	7.502651	22.25988	1.568974	2.67E-33	1.44E-31
JAK2	1.165468	1.818419	0.641775	5.73E-17	1.31E-15
IGF2	499.1142	306.0784	-0.70547	0.004551	0.010968
PLEKHF1	10.79394	16.56107	0.617574	1.08E-05	5.59E-05
GABBR1	3.596635	2.383144	-0.59378	0.003302	0.008315
CSPG5	3.139305	2.051803	-0.61355	0.000421	0.001395
SPAG17	0.678267	0.438746	-0.62847	0.000779	0.002382
TAGAP	0.612713	2.024077	1.72398	4.97E-47	1.41E-44
C3orf80	0.716286	1.338887	0.902428	6.82E-15	1.38E-13
TFF3	19.37541	5.497983	-1.81725	0.001463	0.004095
RTP4	12.52086	19.99129	0.675038	4.22E-10	5.37E-09
EXOC3L4	0.439858	0.709078	0.688906	9.93E-07	6.71E-06
NNMT	19.05742	42.06915	1.14241	1.45E-18	3.53E-17
LCP1	11.98115	22.37889	0.901371	4.68E-24	1.49E-22
GNG2	0.918112	1.58926	0.791612	5.14E-27	1.92E-25
CPED1	0.66033	0.988251	0.581691	7.36E-14	1.40E-12
GPR37L1	0.786459	0.489316	-0.6846	0.00537	0.012617
ZNF443	3.261855	2.170413	-0.58772	0.000435	0.001435
FCAR	0.090424	0.279281	1.626931	4.79E-20	1.23E-18
TSPAN7	12.57886	8.265182	-0.60588	3.18E-06	1.89E-05
NKAIN4	3.741489	0.817336	-2.19461	2.91E-09	3.30E-08
SPINK1	12.42822	0.270934	-5.51954	0.002359	0.006208
PRRX1	2.743212	4.313499	0.652993	5.20E-10	6.56E-09
TMPRSS9	0.481968	0.215222	-1.16311	0.001779	0.004855
NWD1	0.759308	0.311557	-1.28519	0.000116	0.000451
RGS22	0.515573	0.247151	-1.06078	3.48E-05	0.000157
CD84	0.646445	2.169294	1.746626	1.45E-42	1.70E-40
PAQR6	2.513132	1.587091	-0.6631	0.001018	0.003002
C6orf118	1.013996	0.424477	-1.25629	2.45E-09	2.82E-08
PTH2R	4.488319	2.701813	-0.73225	1.67E-06	1.06E-05
P2RY12	0.154766	0.456483	1.560475	2.05E-21	5.65E-20
LSP1	4.693619	11.69524	1.317148	9.84E-42	1.03E-39

Gene	conMean	treatMean	logFC	pValue	FDR
TGFB1	15.69954	25.83417	0.718559	1.29E-20	3.43E-19
CD8A	0.804653	3.388725	2.074303	5.31E-37	3.66E-35
GRM6	0.176009	0.103003	-0.77296	4.54E-08	4.07E-07
DOCK10	0.465464	1.045172	1.167	1.03E-33	5.79E-32
BIN2	0.743907	2.460104	1.725525	3.92E-51	3.27E-48
NR0B2	0.361644	0.045954	-2.9763	4.58E-06	2.60E-05
RASSF4	1.841681	3.2456	0.817462	6.78E-23	2.03E-21
PIH1D2	1.501036	0.935697	-0.68184	3.02E-07	2.30E-06
ITGAM	0.964525	2.885584	1.580973	1.26E-37	9.06E-36
ATP7B	3.631143	2.422697	-0.58381	4.66E-05	0.000202
MMP20	0.071764	0.129169	0.847936	0.007357	0.016539
HAND1	0.163663	0.348212	1.08924	0.003343	0.0084
IL22RA2	0.056898	0.151561	1.413437	1.54E-14	3.07E-13
C2CD6	0.333788	0.20892	-0.67598	0.00013	0.000498
ITGAL	0.769218	2.921306	1.92515	4.88E-45	8.13E-43
LCK	0.845995	2.209862	1.385234	1.58E-32	8.00E-31
CCL24	0.225967	0.628287	1.475312	1.68E-11	2.49E-10
SLFN12L	0.088486	0.228868	1.370997	7.29E-29	2.96E-27
STAT4	0.237136	0.502639	1.08381	1.28E-37	9.13E-36
DCDC2B	0.604233	0.403538	-0.5824	9.39E-05	0.000375
SCN3B	0.594805	0.378317	-0.65282	0.016518	0.03295
TTYH1	5.416259	2.489429	-1.12148	0.000247	0.000875
PITPNC1	4.593978	3.034542	-0.59827	1.35E-05	6.78E-05
GMFG	2.775053	7.174143	1.370291	4.06E-47	1.20E-44
IL7	0.269283	0.500155	0.893254	5.18E-22	1.48E-20
GCK	1.258672	0.512694	-1.29573	2.28E-08	2.18E-07
TTLL2	0.167375	0.077897	-1.10345	5.92E-06	3.28E-05
WDFY4	0.237073	0.716034	1.594696	4.34E-33	2.28E-31
ATP6V0D2	0.109363	0.187004	0.773944	0.000215	0.000777
ICOS	0.12344	0.631123	2.354114	5.70E-40	4.84E-38
B2M	319.9477	673.3362	1.073491	2.35E-34	1.39E-32
ATP6V1FNB	1.001679	0.621611	-0.68834	3.93E-07	2.91E-06
DAW1	0.828463	0.258176	-1.68208	0.000152	0.000574
SIGLEC8	0.213844	0.796325	1.896801	3.20E-27	1.21E-25
RAPGEFL1	2.965738	1.964541	-0.5942	3.09E-05	0.000141
LY6H	2.686195	0.586717	-2.19483	0.004058	0.009914
INHBA	2.243886	5.204011	1.213625	1.17E-10	1.57E-09
TMEM178A	4.364554	2.339173	-0.89984	6.35E-08	5.57E-07
C7orf61	1.052439	0.642826	-0.71124	0.002238	0.005931
CYP1B1	3.501737	6.177184	0.818879	3.98E-08	3.60E-07
ARHGEF6	0.959532	2.033422	1.083507	2.82E-31	1.35E-29
ANKRD53	1.14201	0.651799	-0.80908	1.47E-06	9.52E-06
FAXC	0.384393	0.206589	-0.89582	3.09E-05	0.000141
RCSD1	0.582065	1.514584	1.379669	4.83E-37	3.34E-35
SLC7A3	1.35963	0.581726	-1.2248	0.00054	0.001732

Gene	conMean	treatMean	logFC	pValue	FDR
IL16	0.452248	1.06892	1.240967	1.35E-31	6.53E-30
ALX4	0.353704	0.137601	-1.36205	0.004689	0.011247
HIF3A	5.530299	3.315234	-0.73825	0.000233	0.000831
DNAH2	0.660038	0.407011	-0.69748	1.00E-07	8.46E-07
LAMA1	1.358738	0.619032	-1.13418	2.73E-06	1.64E-05
SIGLEC9	0.680734	2.019204	1.568623	2.61E-43	3.45E-41
SOX6	0.880907	0.476521	-0.88645	2.70E-08	2.54E-07
VAMP5	10.42143	22.06401	1.082141	2.55E-33	1.38E-31
MAP7D2	1.821274	1.066083	-0.77263	0.000364	0.00123
GAPT	0.454634	1.317193	1.534689	4.85E-26	1.69E-24
COL9A3	4.703674	0.979341	-2.2639	9.40E-11	1.28E-09
WDR17	0.205013	0.113574	-0.85208	0.000394	0.001318
TMEM198	2.938995	1.373762	-1.09719	5.89E-10	7.36E-09
BEX5	5.386479	3.192778	-0.75453	0.001473	0.004115
MT1M	3.512709	7.271085	1.049587	9.78E-10	1.18E-08
LGALS9	13.91321	21.6759	0.639637	4.76E-16	1.03E-14
FBXO2	15.06363	9.047366	-0.7355	3.04E-05	0.000139
ATP2B3	0.222813	0.117316	-0.92543	9.45E-09	9.71E-08
GPR17	0.732773	0.31122	-1.23543	7.17E-07	5.03E-06
MAN1A1	4.065609	8.529588	1.069005	3.84E-20	9.94E-19
C17orf50	0.296691	0.153539	-0.95035	2.87E-10	3.72E-09
FAM183A	9.314171	3.561137	-1.38709	6.11E-05	0.000257
PCSK1N	47.8831	22.38596	-1.09692	0.000396	0.001326
MZB1	1.539285	5.847403	1.925535	2.12E-19	5.33E-18
MRLN	0.151612	0.066652	-1.18567	0.000583	0.001852
UMODL1	0.300506	0.106352	-1.49855	0.001661	0.004572
HTR7	0.062442	0.169071	1.43705	5.47E-25	1.83E-23
PLPPR1	0.213373	0.079678	-1.42112	4.59E-07	3.34E-06
COLGALT2	0.46285	0.23312	-0.98947	0.008018	0.017791
PIEZO1	4.433271	6.722725	0.600675	8.86E-07	6.06E-06
COX6B2	0.284706	0.185253	-0.61998	0.026592	0.049176
ZNF732	0.893353	0.586058	-0.60819	8.69E-05	0.000351
CD3G	0.250587	0.974761	1.959738	3.16E-41	3.15E-39
GPX2	0.468502	0.188213	-1.31569	0.001487	0.00415
LGALS12	0.098461	0.240868	1.290615	5.49E-17	1.26E-15
FGFRL1	68.61344	44.02942	-0.64002	2.49E-06	1.52E-05
VSTM2B	1.432759	0.550247	-1.38064	2.89E-11	4.18E-10
RXFP4	0.287248	0.176197	-0.70511	9.91E-05	0.000393
C20orf96	19.59944	12.84068	-0.61009	7.99E-12	1.24E-10
CYP2C8	0.454134	0.254668	-0.8345	1.45E-05	7.28E-05
ENTPD2	10.05878	6.19906	-0.69833	0.000511	0.001649
MMP11	24.36499	46.61125	0.935869	1.50E-06	9.66E-06
LPAR6	2.57344	4.606319	0.839916	6.18E-21	1.67E-19
EFCAB12	0.830543	0.406578	-1.03052	1.18E-08	1.19E-07
ACSL5	7.946194	12.16986	0.614976	2.42E-11	3.53E-10
ELL2	1.204576	2.327146	0.950037	1.57E-22	4.63E-21

Gene	conMean	treatMean	logFC	pValue	FDR
C2orf50	1.302122	0.709914	-0.87515	0.004118	0.010034
ITIH2	1.578296	0.180852	-3.12548	3.66E-06	2.14E-05
AMHR2	0.656424	0.299427	-1.13242	1.37E-06	8.93E-06
CCDC27	0.282839	0.169199	-0.74126	2.90E-08	2.70E-07
GLI2	2.332248	1.538991	-0.59974	3.69E-05	0.000165
DNAAF1	0.74243	0.370671	-1.00212	0.000867	0.002612
APOE	116.1115	228.0668	0.973945	2.01E-23	6.16E-22
HBEGF	3.039455	5.271825	0.79449	1.50E-10	2.00E-09
SERPINB4	0.091205	0.264429	1.535694	0.017668	0.034881
POU2F3	3.008788	4.854865	0.690249	9.64E-06	5.04E-05
SPATA4	0.192141	0.093408	-1.04054	9.98E-05	0.000395
TLR7	0.490007	1.574848	1.684337	3.18E-37	2.23E-35
LRRC38	0.172236	0.09409	-0.87227	0.021522	0.041137
FPR1	1.182879	4.004763	1.759415	2.89E-34	1.69E-32
PCDHB6	1.766297	0.70348	-1.32815	4.19E-07	3.07E-06
PIGR	5.068102	10.57636	1.061326	9.66E-08	8.17E-07
CD300A	1.519012	4.05291	1.415825	9.60E-42	1.01E-39
ZFP42	1.315304	0.647017	-1.02352	0.012284	0.025684
BLOC1S5-TXNDC5	0.163024	0.102348	-0.6716	0.001664	0.004577
LEFTY1	1.781789	0.372784	-2.25691	5.53E-06	3.09E-05
GJB7	0.434338	0.214348	-1.01886	2.06E-05	9.85E-05
KIF1A	13.20721	7.082728	-0.89895	3.30E-08	3.04E-07
ZNF84	3.295015	2.168536	-0.60356	2.95E-11	4.26E-10
ZNF229	2.175954	1.360867	-0.67712	9.14E-05	0.000366
TCERG1L	0.220289	0.061721	-1.83557	2.45E-06	1.49E-05
ZNF334	2.211599	1.349107	-0.71309	1.15E-08	1.16E-07
FXYD1	2.87535	1.059731	-1.44004	3.70E-09	4.13E-08
COX6A2	0.736881	0.421769	-0.80498	0.000113	0.000441
ITGA11	2.180706	4.748424	1.122654	1.97E-11	2.89E-10
S1PR4	0.450366	1.447652	1.684544	1.15E-44	1.81E-42
CEACAM4	0.053695	0.209358	1.963115	1.21E-31	5.85E-30
AC018630.2	0.149932	0.100237	-0.58089	5.90E-05	0.000249
CSF3R	1.546139	4.042845	1.3867	2.13E-27	8.09E-26
PKIA	1.914755	1.042844	-0.87664	0.002217	0.005883
ZNF709	0.320432	0.193817	-0.72532	1.13E-07	9.44E-07
TMEM150B	0.425977	1.222563	1.521061	3.25E-37	2.27E-35
GNG3	0.744632	0.38495	-0.95186	4.25E-07	3.11E-06
EMILIN3	1.020856	0.260909	-1.96816	2.07E-07	1.64E-06
IL1RN	4.406616	7.513215	0.769759	6.22E-13	1.08E-11
HLA-DQB1	17.83326	48.01605	1.428946	1.58E-26	5.68E-25
GPR135	0.791917	0.474717	-0.73828	1.42E-08	1.40E-07
PLA2G4F	0.27809	0.145299	-0.93653	5.02E-05	0.000216
KCP	1.407652	0.667375	-1.07672	4.00E-08	3.62E-07
MEI1	0.313324	0.572679	0.870072	8.16E-22	2.30E-20

Gene	conMean	treatMean	logFC	pValue	FDR
ARHGAP15	0.200611	0.601756	1.58478	4.51E-50	2.36E-47
SPACA3	0.13979	0.062029	-1.17224	0.000738	0.002271
CD300E	0.233285	0.659917	1.500189	1.56E-22	4.60E-21
S100A2	17.0465	27.26087	0.677356	0.01053	0.022483
MPP1	2.114561	3.750685	0.826796	1.64E-30	7.42E-29
SPEF1	4.057649	2.104304	-0.9473	2.61E-07	2.01E-06
CPNE6	0.193584	0.053107	-1.86599	0.002254	0.005969
IRAK3	0.446642	0.79793	0.837144	2.78E-13	4.97E-12
PIK3CG	0.276878	0.814641	1.556916	1.94E-33	1.07E-31
EPSTI1	4.421985	8.292955	0.907192	3.66E-19	9.09E-18
GZMA	1.488547	6.584629	2.145198	1.98E-45	3.69E-43
SLC19A3	1.01881	1.852299	0.862431	5.53E-07	3.96E-06
GPR162	3.445648	2.278255	-0.59685	0.000551	0.001764
DAB2	4.393872	7.195457	0.711594	6.22E-19	1.53E-17
PLAU	13.19132	31.22352	1.243044	4.08E-20	1.05E-18
TRPM2	2.221079	3.600611	0.696981	1.55E-15	3.27E-14
GPR50	0.211473	0.116444	-0.86084	8.78E-05	0.000354
GZMM	0.298653	1.168268	1.967831	2.92E-44	4.40E-42
TMEM190	3.747497	1.454133	-1.36577	9.36E-08	7.96E-07
IL18BP	2.494965	4.665357	0.902968	9.86E-27	3.59E-25
SFTPB	0.493671	0.776434	0.653313	0.000249	0.000883
LRRC15	2.131778	4.626276	1.117794	1.86E-09	2.18E-08
THBS4	3.462392	1.795161	-0.94766	1.64E-05	8.08E-05
MAPK4	0.257126	0.095804	-1.42431	7.04E-08	6.14E-07
FYB2	0.959826	0.500858	-0.93837	0.012192	0.025519
KCNIP1	1.763025	0.724236	-1.28352	1.49E-05	7.42E-05
HS3ST2	0.327687	0.549124	0.744812	0.003507	0.008751
HRH2	0.399128	0.684557	0.778318	5.26E-12	8.34E-11
UROC1	0.152089	0.088283	-0.7847	0.012838	0.026704
IL21R	0.152858	0.812758	2.410631	1.35E-44	2.10E-42
MYOC	1.234654	0.202233	-2.61002	0.003682	0.009124
DIRC1	0.043542	0.165333	1.924903	2.79E-10	3.62E-09
COL16A1	3.288954	5.019485	0.60991	4.01E-07	2.96E-06
HLA-DPB1	42.93522	121.3246	1.498638	5.08E-39	4.05E-37
APOBEC3H	0.381366	0.954832	1.32407	8.80E-33	4.54E-31
TNFAIP8L2	1.586251	4.893287	1.625183	6.24E-51	4.21E-48
GJA8	0.54428	0.312412	-0.8009	0.014909	0.03023
SLCO6A1	0.135846	0.070908	-0.93795	2.25E-05	0.000106
TMC8	1.371722	3.061207	1.158113	5.31E-34	3.01E-32
NANOS3	1.650652	1.093967	-0.59347	5.70E-06	3.17E-05
SLAMF1	0.095368	0.42234	2.146825	6.28E-43	7.80E-41
WSCD2	0.566273	0.195142	-1.53697	0.023937	0.044983
PIK3R6	0.393646	0.708724	0.848327	6.43E-25	2.14E-23
CD300C	0.53143	1.66538	1.647898	3.55E-42	3.96E-40
PLPPR3	0.997695	0.481779	-1.05023	0.019414	0.03778
HLA-DMA	31.19355	67.76423	1.119276	7.82E-27	2.86E-25

Gene	conMean	treatMean	logFC	pValue	FDR
SMPDL3A	1.384374	2.431805	0.812794	1.18E-22	3.51E-21
CERS1	0.495387	0.293285	-0.75626	4.45E-11	6.30E-10
CD28	0.234303	0.432996	0.885978	1.32E-16	2.96E-15
SMIM17	0.319494	0.207688	-0.62137	0.001035	0.003045
EVI2A	1.642562	4.974829	1.598699	9.94E-45	1.60E-42
LILRA2	0.194734	0.527004	1.436307	2.02E-30	9.08E-29
TREML1	0.19513	0.410285	1.072193	4.27E-16	9.35E-15
COL6A2	74.65886	113.8706	0.60901	5.88E-06	3.26E-05
MLXIPL	6.698269	3.46654	-0.95029	4.38E-07	3.19E-06
PLXNC1	1.369012	2.293795	0.744601	2.18E-21	6.00E-20
PSMB9	12.98289	29.90935	1.203986	6.28E-24	1.98E-22
SERPINF1	21.72716	38.97876	0.843188	2.33E-15	4.88E-14
VNN1	1.18896	1.964314	0.724325	4.74E-17	1.09E-15
PLCB1	1.791609	1.162368	-0.62419	1.31E-07	1.08E-06
C11orf97	1.061483	0.31343	-1.75986	0.001251	0.003578
ZNF723	0.179692	0.114901	-0.64513	0.001139	0.003296
USH1G	0.316801	0.184054	-0.78345	0.008391	0.018501
CX3CR1	2.208005	4.781927	1.114848	8.42E-12	1.30E-10
DISP3	0.631887	0.116875	-2.43471	1.99E-13	3.65E-12
GBX1	0.170967	0.086005	-0.99122	3.69E-05	0.000165
ETV7	2.160683	4.474514	1.050244	5.08E-19	1.26E-17
CAMK2N2	2.7355	1.642014	-0.73634	0.009711	0.020965
CDHR2	0.175243	0.112546	-0.63884	0.003025	0.007709
CCL7	0.654502	1.773396	1.438046	2.09E-12	3.43E-11
CXCL17	42.96809	77.63137	0.853374	6.48E-07	4.58E-06
AOAH	1.108362	3.007294	1.440037	1.92E-39	1.60E-37
ACSM5	0.102517	0.158044	0.624466	7.52E-11	1.04E-09
MLKL	0.73335	1.477064	1.010158	4.62E-31	2.17E-29
MRVI1	1.275424	2.3114	0.857789	4.26E-12	6.82E-11
SLC6A11	1.032054	0.631363	-0.70898	0.002176	0.005785
TIGIT	0.128152	0.519911	2.02041	1.95E-39	1.61E-37
PFN3	0.243039	0.124027	-0.97054	0.008268	0.01827
MAFB	15.05119	22.59864	0.586359	4.95E-12	7.91E-11
IFI30	0.448586	0.918402	1.033743	7.72E-28	3.00E-26
HYDIN	0.322751	0.157423	-1.03577	9.61E-11	1.31E-09
IGSF1	0.815366	0.463002	-0.81643	0.000122	0.000472
BATF	1.558997	3.417429	1.132294	1.16E-29	4.99E-28
CD4	7.665064	19.79099	1.368474	1.28E-43	1.76E-41
APBB1IP	1.460185	4.860831	1.735051	6.88E-47	1.87E-44
RNF213	7.344764	11.61809	0.661585	7.44E-12	1.16E-10
HLA-DQA2	7.343987	23.50991	1.678634	1.04E-16	2.36E-15
ADRB1	0.172834	0.308848	0.83751	8.35E-09	8.74E-08
TSPAN8	2.74012	1.658193	-0.72463	3.92E-06	2.27E-05
SLC35F1	0.343551	0.142503	-1.26953	0.001113	0.003233
IFIT2	8.006765	12.09502	0.595122	1.84E-08	1.79E-07

Gene	conMean	treatMean	logFC	pValue	FDR
SFRP2	27.69741	59.30107	1.098307	5.13E-07	3.69E-06
COL6A6	0.281908	0.43955	0.6408	8.15E-08	6.99E-07
TEX14	0.171036	0.105861	-0.69213	0.00072	0.002223
CCN4	1.546727	3.271406	1.080693	4.51E-13	7.91E-12
LRRC18	0.206894	0.09803	-1.0776	0.001952	0.005258
FLNC	4.620406	2.793497	-0.72595	0.013792	0.028337
SLC46A2	0.142832	0.219046	0.616918	5.45E-10	6.85E-09
SOBP	1.840426	1.064662	-0.78964	2.96E-05	0.000136
CARD6	1.717942	2.902543	0.756636	9.51E-15	1.91E-13
CDHR3	1.056634	0.365971	-1.52968	3.54E-08	3.24E-07
PTPRT	0.950419	0.330823	-1.5225	7.27E-08	6.32E-07
UNC5A	5.537409	9.680852	0.805923	4.72E-05	0.000204
BNC1	0.583866	1.310156	1.166029	6.21E-06	3.42E-05
PIK3CD	2.926888	4.471412	0.611363	4.55E-12	7.27E-11
TMEM229B	0.626829	1.127126	0.846506	2.38E-18	5.77E-17
RAB7B	1.240774	2.234098	0.848452	5.88E-15	1.20E-13
CNGA4	0.406678	0.188469	-1.10956	0.000595	0.001885
CHGB	1.308117	0.146454	-3.15897	0.006897	0.015638
SOHLH2	0.159057	0.094875	-0.74543	0.000607	0.00192
CFAP300	3.103357	1.904067	-0.70475	2.00E-05	9.57E-05
SIGLEC10	0.806142	2.73027	1.759938	4.55E-41	4.39E-39
F13A1	4.640529	8.438679	0.862728	1.59E-11	2.37E-10
KCNJ12	1.741825	1.012892	-0.78212	8.09E-07	5.61E-06
LGR5	14.64064	5.194224	-1.495	1.08E-10	1.46E-09
CCL23	0.088301	0.276263	1.645546	2.32E-23	7.07E-22
SERPINE1	13.6753	32.45592	1.246909	2.94E-08	2.73E-07
TPPP3	18.18788	9.236045	-0.97763	9.56E-09	9.79E-08
RHBDL3	0.353782	0.150346	-1.23457	0.012953	0.026886
IKZF3	0.478026	1.033029	1.11172	2.49E-22	7.27E-21
CD244	0.069359	0.287223	2.050018	2.57E-43	3.44E-41
ASXL3	0.211421	0.072753	-1.53903	1.34E-06	8.76E-06
SH2D1A	0.156811	0.675442	2.106803	1.37E-41	1.39E-39
TUBB4A	8.846317	4.738672	-0.90059	3.48E-05	0.000157
PARVG	1.296086	2.030121	0.647404	2.35E-15	4.91E-14
IRF8	0.989213	2.567885	1.376227	3.88E-35	2.40E-33
C5AR1	3.646301	8.05188	1.142892	6.28E-24	1.98E-22
SAMD11	6.929574	3.349801	-1.04869	1.96E-05	9.43E-05
C2CD4B	3.279262	5.789843	0.820153	0.000199	0.000726
FOXI3	2.731226	1.706055	-0.67888	0.026752	0.049407
FNDC1	3.799424	8.000925	1.074386	4.74E-08	4.24E-07
NTF3	2.806419	1.728921	-0.69886	0.002471	0.006473
ABLIM3	0.392009	0.608232	0.633736	2.02E-09	2.35E-08
FSTL5	0.150177	0.087132	-0.78539	0.003054	0.007774
PAPPA2	0.43927	0.034059	-3.68902	0.002533	0.006614
SPN	0.406049	1.0117	1.317055	1.46E-41	1.48E-39
ZNF469	1.38461	2.473722	0.837203	4.80E-07	3.47E-06

Gene	conMean	treatMean	logFC	pValue	FDR
DRC7	0.703658	0.358761	-0.97185	5.15E-05	0.000221
IL10	0.773218	1.471695	0.928531	1.16E-15	2.48E-14
PTCH2	1.261127	0.735302	-0.77831	2.26E-06	1.39E-05
CDK5R2	0.464243	0.195885	-1.24487	0.018003	0.035433
ISM1	3.071503	4.778409	0.637586	2.29E-05	0.000108
CFAP46	0.489628	0.267553	-0.87186	1.72E-05	8.43E-05
DIO2	0.454124	0.888132	0.967688	6.52E-09	6.96E-08
OR2T8	0.260102	0.110809	-1.23101	0.004988	0.011856
CSTA	2.769034	4.888989	0.820153	7.23E-13	1.24E-11
ADAMTS12	1.483253	2.549831	0.781637	1.12E-05	5.73E-05
XCL2	0.598932	2.659799	2.150853	4.28E-40	3.72E-38
LRGUK	0.643409	0.421496	-0.61022	5.43E-06	3.04E-05
PTCRA	0.062307	0.175282	1.4922	2.25E-25	7.76E-24
MAATS1	1.686282	0.99515	-0.76086	3.93E-09	4.38E-08
OLFML2B	7.591406	11.70109	0.624204	3.28E-13	5.82E-12
TRIM22	5.828108	11.65409	0.999737	1.43E-23	4.41E-22
MYO1G	0.531531	1.247155	1.230415	2.24E-36	1.53E-34
ADAM8	4.462303	9.165149	1.03837	3.56E-22	1.03E-20
MMP7	74.71379	120.7807	0.692944	0.001228	0.003522
ZDHHC11B	0.771894	0.438011	-0.81744	1.00E-10	1.36E-09
ZNF208	0.894056	0.44549	-1.00497	9.15E-06	4.82E-05
RBM20	0.197575	0.127157	-0.63579	8.83E-05	0.000355
CLEC4E	0.386161	1.688235	2.12824	2.46E-39	2.01E-37
SALL1	1.851026	0.472356	-1.97038	0.000435	0.001435
CFAP206	0.220377	0.141458	-0.63959	0.002855	0.007344
ZCCHC18	0.304425	0.168327	-0.85482	5.55E-07	3.98E-06
ZKSCAN7	1.652597	1.094552	-0.59439	6.60E-11	9.19E-10
ARNT2	6.217314	3.700542	-0.74855	1.64E-08	1.61E-07
KIAA1549	2.33583	1.246078	-0.90654	6.14E-13	1.06E-11
ADAM11	0.630205	0.380813	-0.72674	3.24E-06	1.92E-05
HLA-DRB1	182.9028	458.2115	1.324937	1.21E-31	5.87E-30
CTHRC1	25.48923	46.9318	0.880678	1.33E-08	1.32E-07
MEX3B	1.45957	0.850006	-0.78	0.000597	0.001891
GAB3	0.295233	0.768029	1.37931	3.19E-39	2.59E-37
KCNAB2	2.15122	3.424982	0.670942	2.36E-20	6.22E-19
PTPRC	1.024871	3.971295	1.954167	6.84E-54	1.61E-50
SIGLEC7	0.585734	1.628619	1.475332	3.88E-35	2.40E-33
SULF1	9.234977	22.9895	1.315795	5.15E-15	1.05E-13
KLHL41	0.355639	0.134008	-1.40809	1.43E-08	1.42E-07
MEFV	0.079125	0.166086	1.069729	3.94E-24	1.26E-22
ARMC4	0.641451	0.296387	-1.11386	8.80E-09	9.17E-08
TNFRSF1B	4.791608	10.65009	1.152284	2.11E-30	9.45E-29
CD40LG	0.103284	0.438454	2.085813	8.91E-35	5.47E-33
PDGFRA	2.104513	3.147616	0.580773	1.69E-05	8.31E-05
ANTXR2	1.161513	1.839129	0.663017	4.82E-11	6.82E-10

Gene	conMean	treatMean	logFC	pValue	FDR
LYNX1	5.32431	8.929081	0.745917	0.006975	0.015784
EREG	0.088818	0.176471	0.990505	1.99E-06	1.25E-05
IGFL2	0.455664	1.910213	2.067692	6.32E-18	1.51E-16
ZNF738	8.080041	4.853339	-0.73538	5.42E-10	6.82E-09
ANO2	0.878363	0.474561	-0.88822	4.47E-08	4.02E-07
PRLH	0.459676	0.178826	-1.36207	0.013895	0.028522
MGAT5B	1.142317	0.481279	-1.24702	2.18E-05	0.000103
LRCOL1	0.344672	0.180949	-0.92964	0.000268	0.000944
ZNF781	0.272188	0.172162	-0.66084	0.009711	0.020965
ZBTB16	0.363209	0.642503	0.822904	2.61E-07	2.01E-06
NKX6-1	3.426209	1.107222	-1.62967	1.02E-05	5.31E-05
DNMT3A	7.273665	4.784516	-0.60431	5.51E-09	5.96E-08
UBXN10	4.772642	3.158235	-0.59567	0.00087	0.00262
IFI35	13.43451	23.23118	0.790118	6.51E-17	1.49E-15
SLIT1	0.372579	0.087753	-2.08603	0.000146	0.000554
GIMAP4	3.910678	11.02292	1.495016	1.79E-48	6.86E-46
CLEC5A	0.981894	2.642719	1.428384	1.02E-27	3.90E-26
CCL5	6.963152	32.07291	2.203543	6.85E-48	2.31E-45
ATP10A	1.23659	1.856396	0.586138	9.93E-09	1.01E-07
CLEC4M	0.265051	0.101055	-1.39113	0.000366	0.001237
GRAP2	0.074542	0.27682	1.892823	4.01E-41	3.95E-39
IQCN	4.336352	2.657537	-0.70639	2.16E-06	1.34E-05
CHI3L2	0.887055	2.018714	1.186341	7.85E-15	1.58E-13
PAPSS2	2.368631	3.98969	0.752223	2.50E-12	4.07E-11
STK33	1.29193	0.764268	-0.75738	9.40E-12	1.44E-10
CABCOCO1	1.522353	0.56914	-1.41945	5.70E-09	6.15E-08
ICAM3	0.129591	0.448721	1.791855	2.09E-39	1.73E-37
ZC3H12D	0.137202	0.316885	1.207651	5.20E-30	2.28E-28
MMP25	0.409823	0.646223	0.657032	1.93E-10	2.54E-09
DGKK	0.345153	0.166586	-1.05097	0.006019	0.013922
NGEF	5.252223	3.40201	-0.62654	0.001077	0.003145
CYP4F22	0.034966	0.248386	2.828547	1.12E-26	4.04E-25
CXCL14	12.34666	19.24505	0.640367	1.39E-06	9.05E-06
CHRND	0.279671	0.057985	-2.26997	5.03E-11	7.09E-10
CHRNG	0.346254	0.096638	-1.84116	1.03E-05	5.36E-05
IFT81	2.771738	1.719924	-0.68845	2.82E-14	5.52E-13
MS4A7	1.972777	5.150601	1.384513	1.17E-35	7.51E-34
CFAP221	0.426932	0.194454	-1.13458	2.76E-07	2.11E-06
GLIPR1	1.442132	3.046852	1.079116	6.36E-33	3.31E-31
FXYD4	3.166774	1.864185	-0.76447	4.13E-05	0.000182
GPNMB	14.95005	30.41151	1.024467	1.00E-21	2.82E-20
TOX2	2.905902	1.896344	-0.61577	0.002456	0.006437
SLCO1B3	0.128888	0.27652	1.10127	0.013584	0.027988
FBP2	0.143057	0.079089	-0.85503	0.001484	0.004146
ARHGAP31	1.1228	2.017225	0.845271	1.95E-18	4.74E-17
TNFAIP6	1.944008	3.746982	0.946695	3.50E-15	7.22E-14

Gene	conMean	treatMean	logFC	pValue	FDR
GNGT2	0.459305	0.985089	1.100802	7.65E-34	4.32E-32
RBPJL	2.326806	0.15599	-3.89882	4.60E-05	0.0002
TOX3	0.912494	0.201012	-2.18253	6.16E-06	3.40E-05
PTF1A	1.283284	0.106002	-3.59768	0.0002	0.00073
TNFRSF4	1.768859	3.303072	0.900989	4.53E-21	1.23E-19
KIF5C	2.546394	1.700709	-0.58232	3.10E-06	1.84E-05
HLA-DRB5	53.94572	137.4763	1.349602	1.14E-19	2.89E-18
CFAP52	0.717225	0.36184	-0.98707	8.24E-06	4.40E-05
ZSCAN23	0.272815	0.18047	-0.59617	5.34E-05	0.000228
LYN	5.877813	9.516139	0.695097	3.94E-22	1.14E-20
GFPT2	1.53821	2.627329	0.772344	2.45E-08	2.33E-07
SGK2	0.821678	0.462769	-0.82828	3.51E-05	0.000158
VGLL3	0.375551	0.83381	1.15071	3.28E-10	4.23E-09
WNK2	4.390131	2.265023	-0.95474	1.59E-12	2.66E-11
SLC5A5	0.45214	0.690029	0.609887	0.011319	0.023947
STK10	2.062999	3.351254	0.699958	2.59E-20	6.82E-19
FAM241A	0.323076	0.581197	0.847152	3.17E-20	8.26E-19
CALML5	5.797864	19.0672	1.7175	2.45E-06	1.50E-05
GLRA2	0.260623	0.150865	-0.78871	0.000216	0.000778
PLCH1	1.062302	0.547541	-0.95616	9.65E-07	6.54E-06
VAV1	1.825827	3.524929	0.949044	3.25E-25	1.11E-23
NOTUM	18.3608	2.270911	-3.01529	0.007274	0.016375
LTB	5.228298	13.26674	1.3434	5.24E-24	1.67E-22
USP44	0.53874	0.303637	-0.82724	7.86E-05	0.00032
RXRG	3.453229	1.009868	-1.77378	4.62E-07	3.35E-06
SBK2	1.963521	0.789617	-1.31422	0.001227	0.003522
BHLHE22	0.102968	0.299046	1.538177	2.77E-20	7.28E-19
FGFR3	6.160079	2.814401	-1.13012	1.20E-05	6.11E-05
IL12RB1	0.318999	1.055399	1.726167	8.23E-46	1.74E-43
RCOR2	11.82706	4.294972	-1.46137	7.20E-16	1.56E-14
C11orf96	6.189591	9.479366	0.614946	0.000316	0.001088
PIM2	10.30983	16.27496	0.658633	3.01E-11	4.33E-10
OLFM2	16.08627	8.9164	-0.8513	0.000624	0.001962
TTC29	0.304718	0.115586	-1.39851	0.001293	0.003683
LRRTM4	0.255777	0.141947	-0.84954	0.000272	0.000956
KREMEN2	5.854187	3.656978	-0.67882	8.26E-05	0.000335
RIN3	3.056166	4.598266	0.589367	1.45E-15	3.06E-14
GJC2	3.772878	1.859396	-1.02083	8.88E-06	4.69E-05
AC013470.2	0.214528	0.12981	-0.72476	3.68E-05	0.000164
RASL11A	6.088576	9.431237	0.631342	7.49E-06	4.05E-05
RNASE2	0.974485	3.080499	1.660453	2.01E-32	1.02E-30
FCGR3A	7.738628	25.983	1.747418	3.49E-41	3.46E-39
LY96	4.300129	11.66202	1.439365	1.09E-41	1.13E-39
CXCL12	7.909977	13.86351	0.809547	2.25E-09	2.60E-08
SAA1	18.20831	29.73771	0.707696	2.87E-11	4.16E-10

Gene	conMean	treatMean	logFC	pValue	FDR
FGF8	0.239804	0.030582	-2.97109	4.58E-07	3.33E-06
ALOX5AP	7.121158	25.42028	1.835796	2.13E-40	1.91E-38
VWA5B1	0.145181	0.064056	-1.18045	7.87E-06	4.23E-05
PCSK2	1.125122	0.580248	-0.95534	0.000247	0.000875
FANK1	1.731263	1.149766	-0.59048	2.05E-06	1.28E-05
NPIPB6	0.213075	0.135164	-0.65665	3.71E-06	2.16E-05
SCG5	11.06614	5.614406	-0.97895	1.75E-05	8.55E-05
TAS2R31	0.210458	0.138804	-0.60048	7.60E-05	0.000311
ROR1	3.634994	2.318087	-0.64902	5.69E-05	0.000241
FGF17	5.024938	0.211707	-4.56896	8.45E-17	1.92E-15
LRRC73	2.523321	1.462025	-0.78736	6.09E-06	3.36E-05
PRSS3	0.741761	0.261635	-1.5034	0.000985	0.002916
ICA1L	0.963259	0.60001	-0.68294	3.83E-12	6.15E-11
P4HA3	0.412357	0.684338	0.730814	4.79E-08	4.28E-07
SRSF12	1.282398	0.780005	-0.71729	1.17E-06	7.74E-06
C10orf82	1.079425	0.392287	-1.46028	1.26E-08	1.26E-07
PRSS56	0.202104	0.043173	-2.2269	6.17E-10	7.68E-09
DPEP2	0.285953	0.620251	1.117073	6.48E-28	2.54E-26
EFS	7.388144	4.779816	-0.62826	0.002502	0.006542
MTMR7	0.52302	0.348575	-0.5854	2.14E-07	1.69E-06
DNAI2	0.466144	0.171137	-1.44562	3.46E-07	2.59E-06
SLC22A23	3.382242	2.196191	-0.62298	0.000399	0.001334
LRRC52	0.29357	0.108453	-1.43664	1.11E-05	5.72E-05
TTC21A	1.271322	0.822661	-0.62796	1.98E-06	1.24E-05
HAPLN2	0.259223	0.147799	-0.81056	8.28E-06	4.42E-05
SORCS1	0.686576	0.191878	-1.83923	0.001014	0.002993
CHRNA6	0.107448	0.239903	1.158818	7.85E-11	1.08E-09
DUSP15	2.003493	0.978523	-1.03384	5.95E-12	9.40E-11
STUM	0.54064	0.257788	-1.06848	6.17E-06	3.40E-05
CD69	0.847784	2.545512	1.586187	5.18E-32	2.56E-30
HLA-DRA	379.567	972.445	1.357262	2.44E-34	1.43E-32
MMP2	39.04674	72.63186	0.8954	7.74E-08	6.67E-07
PCLO	0.372051	0.216005	-0.78443	4.73E-07	3.43E-06
CCL8	2.103253	6.32162	1.587672	3.44E-24	1.11E-22
LAX1	0.150451	0.532477	1.823424	3.88E-23	1.17E-21
HMGCLL1	0.25193	0.118253	-1.09114	0.002708	0.007017
LGI4	0.582225	0.364377	-0.67614	0.023882	0.044897
FCGR2B	0.691333	2.50346	1.85647	4.57E-46	1.06E-43
MATN1	0.257154	0.102563	-1.32612	0.007005	0.015843
TMC5	1.426761	2.210259	0.631472	0.006284	0.01442
SELP	0.274279	0.635862	1.213073	1.37E-14	2.74E-13
GPR150	0.075324	0.135369	0.845705	6.23E-12	9.79E-11
LRRC25	1.988031	5.379183	1.436047	8.02E-43	9.71E-41
C2	8.722981	13.87485	0.669579	1.04E-12	1.77E-11
SLC26A7	1.39652	0.517817	-1.43132	0.000131	0.000502
CRISPLD2	3.025676	6.391022	1.078789	2.61E-07	2.01E-06

Gene	conMean	treatMean	logFC	pValue	FDR
MMP12	1.233586	4.50656	1.869168	2.84E-09	3.22E-08
CORIN	0.127525	0.228667	0.842467	2.32E-08	2.21E-07
FFAR1	0.235955	0.121334	-0.95953	0.001128	0.003267
FER1L5	0.388501	0.253074	-0.61836	7.01E-06	3.82E-05
SBK1	14.33306	7.487428	-0.9368	1.19E-10	1.60E-09
SPRY2	13.83312	8.639748	-0.67907	0.000618	0.001946
GGT5	1.882601	3.304828	0.811848	2.06E-16	4.59E-15
PGAP4	4.718304	2.981447	-0.66226	8.21E-06	4.39E-05
CD52	8.352482	26.84674	1.68447	6.41E-38	4.73E-36
ACKR4	0.353052	0.624958	0.823879	2.18E-10	2.85E-09
CD5	0.380962	1.401205	1.87895	3.79E-42	4.19E-40
HLA-DQB2	2.800215	8.670692	1.630609	3.78E-26	1.33E-24
GATA4	2.190734	1.445408	-0.59994	0.00102	0.003007
KCNK13	0.685986	1.032947	0.590515	5.77E-13	1.00E-11
CCL2	15.07327	33.4705	1.150898	3.96E-29	1.64E-27
GIMAP1	0.380129	0.945854	1.31513	6.34E-39	4.96E-37
COL1A1	349.27	655.4572	0.908159	2.06E-07	1.63E-06
POPDC3	1.007263	0.4386	-1.19946	0.000741	0.00228
KCNK3	1.377395	0.787649	-0.80632	0.010306	0.022064
CD226	0.063538	0.155749	1.293531	1.35E-33	7.54E-32
DNAJC5B	0.090435	0.238881	1.401338	5.68E-23	1.71E-21
SCGB1D4	3.023566	0.443836	-2.76815	2.80E-05	0.000129
PTGER2	2.713438	4.34896	0.680548	6.35E-11	8.88E-10
CARD17	0.176461	0.421988	1.257854	5.02E-14	9.66E-13
LTBP4	34.05228	15.80585	-1.10729	4.24E-08	3.82E-07
PIANP	3.311108	1.52255	-1.12082	7.96E-09	8.37E-08
GPSM3	9.112581	18.74832	1.04083	7.21E-31	3.35E-29
AP006333.1	0.534457	0.151065	-1.8229	7.82E-08	6.72E-07
ZMYND15	1.931628	2.915922	0.594135	5.69E-10	7.12E-09
KIRREL2	1.899265	0.576848	-1.71918	9.00E-05	0.000361
CPA1	2.674531	0.209301	-3.67564	1.28E-06	8.44E-06
SP5	6.674868	2.092069	-1.67381	2.76E-08	2.59E-07
THBS2	10.71588	27.526	1.361044	6.52E-14	1.24E-12
BLNK	1.433724	2.150089	0.584629	6.11E-14	1.17E-12
NLRC4	0.349739	0.650906	0.896169	1.13E-28	4.55E-27
GPRIN3	0.309159	0.690481	1.159251	5.56E-36	3.63E-34
PSD2	0.50536	0.310416	-0.70311	0.000303	0.00105
STAC3	1.157459	1.747988	0.594734	6.83E-16	1.48E-14
EMX1	0.273093	0.075075	-1.86299	0.003226	0.008146
TMEM273	0.40699	0.96838	1.250579	4.47E-34	2.57E-32
LILRA1	0.147276	0.389464	1.402966	3.92E-29	1.63E-27
LMNTD1	0.143201	0.05719	-1.32421	0.0073	0.016428
SLC31A2	0.219545	0.441546	1.008051	3.60E-23	1.09E-21
ZNF648	0.152144	0.088635	-0.77949	2.65E-08	2.50E-07
KIR2DL4	0.091063	0.413271	2.182153	9.30E-28	3.58E-26

Gene	conMean	treatMean	logFC	pValue	FDR
TMEM106A	1.598731	2.548906	0.672951	5.04E-19	1.25E-17
ACP3	2.854034	5.973588	1.065595	1.47E-05	7.36E-05
SNCB	0.769788	0.276827	-1.47548	1.43E-05	7.17E-05
RAB38	6.384533	3.743931	-0.77003	1.17E-06	7.74E-06
CTXN1	165.437	110.2715	-0.58522	0.000277	0.000969
CBY2	0.403422	0.249985	-0.69045	2.71E-05	0.000125
CCR7	0.37621	1.198443	1.67155	7.78E-33	4.02E-31
PCP4	24.17012	11.64313	-1.05375	7.85E-09	8.26E-08
CD163	3.057507	9.26502	1.599438	2.07E-35	1.30E-33
RASAL3	0.617284	1.943567	1.6547	2.37E-46	6.00E-44
ACKR1	1.363489	2.590563	0.925962	1.80E-09	2.11E-08
COL5A2	13.09151	24.48835	0.903464	2.60E-09	2.97E-08
NRTN	8.03519	4.947394	-0.69966	6.04E-05	0.000254
PSTPIP2	1.972991	3.693371	0.904554	2.59E-16	5.73E-15
NAP1L2	0.694212	0.450887	-0.62261	0.00139	0.00391
NPHP1	1.197079	0.788905	-0.60159	3.85E-11	5.49E-10
CATSPER1	0.184146	0.327699	0.831522	7.16E-30	3.12E-28
STARD8	0.725796	1.130135	0.638858	7.80E-20	1.99E-18
THBD	1.912667	3.770982	0.979355	1.58E-12	2.64E-11
ODF3B	7.101246	10.91282	0.61988	6.62E-15	1.35E-13
FCRLA	0.052939	0.182207	1.78318	4.50E-24	1.44E-22
HNF1A	0.231743	0.015302	-3.92073	7.00E-05	0.000289
HOXD1	5.426097	8.187004	0.593421	0.001571	0.004354
NR2F6	117.2769	77.77677	-0.59251	1.46E-09	1.73E-08
COL4A6	0.525557	0.247159	-1.08841	4.44E-06	2.54E-05
KRT6A	6.847011	11.97181	0.806096	7.32E-05	0.000301
SLC25A18	0.344876	0.185185	-0.89712	0.001025	0.003018
ZNF703	33.6623	20.29998	-0.72966	1.10E-05	5.65E-05
PPEF1	0.127166	0.248684	0.967603	6.95E-07	4.88E-06
CLLU1-AS1	0.048928	0.167572	1.776043	4.16E-05	0.000183
GFRA3	3.154565	0.683646	-2.20612	0.001431	0.004013
HNF1B	1.087631	0.451918	-1.26706	6.23E-07	4.41E-06
PCDHGA1	0.333259	0.204236	-0.70641	4.81E-05	0.000208
CBLN4	0.131765	0.221588	0.749908	9.27E-09	9.56E-08
LINGO3	0.152045	0.233783	0.620671	8.37E-13	1.43E-11
MICAL1	2.070833	3.142216	0.601571	1.84E-13	3.39E-12
LRP4	3.073211	0.93653	-1.71435	3.60E-07	2.68E-06
LTA	0.191733	0.706836	1.882279	6.32E-44	9.13E-42
PCDHB2	4.09569	2.660036	-0.62266	0.000542	0.001737
CD1E	0.107876	0.876452	3.022305	4.06E-30	1.79E-28
LRFN1	5.027346	3.241029	-0.63334	9.64E-06	5.04E-05
RNF224	1.156173	0.580571	-0.99381	8.01E-06	4.30E-05
LKAAEAR1	2.30046	0.825104	-1.47927	1.02E-11	1.56E-10
SCGB1D1	26.7109	5.211664	-2.35761	0.014622	0.029802
TMEM72	0.502192	0.070035	-2.8421	4.55E-10	5.78E-09
VWC2	0.190728	0.066169	-1.52729	9.52E-07	6.46E-06

Gene	conMean	treatMean	logFC	pValue	FDR
LHFPL2	3.130922	5.949562	0.926196	8.90E-33	4.57E-31
CCL4	2.623926	7.895822	1.589363	4.17E-41	4.07E-39
KCNIP3	2.497694	1.48808	-0.74714	9.09E-09	9.41E-08
TNF	3.51355	6.558114	0.900351	3.55E-10	4.56E-09
ANKRD33	0.267589	0.071805	-1.89786	5.13E-05	0.00022
SLC26A9	0.915453	1.480075	0.693112	7.53E-05	0.000308
KRT31	0.210234	0.034727	-2.59789	0.007135	0.016101
CAVIN4	0.565149	0.333641	-0.76033	6.52E-09	6.96E-08
SPHK1	1.515187	3.0245	0.9972	5.17E-17	1.19E-15
CYP8B1	0.109427	0.177061	0.694284	0.00012	0.000464
DOCK8	0.69758	1.658114	1.249112	8.40E-31	3.89E-29
CFAP99	0.177755	0.118769	-0.58173	0.004106	0.010018
CADM4	13.50766	8.919421	-0.59876	1.49E-05	7.43E-05
CD6	0.742294	1.276148	0.781734	1.24E-20	3.32E-19
PODXL	43.53409	29.06244	-0.58299	2.22E-05	0.000105
CXCL10	28.47221	98.65058	1.792773	1.22E-28	4.89E-27
MTTP	0.164397	0.066699	-1.30145	3.40E-07	2.55E-06
CD1B	0.03309	0.253548	2.937785	2.93E-24	9.44E-23
NLRC5	1.376828	2.565141	0.89769	7.10E-17	1.62E-15
EFHB	0.619253	0.298347	-1.05354	1.13E-06	7.54E-06
OTUD7A	0.156943	0.098647	-0.66989	0.003571	0.008883
ECEL1	6.184414	1.711738	-1.85317	8.79E-05	0.000354
KCNIP4	1.02835	0.568651	-0.85471	3.93E-07	2.91E-06
ECT2L	0.12336	0.077824	-0.66459	0.000198	0.000725
ADAM33	2.479394	1.482305	-0.74215	0.011169	0.023654
CHSY3	0.410891	0.769818	0.905763	8.27E-08	7.09E-07
SLC38A3	1.920178	1.077617	-0.8334	1.20E-05	6.11E-05
ADAMTS4	0.799435	1.28621	0.686075	3.48E-08	3.19E-07
TRPV2	1.375962	3.080719	1.162827	1.27E-38	9.75E-37
CCDC114	2.628995	1.617862	-0.70042	5.53E-05	0.000235
FCGR3B	0.111653	0.454821	2.026278	3.00E-15	6.21E-14
DDX25	0.439351	0.121577	-1.8535	1.74E-11	2.57E-10
S100A9	98.33578	173.9878	0.823198	2.10E-16	4.66E-15
LACC1	0.891328	1.421368	0.673252	2.76E-17	6.39E-16
P2RY14	0.164634	0.299131	0.861513	1.29E-20	3.43E-19
BTBD17	1.008918	0.059433	-4.0854	4.54E-08	4.08E-07
TRAT1	0.090361	0.465619	2.365371	8.66E-42	9.23E-40
INMT	0.534062	0.850168	0.670741	2.65E-06	1.60E-05
POU2AF1	0.527898	0.888698	0.751435	0.000226	0.000811
TTC9	3.997421	6.162218	0.62438	7.69E-06	4.14E-05
CD79A	2.020095	6.621579	1.712752	1.78E-15	3.74E-14
MACROH2A2	30.33885	19.92649	-0.60648	8.95E-09	9.28E-08
ECM2	1.044341	1.792612	0.779471	1.34E-06	8.75E-06
ALPK2	0.198374	0.311253	0.649864	1.22E-08	1.23E-07
CDH2	20.62138	11.1847	-0.88261	3.37E-07	2.53E-06

Gene	conMean	treatMean	logFC	pValue	FDR
SLC11A1	1.192407	2.331601	0.967444	1.30E-20	3.45E-19
IL10RA	1.203264	3.962055	1.719296	2.29E-43	3.12E-41
WNT2B	0.849125	0.445556	-0.93037	3.78E-06	2.20E-05
SLC15A3	6.959623	12.20552	0.810453	1.20E-17	2.84E-16
EFNA3	6.792371	4.246122	-0.67777	1.50E-06	9.63E-06
APLNR	1.835241	2.967301	0.693182	1.18E-06	7.80E-06
P2RY6	2.021077	3.771577	0.900044	2.83E-22	8.25E-21
PTPN5	0.227629	0.111929	-1.02409	8.24E-06	4.40E-05
KLRD1	0.078295	0.168413	1.105012	6.52E-21	1.75E-19
DERL3	4.429797	7.354144	0.731317	4.23E-09	4.70E-08
SLC35G6	0.142091	0.088043	-0.69054	0.003782	0.009327
CCL19	0.460156	2.838962	2.625168	1.42E-26	5.09E-25
TIGD3	0.876645	0.242334	-1.855	0.000402	0.001341
CCDC40	1.899747	0.977405	-0.95878	8.30E-10	1.02E-08
ASTN1	1.601887	0.50647	-1.66122	8.28E-07	5.72E-06
C10orf99	2.902599	10.12031	1.801836	0.025184	0.04701
NSG1	18.15058	11.0012	-0.72236	6.87E-08	5.99E-07
SCG3	1.024408	0.072187	-3.82691	0.008158	0.01807
IL27	0.072506	0.190509	1.393697	1.98E-23	6.06E-22
PPM1H	4.924819	3.203208	-0.62055	1.36E-06	8.88E-06
PKIB	0.998017	1.947519	0.964501	6.05E-22	1.72E-20
VCAN	4.726162	9.456048	1.000568	2.02E-10	2.66E-09
APOBR	0.83848	2.213523	1.400497	5.13E-34	2.93E-32
TFF1	2.05634	0.168682	-3.6077	3.18E-05	0.000145
FOLR2	5.446931	10.98388	1.011872	6.97E-20	1.78E-18
APOL6	4.806399	10.14201	1.077316	7.60E-24	2.38E-22
CACNG8	0.128522	0.080606	-0.67306	0.00112	0.003249
FGF19	2.019781	0.353643	-2.51383	1.02E-15	2.18E-14
ZBBX	0.804709	0.310353	-1.37456	8.64E-07	5.93E-06
HPSE	2.223227	4.257248	0.937266	2.11E-19	5.29E-18
SSC5D	2.00459	3.091967	0.625218	1.97E-06	1.23E-05
SQOR	4.412259	7.973501	0.853696	1.42E-22	4.20E-21

Table S3. The results of genomic instability-related differentially expressed genes (DEGs).

Gene	conMean	treatMean	logFC	pValue	FDR
ABCA6	0.073568	0.153095	1.057285	3.78E-07	0.004791
BAG2	1.871568	2.968229	0.665355	0.000143	0.025939
C16orf54	0.398591	0.634058	0.669706	0.00046	0.035186
HTRA4	0.102753	0.175708	0.774001	0.00018	0.025939
DRP2	0.114444	0.179498	0.649329	0.001276	0.047277
THPO	0.098318	0.228955	1.219533	3.10E-05	0.021617
LRRN4CL	0.979332	1.767611	0.851931	0.000627	0.03715
ISLR2	0.409441	1.161227	1.50392	0.00068	0.038474
GPR1	0.421521	0.820762	0.961359	0.001442	0.049442

Gene	conMean	treatMean	logFC	pValue	FDR
GABRR2	0.10977	0.192329	0.809092	0.000935	0.042313
FBXL22	0.718556	1.134047	0.658308	0.00068	0.038474
CNTN1	0.993437	1.889137	0.927226	0.000702	0.039194
FAM155A	0.155882	0.289977	0.895487	0.000297	0.03038
ALDH3B1	10.80585	7.051223	-0.61587	0.000242	0.027887
BASP1	10.3636	20.29371	0.969508	5.92E-06	0.020383
ITGA8	0.194544	0.342038	0.81406	0.000106	0.025939
B3GALT1	0.305479	0.777849	1.348415	0.000302	0.030653
NR5A2	0.187769	0.368382	0.972246	2.79E-05	0.021617
HEPH	1.014766	1.803658	0.829778	0.000168	0.025939
HIC1	1.151464	1.895696	0.719259	0.000156	0.025939
IGSF23	0.732768	1.79737	1.294458	0.000669	0.038474
HOXC8	0.642026	0.987821	0.621618	0.000598	0.036407
ABCA9	0.140587	0.286594	1.027543	6.14E-05	0.024699
ADAMDEC1	1.14333	2.915914	1.350706	0.00141	0.049201
DLX6	0.632645	1.216751	0.943566	2.06E-05	0.021617
PTPRO	0.3804	0.598842	0.654657	0.000346	0.032985
HOPX	1.855224	3.392268	0.870657	6.31E-05	0.024699
TMEM200A	0.683953	1.062299	0.63522	4.59E-05	0.021948
LHFPL6	5.262816	7.909948	0.587833	0.000226	0.027887
TSHR	0.299511	0.520257	0.796615	0.000691	0.038752
AOC3	1.05138	2.566121	1.287305	0.000215	0.027887
COPZ2	3.856309	5.765573	0.580243	0.000824	0.040625
RGS4	1.605362	2.850719	0.828427	0.000396	0.033898
LMOD1	1.959397	3.454712	0.818156	0.001464	0.049442
MS4A1	0.096875	0.276769	1.514482	0.001058	0.045062
THSD1	0.427892	0.701923	0.714067	2.29E-05	0.021617
PABPC5	0.151924	0.247648	0.704936	0.000761	0.039194
COLCA2	3.295163	1.492438	-1.14268	4.50E-05	0.021948
PTGDR	0.083365	0.127715	0.615405	0.00068	0.038474
DNMT3B	2.302009	3.500641	0.604726	0.000714	0.039194
PLAAT3	16.30328	10.33384	-0.65779	0.000452	0.035186
APOBEC3A	0.453104	0.794246	0.809744	0.000569	0.035702
KRTAP5-1	0.284923	0.485633	0.769294	0.000317	0.031381
MEIS3	1.015547	1.590484	0.647208	0.00018	0.025939
CCR4	0.148563	0.256241	0.786427	0.00092	0.042313
NEXN	1.291451	2.273901	0.816177	3.51E-05	0.021617
PLN	0.40556	1.117003	1.461646	0.000516	0.035186
CHRD	0.402683	1.067982	1.40717	4.68E-05	0.021948
GRAMD2A	5.353905	2.851307	-0.90897	0.000864	0.04179
ZNF804A	0.082811	0.128859	0.637891	0.00034	0.032929
PRDM8	0.180753	0.292854	0.696161	0.000508	0.035186
EBF1	0.736895	1.108774	0.589434	0.00037	0.033898
CCDC150	0.254527	0.381241	0.582888	0.000638	0.03758
ABCA10	0.068147	0.131455	0.947851	5.34E-05	0.024146

Gene	conMean	treatMean	logFC	pValue	FDR
SRPX2	3.045338	4.914691	0.690498	0.000278	0.029828
NUGGC	0.181753	0.323501	0.83179	0.000146	0.025939
RSPO3	4.00966	8.140341	1.021609	0.000598	0.036407
MCTP1	0.185913	0.318117	0.774932	0.000174	0.025939
PIWIL1	0.347224	0.180708	-0.9422	0.001101	0.045366
LILRA5	0.415453	0.637904	0.618654	0.000508	0.035186
BVES	0.500843	0.91978	0.876931	0.00106	0.045062
SOCS2	0.424843	0.647862	0.608757	0.000499	0.035186
AP1S2	4.979498	7.577374	0.605698	0.000165	0.025939
TCEAL7	0.962932	1.768394	0.876934	0.000177	0.025939
RASGRF2	0.39825	0.723309	0.860938	1.61E-05	0.021617
TLR4	0.962682	1.566563	0.702471	0.000607	0.036825
PTGIR	0.609934	0.933324	0.613725	0.000749	0.039194
IRF4	0.241893	0.563712	1.22059	0.000467	0.035186
MN1	1.063668	2.009895	0.918073	0.001442	0.049442
P2RY8	0.351589	0.59853	0.767533	0.001336	0.048155
GASK1B	2.460857	4.033687	0.712938	9.64E-05	0.025939
TM6SF1	0.452484	0.678328	0.584115	0.000154	0.025939
DDX43	0.198547	0.359772	0.857606	0.000227	0.027887
GRIP2	0.087939	0.136852	0.638039	0.000297	0.03038
FMN1	0.626164	0.39468	-0.66586	0.000579	0.035933
C5AR2	0.116009	0.191419	0.722495	0.00043	0.035186
PLA2G4C	0.319072	0.533613	0.741909	6.44E-06	0.020383
KRT18	339.6823	220.3003	-0.62471	0.000222	0.027887
DLX5	1.709954	3.407078	0.994578	0.001257	0.047277
KMO	0.176186	0.26654	0.597253	0.000154	0.025939
PECAM1	5.009312	7.510537	0.584304	8.48E-05	0.025939
DCN	16.67818	32.87781	0.979153	0.000162	0.025939
SMTN	3.724159	5.603823	0.589497	3.45E-05	0.021617
LILRB1	0.900975	1.403718	0.639694	0.000851	0.041291
CLEC7A	1.469858	2.206266	0.585931	0.001464	0.049442
DACT1	1.079059	1.784007	0.725348	0.000238	0.027887
PRDM1	1.492242	2.29517	0.621119	2.79E-05	0.021617
TPSAB1	1.197822	2.805579	1.227885	0.001509	0.049806
ZEB2	0.642159	1.064216	0.72879	8.64E-05	0.025939
CNRIP1	1.232433	2.00358	0.701071	9.30E-05	0.025939
ADCY8	1.977819	0.446162	-2.14827	0.000557	0.035632
CALHM5	0.264562	0.395891	0.581498	0.000136	0.025939
COMP	8.370188	19.54503	1.22347	0.000215	0.027887
ADAMTS2	2.699479	5.042421	0.901436	0.000761	0.039194
CXorf21	0.583227	0.896805	0.620737	0.000452	0.035186
GAS2L2	1.113716	0.336468	-1.72684	0.001336	0.048155
C2orf66	0.165719	0.400985	1.274805	0.001366	0.048334
PRDM6	0.143541	0.284753	0.988246	0.001027	0.044882
PDCD1LG2	0.767828	1.234667	0.685268	0.001464	0.049442
ISLR	37.82174	66.78339	0.820273	0.001464	0.049442

Gene	conMean	treatMean	logFC	pValue	FDR
LIN7B	0.921647	1.381543	0.583994	3.58E-05	0.021617
SLAMF7	1.003057	1.943562	0.9543	0.00151	0.049806
CELF4	0.856801	1.468579	0.77739	0.000283	0.029828
TBX4	0.214657	0.093738	-1.19533	0.001442	0.049442
HMGCS1	5.844693	9.453881	0.693779	0.000389	0.033898
KLF2	7.938779	14.19716	0.838613	0.001182	0.04649
CCL11	2.325847	4.560231	0.971351	0.000177	0.025939
UCN2	0.128902	0.194894	0.596419	0.001396	0.048854
GATA2	1.414965	3.528868	1.318439	0.000551	0.035433
FCAR	0.118583	0.231763	0.966749	0.000162	0.025939
SMOC2	5.065162	10.1645	1.004859	5.54E-05	0.024207
FAT4	0.240694	0.387195	0.685857	0.000491	0.035186
INHBA	2.505063	4.399989	0.812653	0.001027	0.044882
RUNX1T1	0.284083	0.506174	0.833321	0.001276	0.047277
FILIP1L	4.255818	6.884143	0.693841	0.000445	0.035186
S1PR1	2.339318	3.614838	0.627843	7.88E-05	0.025613
LPAR4	0.152594	0.277695	0.863804	0.000164	0.025939
SGCD	0.741476	1.252697	0.756565	0.000516	0.035186
RAMP3	1.725748	2.599243	0.59087	0.000377	0.033898
AC005020.2	0.144039	0.080511	-0.8392	0.000565	0.035702
ACSM5	0.088057	0.167412	0.926893	0.001487	0.049442
MRVI1	1.174131	2.39214	1.026708	0.000113	0.025939
SYNPO2	1.007912	1.802913	0.83896	0.000508	0.035186
CCN4	1.584092	3.327142	1.070627	0.000824	0.040625
IGFBP5	56.18184	90.81863	0.692885	0.001077	0.045313
ADAMTS5	0.86766	1.301873	0.585387	0.00106	0.045062
FNDC1	3.837628	8.265516	1.10689	0.000935	0.042313
IL10	0.808762	1.371465	0.761931	0.001163	0.046062
CDK5R2	0.172358	0.499301	1.534503	0.000949	0.042678
TKTL1	2.495043	4.119322	0.723342	0.001392	0.048854
PDGFRA	1.919995	3.370462	0.811844	0.000824	0.040625
ANTXR2	1.101384	1.747057	0.665609	0.000151	0.025939
CDH18	0.459361	0.930383	1.018198	0.00127	0.047277
GLIPR1	1.709011	2.555567	0.580482	3.01E-05	0.021617
C11orf96	5.730507	10.80138	0.914481	0.000508	0.035186
SEMA3C	5.413156	8.723142	0.688378	0.000669	0.038474
LAX1	0.175836	0.380951	1.115376	0.000177	0.025939
LEP	0.04492	0.214927	2.258411	0.000187	0.026267
B3GALNT1	7.639634	11.88289	0.637311	0.000136	0.025939
OR51E1	0.145885	0.218265	0.581248	0.001464	0.049442
CHSY3	0.377194	0.7149	0.922435	0.001219	0.046785
ADAMTS4	0.811358	1.470083	0.857488	0.001378	0.048489
P2RY14	0.163678	0.247332	0.595588	0.000438	0.035186
ECM2	1.030611	1.641632	0.671631	0.000569	0.035702
CCL19	1.200432	2.343964	0.965397	0.000533	0.035186

Gene	conMean	treatMean	logFC	pValue	FDR
VCAN	5.329961	9.191833	0.786227	0.000483	0.035186
KRT86	3.650036	1.475129	-1.30707	0.000935	0.042313

Table S4. Enriched gene sets.

Gene sets	Description	ES	NES	pvalue	FDR
KEGG	KEGG_NOD_LIKE_RECEPTOR_SIGNALING_PATHWAY	0.766	2.482	0.000	0.000
KEGG	KEGG_TOLL_LIKE_RECEPTOR_SIGNALING_PATHWAY	0.758	2.456	0.000	0.000
KEGG	KEGG_CYTOSOLIC_DNA_SENSING_PATHWAY	0.761	2.451	0.000	0.000
KEGG	KEGG_CHEMOKINE_SIGNALING_PATHWAY	0.668	2.396	0.000	0.000
KEGG	KEGG_NATURAL_KILLER_CELL_MEDIATED_CYTOTOXICITY	0.719	2.393	0.000	0.000
KEGG	KEGG_ANTIGEN_PROCESSING_AND_PRESENTATION	0.816	2.385	0.000	0.000
KEGG	KEGG_CYTOKINE_CYTOKINE_RECEPTOR_INTERACTION	0.661	2.376	0.000	0.000
KEGG	KEGG_RIG_I_LIKE_RECEPTOR_SIGNALING_PATHWAY	0.703	2.358	0.000	0.000
KEGG	KEGG_LEISHMANIA_INFECTION	0.830	2.348	0.000	0.000
KEGG	KEGG_AUTOIMMUNE_THYROID_DISEASE	0.840	2.292	0.000	0.000
KEGG	KEGG_INTESTINAL_IMMUNE_NETWORK_FOR_IGA_PRODUCTION	0.868	2.289	0.000	0.000
KEGG	KEGG_LYSOSOME	0.658	2.285	0.000	0.000
KEGG	KEGG_CELL_ADHESION_MOLECULES_CAMS	0.698	2.285	0.000	0.000
KEGG	KEGG_B_CELL_RECEPTOR_SIGNALING_PATHWAY	0.683	2.281	0.000	0.000
KEGG	KEGG_HEMATOPOIETIC_CELL_LINEAGE	0.728	2.276	0.000	0.000
KEGG	KEGG_VIRAL_MYOCARDITIS	0.719	2.260	0.000	0.000
KEGG	KEGG_PRIMARY_IMMUNODEFICIENCY	0.867	2.235	0.000	0.000
KEGG	KEGG_T_CELL_RECEPTOR_SIGNALING_PATHWAY	0.676	2.233	0.000	0.000
KEGG	KEGG_PROTEASOME	0.810	2.202	0.000	0.000
KEGG	KEGG_TYPE_I_DIABETES_MELLITUS	0.868	2.197	0.000	0.000
KEGG	KEGG_EPITHELIAL_CELL_SIGNALING_IN_HELICOBACTER_PYLORI_INFECTION	0.615	2.148	0.000	0.000
KEGG	KEGG_JAK_STAT_SIGNALING_PATHWAY	0.579	2.120	0.000	0.000
KEGG	KEGG_ALLOGRAFT_REJECTION	0.918	2.076	0.000	0.001

Gene sets	Description	ES	NES	pvalue	FDR
KEGG	KEGG_ASTHMA	0.833	2.069	0.000	0.001
KEGG	KEGG_APOPTOSIS	0.582	2.064	0.000	0.001
KEGG	KEGG_AMINO_SUGAR_AND_NUCLEOTIDE_SUGAR_METABOLISM	0.642	2.054	0.000	0.001
KEGG	KEGG_GRAFT_VERSUS_HOST_DISEASE	0.890	2.048	0.000	0.001
KEGG	KEGG_FC_GAMMA_R_MEDIATED_PHAGOCYTOSIS	0.567	1.997	0.002	0.002
KEGG	KEGG_CELL_CYCLE	0.592	1.966	0.000	0.003
KEGG	KEGG_PRION_DISEASES	0.593	1.915	0.000	0.007
KEGG	KEGG_CYSTEINE_AND_METHIONINE_METABOLISM	0.571	1.889	0.000	0.010
KEGG	KEGG_LEUKOCYTE_TRANSENDOTHELIAL_MIGRATION	0.526	1.887	0.004	0.010
KEGG	KEGG_SYSTEMIC_LUPUS_ERYTHEMATOSUS	0.607	1.872	0.004	0.011
KEGG	KEGG_PANCREATIC_CANCER	0.530	1.837	0.004	0.017
KEGG	KEGG_ENDOCYTOSIS	0.475	1.825	0.004	0.019
KEGG	KEGG_NICOTINATE_AND_NICOTINAMIDE_METABOLISM	0.577	1.799	0.004	0.023
KEGG	KEGG_FC_EPSILON_RI_SIGNALING_PATHWAY	0.491	1.797	0.008	0.023
KEGG	KEGG_SNARE_INTERACTIONS_IN_VESICULAR_TRANSPORT	0.535	1.784	0.011	0.025
KEGG	KEGG_ACUTE_MYELOID_LEUKEMIA	0.526	1.780	0.008	0.026
KEGG	KEGG_PYRIMIDINE_METABOLISM	0.472	1.770	0.010	0.028
KEGG	KEGG_P53_SIGNALING_PATHWAY	0.482	1.767	0.010	0.028
KEGG	KEGG_PATHOGENIC_ESCHERICHIA_COLI_INFECTION	0.511	1.761	0.012	0.029
KEGG	KEGG_VIBRIO_CHOLERAE_INFECTION	0.490	1.744	0.008	0.033
KEGG	KEGG_OOCYTE_MEIOSIS	0.474	1.743	0.012	0.033
KEGG	KEGG_UBIQUITIN_MEDIATED_PROTEOLYSIS	0.453	1.691	0.015	0.048
HALLMARK	HALLMARK_INTERFERON_GAMMA_RESPONSE	0.885	2.456	0.000	0.000
HALLMARK	HALLMARK_IL6_JAK_STAT3_SIGNALING	0.821	2.447	0.000	0.000
HALLMARK	HALLMARK_COMPLEMENT	0.685	2.417	0.000	0.000
HALLMARK	HALLMARK_ALLOGRAFT_REJECTION	0.823	2.409	0.000	0.000
HALLMARK	HALLMARK_INFLAMMATORY_RESPONSE	0.771	2.388	0.000	0.000
HALLMARK	HALLMARK_UV_RESPONSE_UP	0.588	2.309	0.000	0.000
HALLMARK	HALLMARK_TNFA_SIGNALING_VIA_NFKB	0.734	2.299	0.000	0.000
HALLMARK	HALLMARK_MTORC1_SIGNALING	0.659	2.292	0.000	0.000
HALLMARK	HALLMARK_IL2_STAT5_SIGNALING	0.617	2.288	0.000	0.000

Gene sets	Description	ES	NES	pvalue	FDR
HALLMARK	HALLMARK_INTERFERON_ALPHA_RESPONSE	0.919	2.269	0.000	0.000
HALLMARK	HALLMARK_APOPTOSIS	0.591	2.244	0.000	0.000
HALLMARK	HALLMARK_PI3K_AKT_MTOR_SIGNALING	0.588	2.144	0.000	0.000
HALLMARK	HALLMARK_UNFOLDED_PROTEIN_RESPONSE	0.557	2.075	0.002	0.001
HALLMARK	HALLMARK_KRAS_SIGNALING_UP	0.539	1.953	0.000	0.004
HALLMARK	HALLMARK_E2F_TARGETS	0.680	1.940	0.006	0.004
HALLMARK	HALLMARK_G2M_CHECKPOINT	0.633	1.934	0.006	0.004
HALLMARK	HALLMARK_REACTIVE_OXYGEN_SPECIES_PATHWAY	0.577	1.869	0.004	0.009
HALLMARK	HALLMARK_P53_PATHWAY	0.442	1.818	0.002	0.015
HALLMARK	HALLMARK_GLYCOLYSIS	0.463	1.797	0.004	0.017
HALLMARK	HALLMARK_ANDROGEN_RESPONSE	0.484	1.751	0.011	0.025
HALLMARK	HALLMARK_XENOBIOTIC_METABOLISM	0.431	1.745	0.006	0.024
HALLMARK	HALLMARK_FATTY_ACID_METABOLISM	0.467	1.711	0.010	0.031
HALLMARK	HALLMARK_PROTEIN_SECRETION	0.505	1.705	0.025	0.031
HALLMARK	HALLMARK_CHOLESTEROL_HOMEOSTASIS	0.488	1.705	0.026	0.030
HALLMARK	HALLMARK_HYPOXIA	0.453	1.703	0.021	0.029
HALLMARK	HALLMARK_DNA_REPAIR	0.455	1.659	0.028	0.037

Table S5. Differentially mutated genes between high- and low-APOBEC3A expression groups.

Hugo_Symbol	High.APOBEC3A	Low.APOBEC3A	P.value	OR	CI.up	CI.low
PLCH1	12	1	0.003	12.703	549.635	1.829
ATG9A	8	0	0.007	Inf	Inf	1.726
MYO16	8	0	0.007	Inf	Inf	1.726
ZSWIM8	8	0	0.007	Inf	Inf	1.726
TSHZ3	12	2	0.011	6.310	59.236	1.363
C19orf44	7	0	0.014	Inf	Inf	1.448
GIGYF2	7	0	0.014	Inf	Inf	1.448
MAN2A2	7	0	0.014	Inf	Inf	1.448
PAPPA2	7	0	0.014	Inf	Inf	1.448
SIDT2	7	0	0.014	Inf	Inf	1.448
TRIO	7	0	0.014	Inf	Inf	1.448
PRUNE2	18	6	0.017	3.221	10.268	1.173
FAT4	2	10	0.019	0.184	0.890	0.019
HIVEP3	9	1	0.019	9.303	412.708	1.257
COL24A1	0	5	0.028	0.000	1.051	0.000
DHX34	0	5	0.028	0.000	1.051	0.000
MALT1	0	5	0.028	0.000	1.051	0.000

Hugo_Symbol	High.APOBEC3A	Low.APOBEC3A	P.value	OR	CI.up	CI.low
PPFIA1	0	5	0.028	0.000	1.051	0.000
TMPRSS2	0	5	0.028	0.000	1.051	0.000
ZNF101	0	5	0.028	0.000	1.051	0.000
CDH5	6	0	0.030	Inf	Inf	1.175
CERCAM	6	0	0.030	Inf	Inf	1.175
COL11A1	6	0	0.030	Inf	Inf	1.175
COL14A1	6	0	0.030	Inf	Inf	1.175
COL5A1	6	0	0.030	Inf	Inf	1.175
IRF8	6	0	0.030	Inf	Inf	1.175
ITIH1	6	0	0.030	Inf	Inf	1.175
LRP4	6	0	0.030	Inf	Inf	1.175
MC5R	6	0	0.030	Inf	Inf	1.175
NRXN1	6	0	0.030	Inf	Inf	1.175
PCDHB5	6	0	0.030	Inf	Inf	1.175
PPP1R15A	6	0	0.030	Inf	Inf	1.175
SLC9A4	6	0	0.030	Inf	Inf	1.175
SVIL	10	2	0.034	5.175	49.516	1.072
KANK1	1	7	0.035	0.134	1.067	0.003
NUMA1	1	7	0.035	0.134	1.067	0.003
DMD	8	1	0.036	8.205	368.488	1.074

1.2 Supplementary Figures

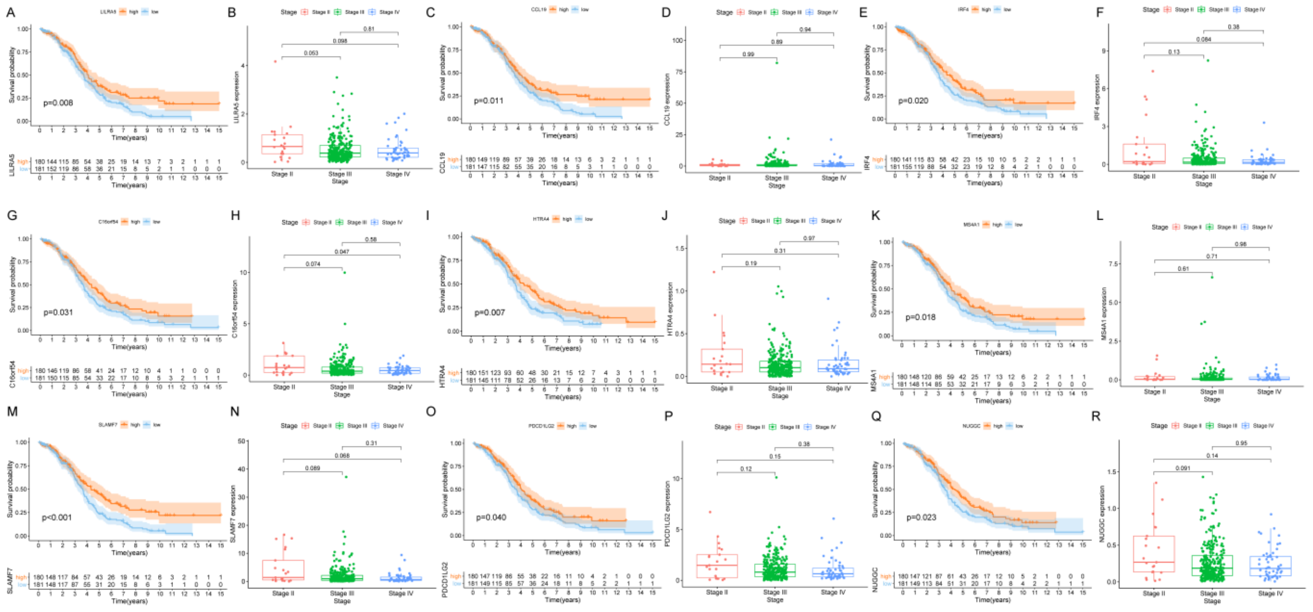

Supplementary Figure 1. Correlation analysis of intersected DEGs with survival and clinicopathological characteristics of ovarian cancer (OC) patients. (A–C) Distribution of gene expression in stage, by Kruskal–Wallis rank sum test. (D–F) Distribution of three kinds of scores in T

classification (p = 0.5, 0.84, 0.55 for ImmuneScore, StromalScore, and ESTIMATEScore, respectively, by Kruskal–Wallis rank sum test). (G–I) Distribution of scores in M classification (p = 0.015, 0.014, 0.004 for ImmuneScore, StromalScore, and ESTIMATEScore separately by Wilcoxon rank sum test). (J–L) Distribution of scores in N classification. Similar to the preceding, p = 0.56, 0.27, 0.40, respectively, with Wilcoxon rank sum test.
